# Identification of Novel β-Tubulin Inhibitors Using a Combined *In Silico*/*In Vitro* Approach

**DOI:** 10.1021/acs.jcim.3c00939

**Published:** 2023-09-29

**Authors:** Mark James Horgan, Lukas Zell, Bianka Siewert, Hermann Stuppner, Daniela Schuster, Veronika Temml

**Affiliations:** † Institute of Pharmacy/Pharmacognosy, Center for Chemistry and Biomedicine, University of Innsbruck, Innrain 80-82, 6020 Innsbruck, Austria; ‡ Institute of Pharmacy, Department of Pharmaceutical and Medicinal Chemistry, Paracelsus Medical University Salzburg, Strubergasse 21, 5020 Salzburg, Austria

## Abstract

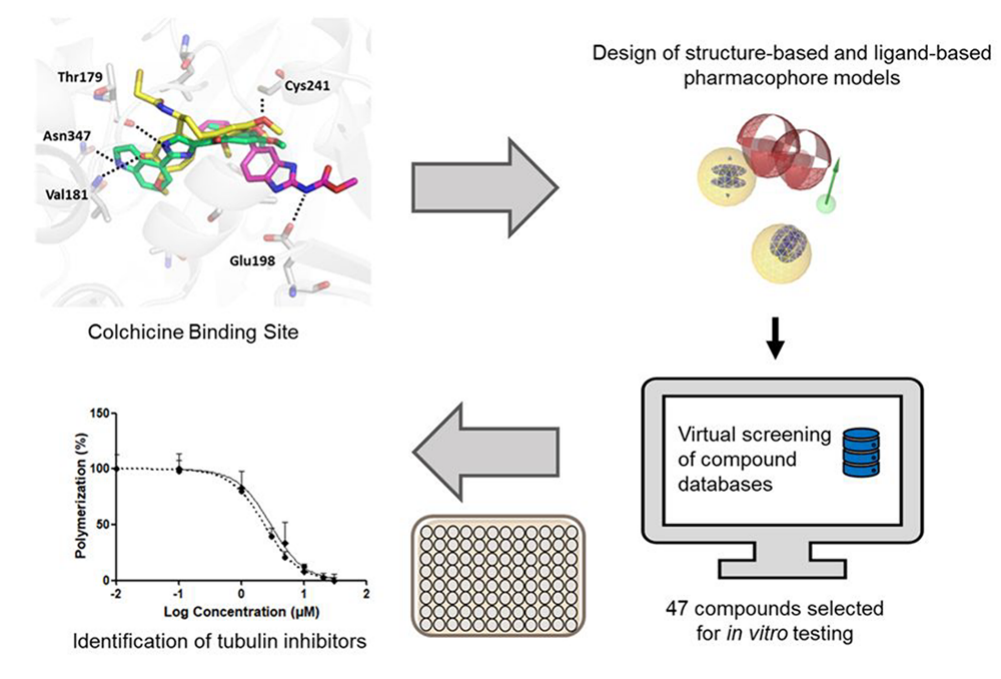

Due to their potential as leads for various therapeutic applications, including as antimitotic and antiparasitic agents, the development of tubulin inhibitors offers promise for drug discovery. In this study, an *in silico* pharmacophore-based virtual screening approach targeting the colchicine binding site of β-tubulin was employed. Several structure- and ligand-based models for known tubulin inhibitors were generated. Compound databases were virtually screened against the models, and prioritized hits from the SPECS compound library were tested in an *in vitro* tubulin polymerization inhibition assay for their experimental validation. Out of the 41 SPECS compounds tested, 11 were active tubulin polymerization inhibitors, leading to a prospective true positive hit rate of 26.8%. Two novel inhibitors displayed IC_50_ values in the range of colchicine. The most potent of which was a novel acetamide-bridged benzodiazepine/benzimidazole derivative with an IC_50_ = 2.9 μM. The screening workflow led to the identification of diverse inhibitors active at the tubulin colchicine binding site. Thus, the pharmacophore models show promise as valuable tools for the discovery of compounds and as potential leads for the development of cancer therapeutic agents.

## 1 Introduction

**Figure 1:**
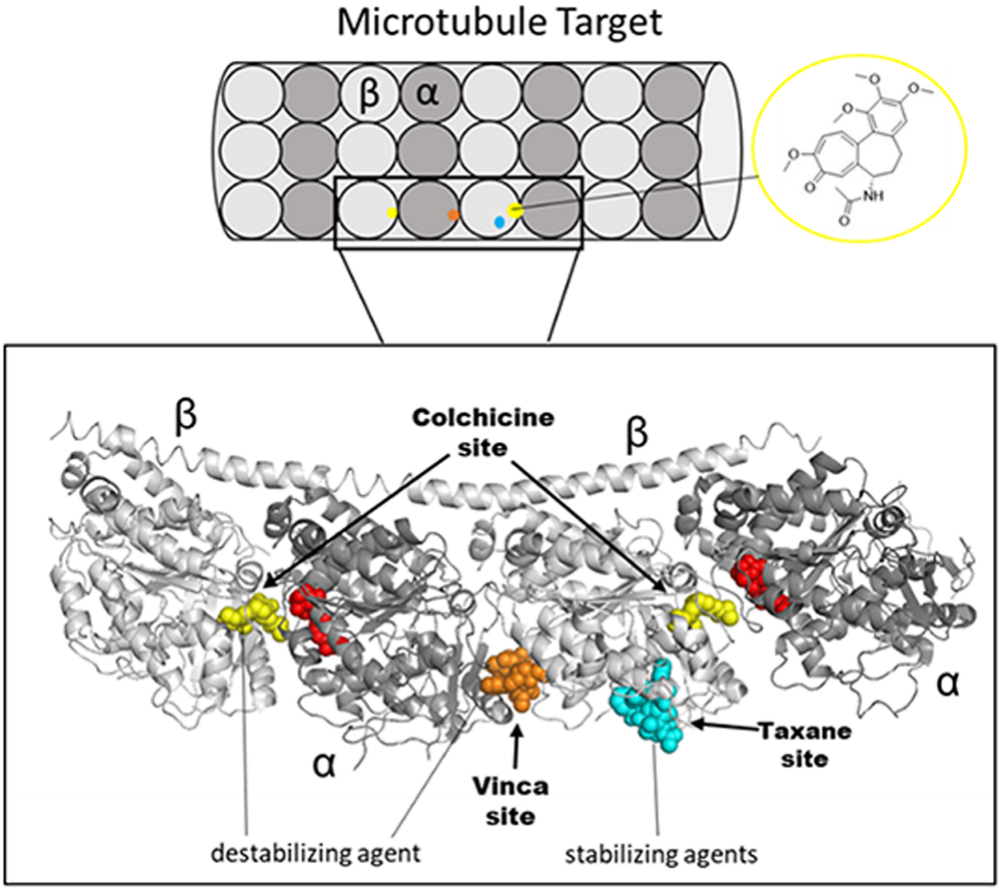
Graphical depiction of microtubule target and α/β-tubulin monomer units with 3D structural overlay (gray) with antimitotic ligands cocrystallized in their respective binding pockets highlighted. Taxol complex (blue, PDB: 1JFF)^9^ with the vinblastine complex (orange, PDB: 1Z2B)^10^ superimposed onto the (yellow, PDB: 1SA0)^11^ colchicine-DAMA complex. red, GTP (Guanosine-5′-triphosphate) substrate. Graphic made in PyMOL.^12^

Cancer is defined as uncontrolled proliferation of cells, resulting in the formation of a tumor.^1^ The number of cancer deaths is escalating, making it one of the leading causes of deaths across several demographics and age groups with alarming projections.^2^ Therefore, there is an urgent need for new effective and rapidly approved anticancer agents. However, the success rate for the approval of new drugs is limited. Clinical failures and stagnation is prevalent from phase I to eventual commercial use, with the U.S. Food and Drug Administration (FDA) approval taking around 8.3 years with an estimated 6.7% success rate.^3,4^ Microtubule disrupters, known as antimitotic agents, are an established class of chemotherapeutics in this complex therapy area. The abnormal growth of malignant tumors is characterized by uncontrolled rapid cell division with unlimited replicative potential.^5^ Thus, these drugs target the microtubule proteins involved in spindle fiber formation and inhibit replication.^6^ Specifically, the microtubule polymers composed of α/β-tubulin heterodimers play a crucial role in the mitosis phase.^7^ Classically, inhibitors act as microtubule-destabilizing or-stabilizing agents, disrupting mitosis and mechanistically exerting their effect by binding at the taxol, vinca, or colchicine binding site (CBS) (Figure 1), although at least two more α/β-tubulin binding sites have been allocated and studied.^8^

**Figure 2:**
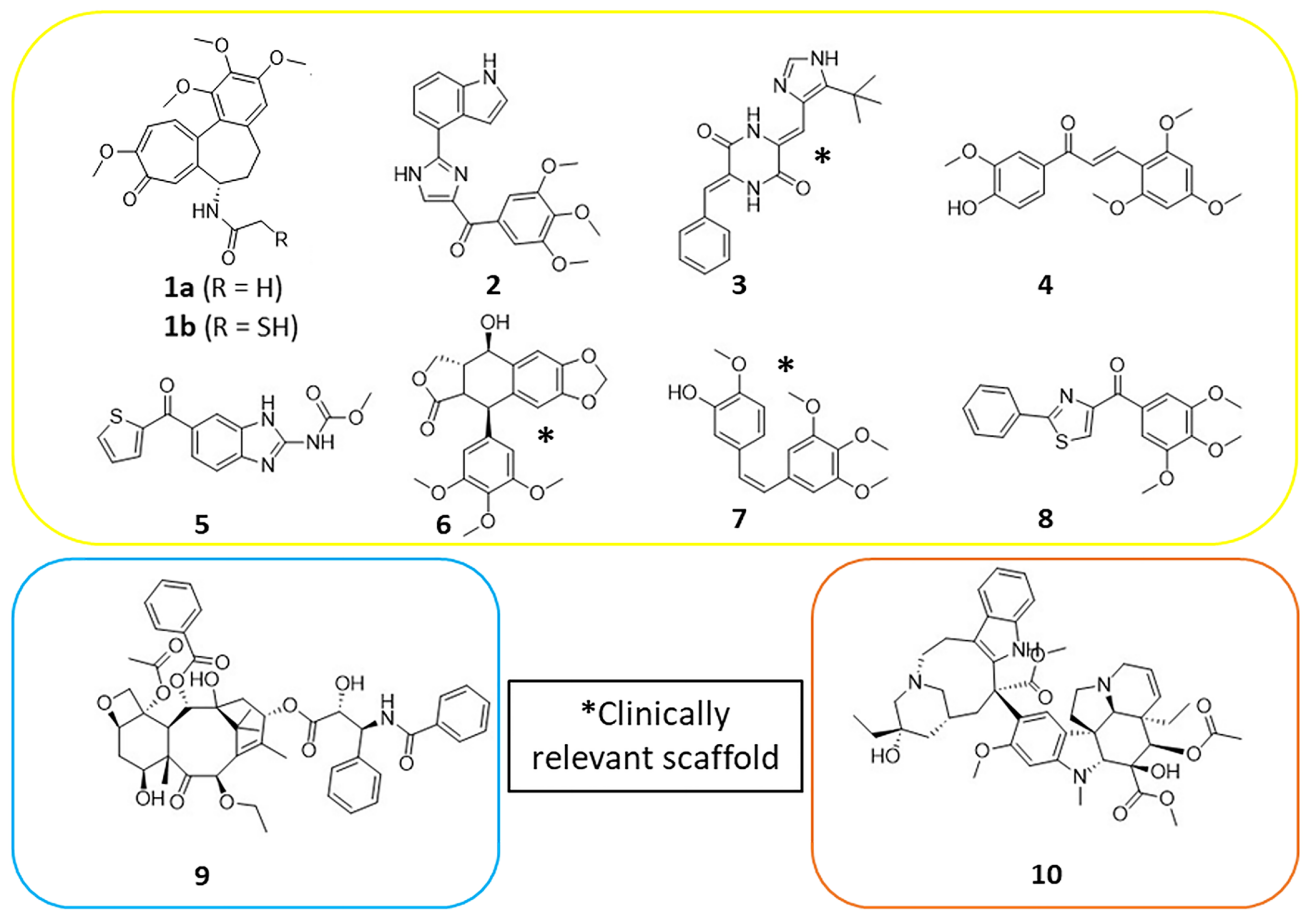
Overview of microtubule inhibitors that target different binding sites of tubulin.

An overview of inhibitors targeting the different binding sites of tubulin is shown (Figure 2). Notably, CBS inhibitors (CBSIs) with natural and synthetic scaffolds exist as microtubule-destabilizing agents and exert their effect by binding at the β-tubulin colchicine domain.^13^ The CBSIs (**1**–**8**) are considered powerful antimitotic agents, but colchicine’s (**1a**) clinical application was limited due to its severe toxicity. It also binds to the tubulin of noncancerous cells, causing mitotic arrest and impairing protein assembly in healthy cells, leading to organ dysfunction.^14^ Furthermore, a **1a** analogue prodrug for the treatment of solid renal tumors was terminated after phase I trials, due to GI and cardiac side effects.^15^ Despite being prescribed in Europe as a second line treatment for gout,^16^ its lack of success as a cancer therapeutic is correlated with the fact that there are currently no European Medicines Agency (EMA) or FDA marketed cancer drugs targeting the CBS. Instead, patients have been treated with the natural product (NP)-derived taxane and vinca alkaloids, including taxol **9** and vinblastine **10**, respectively. Taxol is primarily indicated for Kaposi’s sarcoma, lung, ovarian, and breast cancer; meanwhile, vinblastine is used to treat malignant lymphomas, Hodgkin’s disease, breast, and testicular cancers.^17^ Both are flagship drugs for the success of microtubule-disrupting agents in cancer chemotherapy.

Despite therapeutic limitations, motivation remains for investigating CBSIs, as the approved taxane and vinca alkaloids have notable drawbacks, such as intravenous administration due to poor aqueous solubility, affecting patient compliance. Their high lipophilicity also requires the use of surfactants, causing reactions and hypersensitivity in patients.^18,19^ Alarmingly, the prevalence of multidrug resistance (MDR) in tumors limits the effectiveness of cytotoxic agents, leading to treatment failure. Such resistance mechanisms include the overexpression of P-glycoprotein (P-gp) efflux pumps decreasing intracellular drug concentrations and the mutant β-tubulin III isoform.^20−22^ Currently, models suggest that CBSIs might remain unaffected by this β-tubulin mutation.^20^ Additionally, the high cost of alternative monoclonal antibody biologics is another economic incentive. Research efforts have been led to find alternative CBSIs for anticancer therapy with numerous natural, semisynthetic, or synthetic compounds being reported. The investigations into CBSIs are prevalent, and comprehensive literature reviews outlining the importance of these scaffolds from preclinical to clinical development have been performed.^13,23^ Many NP scaffolds remain excellent starting points for discovering novel CBSIs. Isolated from *Popdophyllum* rhizomes, Podophyllotoxin **6** had its antimitotic effects reported in similar fashion to **1a**.^24^ However, development for use in chemotherapy was impeded due to its unfavorable toxicity profile.^25^ Optimization of **6** led to a series of semisynthetic derivatives, including etoposide, approved to treat a number of cancers by the EMA and FDA.^26^ Despite binding to the CBS of β-tubulin, this analogue has a distinct mode of action to **6**, with DNA topoisomerase II as its primary cytotoxic target.^27^ Concerningly, complications of routine use emerged with the onset of secondary leukemia in patients.^28^ The less toxic phosphate prodrug analogue etopophos with improved formulation characteristics has largely displaced etoposide in clinical settings.^29,30^ Another NP, combretastatin A-4 **7**, isolated from *Combretum caffrum* plants traditionally used by the South African Xhosa tribe, is a CBS inhibitor with potent cytotoxicity against cancer cell lines.^31−33^ A notable bioactivity feature was the retention of the trimethoxyphenyl (TMP) ring substituent, analogous to **1a**. As emphasized in the mentioned reviews, medicinal chemistry efforts yielded unquestionably potent **7** analogues with improved pharmacokinetics. However, they were overshadowed by poor aqueous solubility and short half-life, requiring introduction of heterocycles and/or a phosphate or amino acid hydrochloride salt.^19^ This challenge was circumvented by effective prodrug strategies with improvements in the therapeutic potential of this scaffold, and clinical candidates now reside in phases I–III.^23^ Subsequent modifications utilized the chalcone **4** scaffold to design further derivatives of **7**. The simplicity of the **4** scaffold was favorable for further CBS analogue design with potent antimitotic activities summarized by Dong et al.^13^ More recently, the marine halimide, plinabulin **3** used with docetaxel, is now under phase III trials for epidermal growth factor receptor wild-type patients with its activity owed to CBS interactions.^23,34^ Moreover, NP scaffolds and semisynthetics are not self-standing as synthetic scaffolds have also been prioritized in investigations. Notable synthetic ligands for *in vitro* development of CBSIs include heterocycles (myoseverin, thiazolidinone) and sulfonamides.^13^ One widely studied heterocycle, nocodazole **5**, gave crucial information into the role of CBSIs in microtubule dynamics at the cell biology level.^35^ Established benzimidazoles with antimitotic mechanisms against human and veterinary parasites support their wider safe use as medications.^36,37^ A recent prodrug lisavanbulin with a benzimidazole moiety, reached phase I/IIa trials for advanced solid tumors.^38^ Interestingly, the synthetic indole **2** demonstrated potent tumor growth inhibition in a taxane resistant model, suggesting it might circumvent certain types of MDR that taxanes cannot evade.^39^ Meanwhile, analogues of the synthetic thiazole scaffold **8** also showed promise for overcoming P-gp mediated MDR.^40^ The onset of MDR against the vinca- and taxol-derived drugs highlights the immediate clinical relevance of both NP-derived and synthetic CBSIs toward antimitotic discovery.

**Figure 3:**
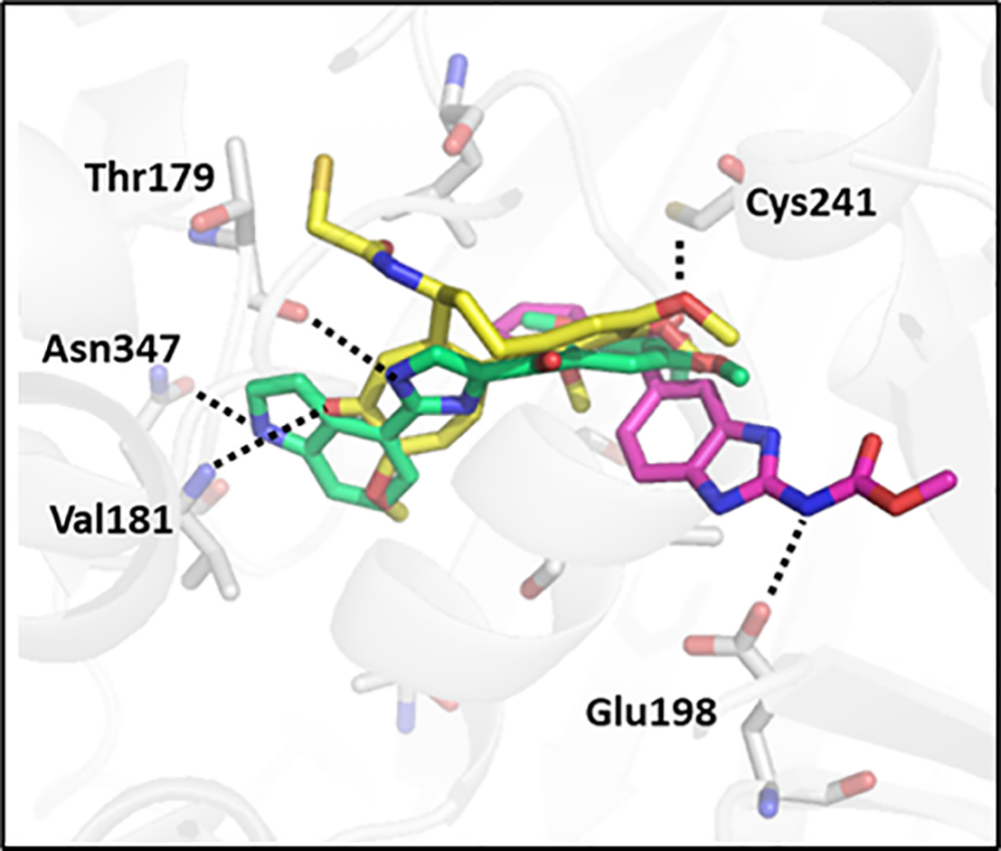
3D graphic of the CBS by overlaying the crystal structures (PDB: 1SA0, 5CA1, and 6O5M)^11,34,39^ in complex with the ligands **1b** (yellow, sticks), **3** (green, sticks), and **5** (magenta, sticks) showing polar interactions with amino acid residues αThr179, αVal181, βCys241, βAsn347, and βGlu198 (gray, sticks). Made in PyMOL.^12^

As drug design assets, molecular modeling studies have been performed based on the interaction of ligands at the CBS. Important crystallographic structures mapping binding interaction complexes are presented in Figure 3. Ravelli et al. first described the CBS buried between the β/α-subunits (PDB: 1SA0) with the DAMA–colchicine in complex.^11^ The βCys-241 residue forms a hydrogen bond with the TMP of **1b** while αThr-179 and αVal-181 form hydrogen bonds with the tropolone ring. Later, Wang et al. showed the structure ligand complex (PDB: 5CA1) and proposed a structure-based (SB) pharmacophore of tubulin with **5**, reporting its key CBS interactions overlap little with **1a**, forming hydrogen bonds with βAsn-165 and βGlu-198.^34^ In contrast, the indole **2** bound at the CBS showed greater overlap with **1a** (PDB: 6O5M).^39^ The ligand shares hydrogen bond interactions with αThr-179 and βAsp-249. In addition, a hydrogen bond between the indole amine and carbonyl of β-Asn347 was observed, and a separate water-mediated hydrogen bond network was observed between the middle methoxy oxygen to the backbone amine in βCys-239 and carbonyl in βGly-235. They declared that this water-bridged feature was likely responsible for increased activity compared to analogues. Such reports rationalize the distinct and ubiquitous interactions at the CBS. Abundant ligand and target information provides a base for CBS pharmacophore modeling.

A pharmacophore is a set of common electronic and geometric features required for a ligand to interact with amino acid residues of a given protein target exerting a biological response.^41^ Such features may consist of positive/negative ionizable interactions, hydrophobic contacts (HC), aromatic interactions (AI), and hydrogen bond donors/acceptors (HBD/HBA) for the interaction with the target. Pharmacophore-based virtual screening is an established computational lead discovery tool utilized in modern drug development.^42^ Co-utilizing SB and ligand-based (LB) screening exploits the target’s 3D structure and a ligand’s molecular similarity principle to cover more of the chemically active space.^43^ It has also been demonstrated that using several modeling programmes for model hit prediction is advantageous.^44^ Moreover, workflows employing both LigandScout (LS) and Discovery Studio (DS) software with *in vitro* validation of potential lead candidates has been successfully applied.^45−47^ Meanwhile pharmacophore models have been developed in CBS antimitotic discovery efforts.^48−50^ However, to our knowledge, none have yet integrated a combined SB-LB pharmacophore workflow with more than one modeling software coupled with experimental validation. Coupled LB pharmacophore modeling allows for inclusion of features from inhibitors where the exact binding mode is not elucidated in crystallography. Thus, providing an opportunity for its application in CBS investigations for anticancer chemotherapy and immunomodulation as outlined in this paper.

## 2 Materials and Methods

### 2.1 Data Set Assembly

A literature search was performed for known β-tubulin CBS inhibitors, and a set of active compounds (*n* = 95) was curated. The compounds were selected based on their scaffold chemical diversity from natural, semisynthetic, and synthetic origin and with inhibition activity reports and IC_50_ values in the micromolar range (<100 μM). A list detailing the selected inhibitors is given in the Supporting Information (SI) (Section 1, Table S1).

A corresponding decoy set was developed from the ChEMBL database (version 26, accessed 05/08/2020) by selecting the distinct compounds search function, followed by selection and download of the “all small molecules” file (*n* ≥ 2 million). Next, a customized script in Pipeline Pilot 2019 Client (BIOVIA, San Diego)^51^ was used for clustering and filtering. The actives data set SMILES codes were input to the script file reader. The script function calculated the mean and SD of the active data set’s physiochemical properties (molecular weight, #N, #O atoms, #rotatable bonds, #HBD, #HBA, and LogP values), and Pipeline Pilot was set to filter those within range from lowest to highest occurring in the database. Thus, the ChEMBL database compounds were reduced to a selected subset of compounds with similar physiochemical properties to the actives set. Furthermore, known tubulin active molecules (from ChEMBL) were removed from the subset (*n* = 1 million), and the remaining compounds were clustered to arrive at a final decoy data set of 4901 structurally diverse compounds with similar physiochemical properties to the actives set. These random compounds are assumed to be inactive for modeling purposes.^52^

Prior to model training, both data sets were converted into multiconformational screening databases with a LS (www.inteligand.com)^53^ integrated Omega conformer generator (https://www.eyesopen.com/omega)^54^ using default “FAST” settings (calculating a maximum of 25 conformers for each structure). For the models generated in DS, the algorithm calculates a maximum of 255 conformers under “FAST” settings.^51^ This differs from Omega but is equivalent since it uses a different conformer generator that allows more similar conformers. The generated databases were used for model training and theoretical validation of the generated pharmacophore models.^52^

### 2.2 Pharmacophore Model Generation and Evaluation

To optimize mapping of the inhibitor’s active chemical space, the pharmacophore modeling programs, LS version 4.08^53^ and DS version 3.0 (BIOVIA),^51^ were employed. For SB modeling, X-ray crystal structures of the protein–ligand complexes (PDB: 5CA1)^34^ and (PDB: 6O5M)^39^ were obtained from the Protein Data Bank (PDB).^55^ For LB modeling in LS, either the merged or shared feature mode was used for the 3D alignment of the selected molecules in the actives data set. All models were subsequently trained against subsets of the actives/decoy data sets. Each automatically generated model possessed a variety of different pharmacophore features such as HBD/A, HC, and AI. Additionally, they contained exclusion volumes (Xvols), which prohibit steric clashes of the molecule with the protein. To optimize the automatically generated models, each model was manually refined or altered during subsequent screening steps. Features that did not lead to higher model selectivity were removed, Xvols were added/removed, and feature tolerances adjusted to optimize model performance. The calculated quality metrics included sensitivity (eq 1), specificity (eq 2), accuracy (eq 3), yield of actives (YoA) (eq 4), and enrichment factor (EF) (eq 5). This refinement process was guided by continuous observation of the quality enrichment metrics calculated during each step as described in previous reports.^47^ Pharmacophore models which failed to reach good performance (i.e., EF < 4) were discarded. Graphical depictions of the final optimized models and detailed descriptions outlining their individual optimization and features can be found in the SI (Section 2, Figures S1–S16) .
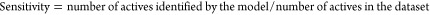
1
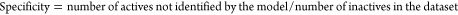
2
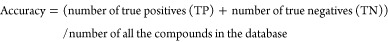
3
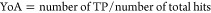
4
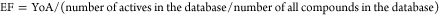
5

### 2.3 Virtual Screening

The 16 generated and optimized pharmacophore models (models 1–16) were subjected to virtual screening against three distinct databases: (1) SPECS database of commercially available synthetic compounds (*n* = 208,968) and (2) SPECS (NP) (*n* = 736) downloaded from www.specs.net (accessed May 2021; Specs_SC_10 mg_May2021, Specs_NP_1 mg_May2021). (3) Further, an in-house, manually curated polyphenol database named PhytChem (PC) (*n* = 735) was used. Each database was prepared for virtual screening by creating 3D multiconformational databases using the Omega conformer generator^54^ with default “FAST” settings calculating a maximum of 25 conformers in LS; meanwhile in DS, the algorithm calculated a maximum of 255 conformers under “FAST” settings for each screening database.^51^

### 2.4 Filtering and Selection of Test Compounds for Biological Assays

In order to guide selection for biological testing, virtual hits obtained were subject to a filtering workflow with multiple cutoff steps. Hit selection was guided by ranking compounds based on consensus overlap and pharmacophore fit scores/values. The online server SwissADME (www.swissadme.ch, accessed May 2021)^56^ was used to prioritize selection further based on Lipinski’s properties (solubility) and alerts to any PAINS or Brenk violations.^57,58^ A final visual inspection was performed to limit and remove chemically unstable functional groups and assess structural similarities. The final hit list (*n* = 47) was cross checked with SciFinder for previously known tubulin activity. Out of those hit candidates which met the criteria, 46 commercially available compounds (**SC1**-**SC46**) (10 hits from SPECS NP and 36 from hits SPECS database) were purchased from SPECS chemicals (www.specs.net), and one (**Hit47**) (hit from PC database) was purchased from MERCK Group.

### 2.5 Fluorescence-Based Cell-Free Tubulin Polymerization Assay

The assay test kit, Tubulin polymerization HTS assay using >99% pure tubulin, fluorescence based (#BK011P), was purchased from Cytoskeleton, Inc. (www.cytoskeleton.com).^59^ The fluorescence-based tubulin polymerization experiment was developed in-line with the kit manual applying some minor modifications. Each of the kit components were reconstituted as solutions and stored as described in the kit guide.^59^ To prepare the tubulin stock, lyophilized brain tubulin powder (>99%, porcine, 10 mg) was placed on ice. The tubulin powder was resuspended in the supplied supplemented buffer 1 (1.1 mL) and kept on ice for 2 min to ensure complete resuspension. On ice, the tubulin stock (10 mg/mL, 88 μL) was dispensed as aliquots into labeled 0.5 mL Eppendorf tubes and snap-frozen with liquid nitrogen. The tubulin stock was stored at −80 °C until later use. To prepare the tubulin reaction mix for each assay, the GTP stock solution (100 mM, 20 μL) and buffer 1 (1.5 mL) were thawed and placed on ice. Glycerol buffer was then removed from +4 °C and placed on ice. Next, the tubulin stock was thawed and immediately placed on ice, and the mix components were combined as follows: buffer 1 (205 mL), glycerol buffer (150 μL), GTP stock solution (100 mM, 4.4 μL), and tubulin stock (10 mg/mL, 85 μL) and kept on ice. Test compounds and control solutions were prepared by dissolving samples in DMSO (3 mM), and from this, aqueous stock solutions (300 μM) were prepared by adding the DMSO/compound stock (100 μL) to Milli-Q water (900 μL).

For screening, 5 μL of each stock solution was added to separate wells of the assay plate (Cytoskeleton Inc., Denver, CO, USA; half area 96-well plate, black, flat bottom) with final DMSO concentrations (<2%). The 96-well plate was submitted to a Tecan Spark 10 M plate reader (Tecan, Männedorf, Switzerland) and warmed for 1 min to 37 °C. Then, 50 μL of the tubulin solution was pipetted into each well. Final test compound concentration in the wells was 30 μM. The fluorimeter function equipped with filters was preset to excitation at 340 nm (20 nm bandwidth), and the monochromator was set to emission at 450 nm. The gain was manually set to 40 (mirror, flashes = 30, integration time = 40 μs, lag time = 0 μs). The reaction plate was resubmitted to the temperature-controlled plate reader (*t* = 60 min, *T* = 37 °C). The polymerization inhibitor **1a** colchicine (TCI Chemicals) was used as a positive control. For bioactivity data analysis, significance testing by one sample *t* test and IC_50_ calculations with nonlinear regression (curve fit), log (inhibitor) vs normalized response–variable slope were performed using GraphPad Prism version 5.01 for Windows (GraphPad Software, San Diego, California USA, www.graphpad.com).^60^

## 3 Results

### 3.1 General Workflow

**Figure 4:**
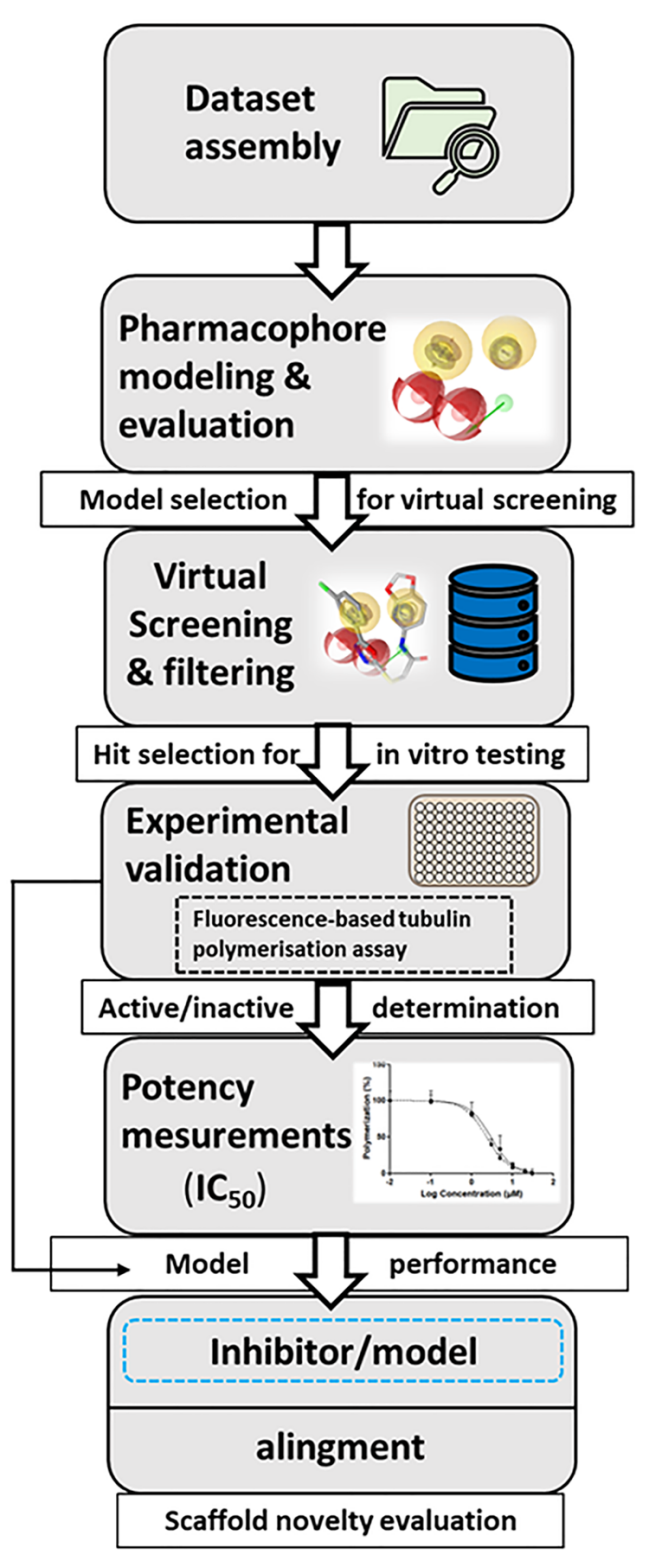
General workflow for the pharmacophore model-based search for novel tubulin CBS inhibitors.

The general workflow of this study is summarized in Figure 4. The PDB database was searched for ligand-binding complexes of tubulin CBS. A total of 95 known CBS inhibitors from the literature were curated into a data set of active ligands. Pharmacophore SB models were created from two PDB structures and the remaining built LB models from the actives set. The initial models were optimized to map as many actives and reject as many decoys as possible. The final models were then used for the virtual screening of SPECS and in-house compound databases. Virtual hits were filtered and prioritized for *in vitro* screening. Test compounds were purchased and classified as active or inactive based on *in vitro* screening at maximum water solubility. The strongest inhibitors identified were subject to concentration–response experiments with potency (IC_50_) determined. Finally, the strongest inhibitors were aligned to their respective models to evaluate their potential pharmacophore binding features and amino acid interactions.

### 3.2 Data Set Assembly

The selected ligands were composed of a variety of chemical scaffolds from both synthetic and NP origin. They were selected on the basis of their known tubulin inhibition and scaffold diversity to ensure sufficient mapping of the available bioactive space of the target.^61^ The ligands were analogues and derivatives of the following CBSI scaffolds: benzimidazole, thiazole, myoseverin, thiazolidinone, sulfonamide, arylthioindoles, anthracenone, combretastatin, podophyllotoxin, chalcone, sesquiterpenoids, and colchicine alkaloids. A detailed list of the selected tubulin CBS inhibitors, abbreviated with **AS** (active set) and listed **AS1**–**AS95**, is shown in the SI (Section 1, Table S1).

### 3.3 Pharmacophore Modeling and Evaluation

Graphical depictions of the final optimized models and detailed descriptions outlining their individual optimization and features can be found in the SI (Section 2, Figures S1–S16). The automatically generated SB and LB pharmacophore models were individually optimized, and below the individual final models are briefly described.

**Figure 5:**
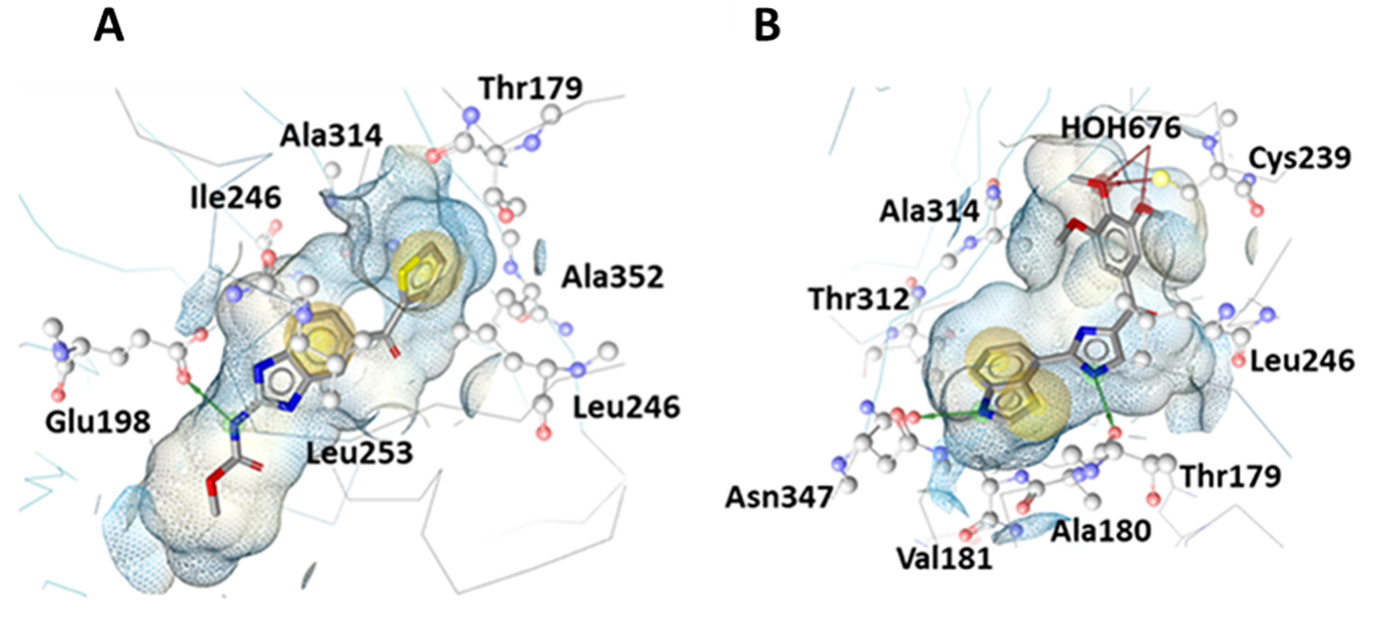
3D representations of tubulin-ligand complexes used to create SB pharmacophore models generated with LS. Automatically generated models (A) based on coordinates the X-ray crystal structure (PDB: 5CA1)^34^ and (B) based on (PDB: 6O5M).^39^

**Figure 6:**
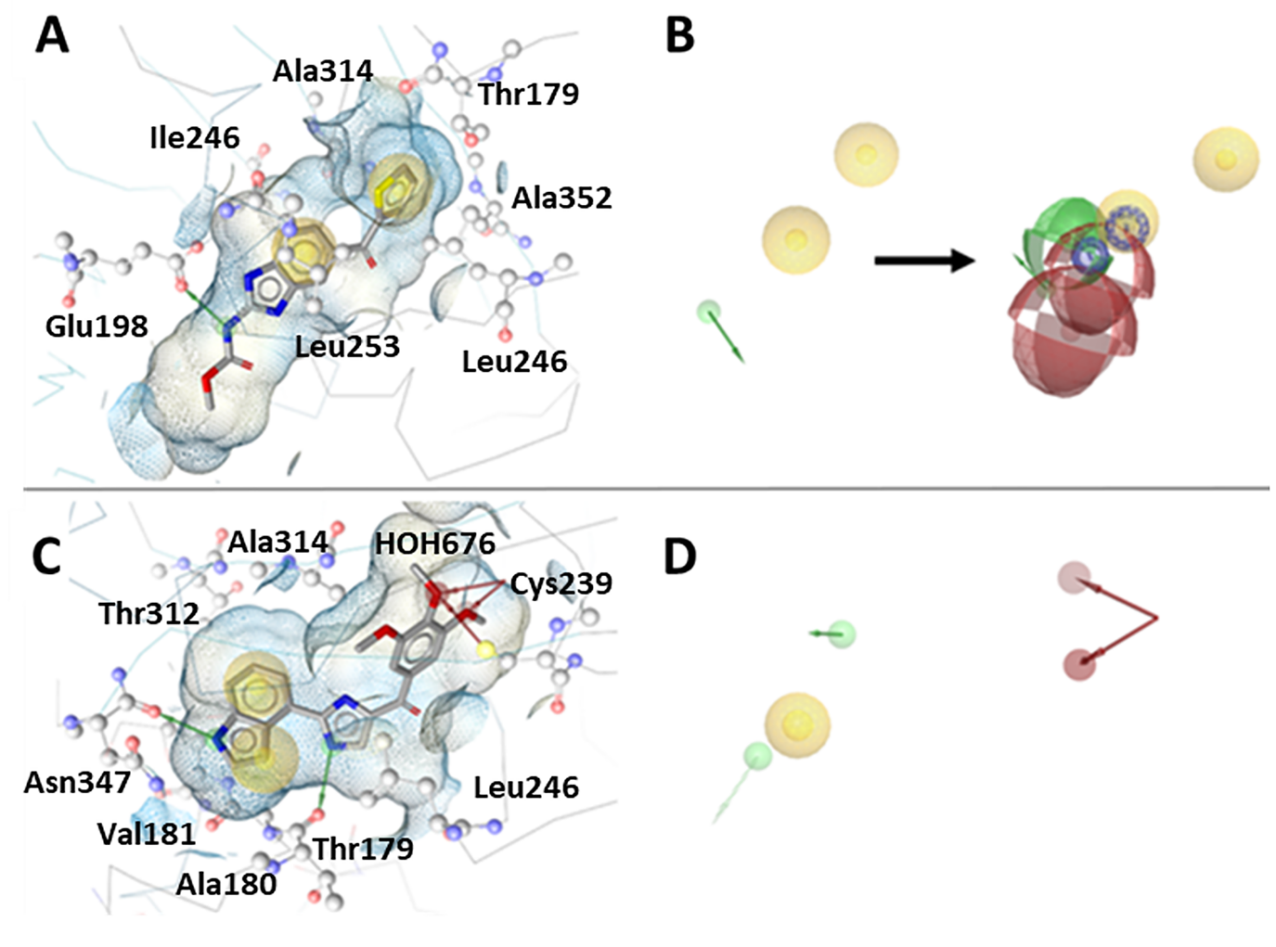
(A–D) SB pharmacophore models SB1 and SB2 generated using LS. The automatically generated models (A) and (C) were based on the β-tubulin X-ray crystal structures (PDB: 5CA1)^34^ and (PDB: 6O5M),^39^ respectively. Pharmacophore models were automatically generated with ligands **5** and **2** cocrystallized in the CBS. (B) SB1 model optimized features with additional LB features added. (D) SB2 model with optimized features. HC, yellow spheres; AI, purple circle; HBD, green arrows/spheres; HBA, red arrows/spheres.

The automatically generated SB models (SB1 and SB2) shown in Figure 5 were generated in the LS SB perspective and were based on coordinates of X-ray crystal structures (PDB: 5CA1(34) and PDB: 6O5M).^39^ The models initially generated showed distinct but unoptimized pharmacophore features. The initial SB1 model contained just three features, two HC and one HBD owed to its base ligand **5**. After optimization of these features, a further two HBD and two AI features were generated by aligning the common LB features of benzimidazole ligands from the actives set ligands. Both the SB and LB features were merged and the final SB1 model further optimized (Figure 6a, b). The automatically generated model of SB2 had two HC, one HBD, and two HBA features representing the important TMP moiety of **2** for CBS binding, including the water-mediated hydrogen bonding network with Cys239 and Gly235. These features were optimized accordingly for improved discriminatory power leading to the final SB2 model (Figure 6c, d).

**Figure 7:**
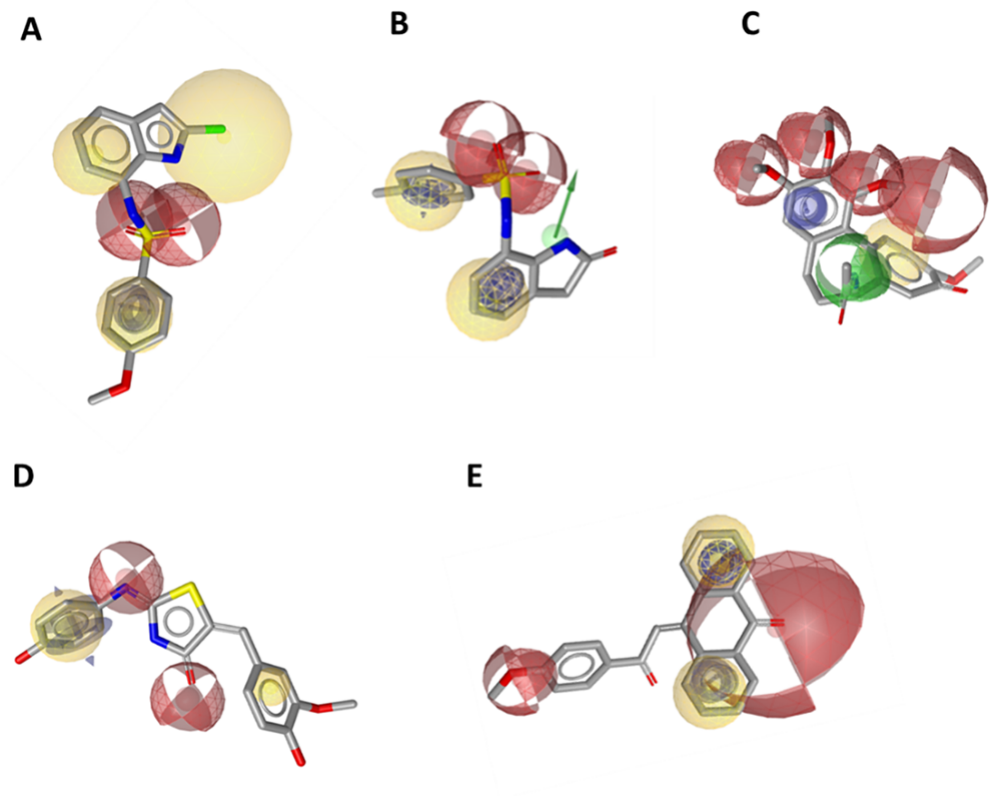
(A–E) LB pharmacophore models generated in LS were aligned with their respective ligands. HC, yellow spheres; AI, purple circle; HBD, green arrows/spheres; HBA, red arrows/spheres.

A total of 14 LB models were generated with LS and DS, respectively. Among the LS models, each was generated in the LB perspective by aligning a set of ligands from the actives data set using the merged or shared feature mode of the LB function. Each model was individually trained against the actives/decoys and optimized as described in SI (Section 2). The model LB3 (Figure 7A) depicted with ligand **AS55** was based on the pharmacophore features of sulfonamide-indole/sesquiterpenoid scaffolds. Model LB6 (Figure 7B) depicted with ligand **AS59** was based on the sulfonamide-indole scaffold. The model LB7 (Figure 7C) depicted with ligand **AS30** (**1a**, colchicine) was based on the colchicine/podophyllotoxin scaffolds. Model LB8 (Figure 7D) depicted with ligand **AS15** was based on thiazolidinedione/benzimidazole scaffolds. While LB10 (Figure 7E) depicted with ligand **AS80** was based on the anthracenone-methoxy phenyl scaffold. In the final models shown, all contained at least one HC, AI, and HBA feature, while LB6 and LB7 additionally possessed a HBD feature attributed to the indole and acetamide moieties.

**Figure 8:**
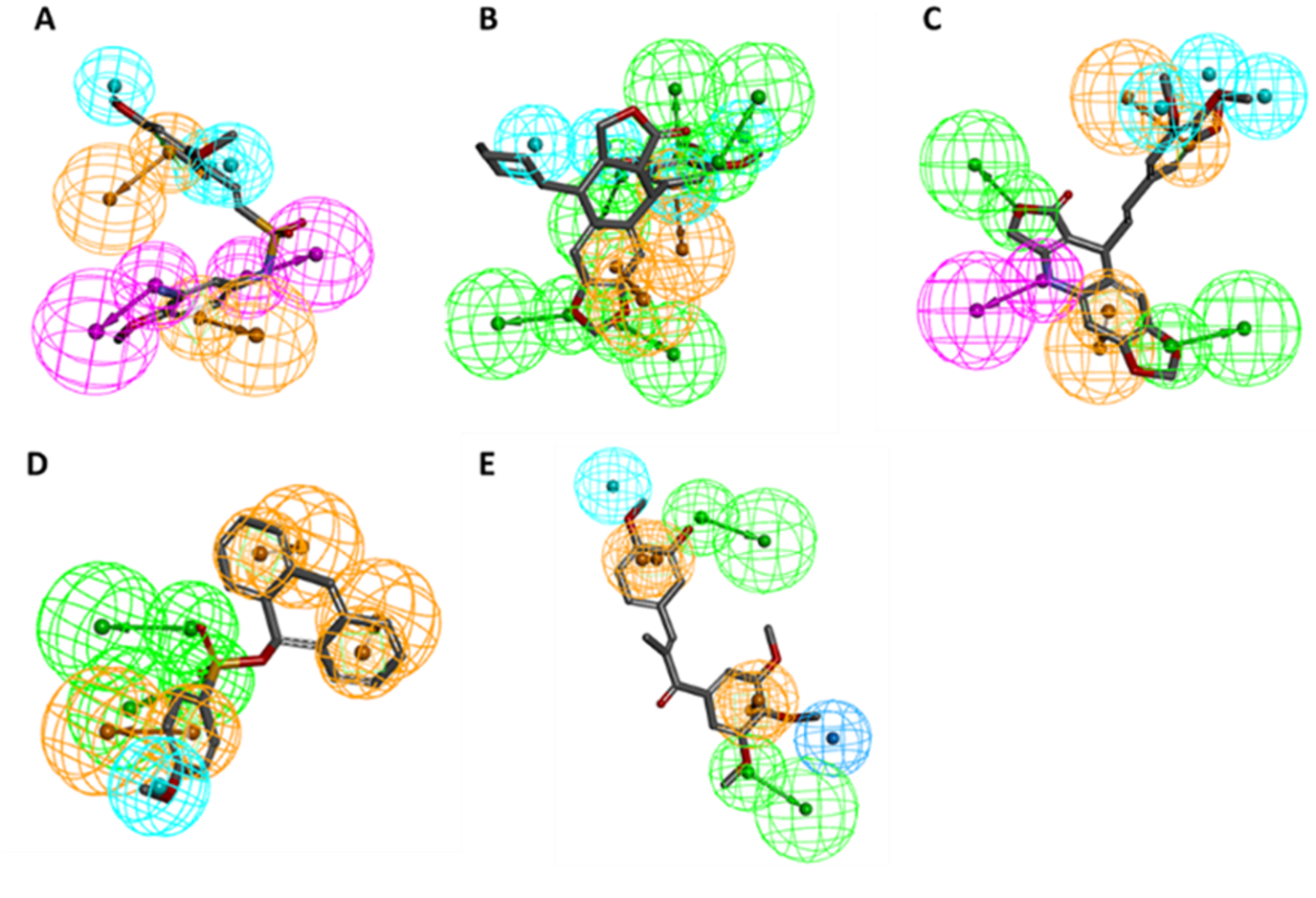
(A–E) LB pharmacophore models were generated in DS with their respective parent ligands. (A) Model DS2, (B) model DS5, (C) model DS8, (D) model DS9, and (E) model DS12. Pharmacophore features included HC (blue spheres), AI (brown spheres), HBA (green spheres), and HBD (purple spheres).

For all DS models, automatically generated feature tolerances were altered to optimize model performance. Xvols were added manually for steric refinement. Some DS models were excluded from the set (DS1, DS3, DS4, DS6, DS7, DS10, and DS11) due to poor model performance (i.e., EF < 4). The model DS2 was based on **AS64**, a diphenyl sulfonamide (Figure 8A). It contained three HC features, two ring AI features that are on the two phenyl rings, two HBD features, and 26 Xvols.

Model DS5 was based on **AS75**, a podophyllotoxin (Figure 8B). It contained four HC features, two AI features, five HBA features, and 34 Xvols (not shown in Figure 8B, see Figure S13, SI). The model DS8 was calculated for **AS77**, a modified podophyllotoxin scaffold with an amine functionality inserted in the central ring and an elongated linker to the trimethoxyphenylring (Figure 8C). The resulting pharmacophore consists of three HC features, two AI, two HBAs, one HBD feature, and 21 Xvols.

Model DS9 was derived from **AS79**, an anthracene sulfonate (Figure 8D). It consists of a HC feature, three AI features, two HBA features, and 60 Xvols.

The model DS12 was based on **AS29**, a chalcone (Figure 8E). The final model contains two HC, two AI, two HBA features, and 118 Xvols.

The theoretical evaluation of the 16 final pharmacophore models optimized against the training set is presented in Table 1. All the generated models rejected a large number of decoys and, thus, can be classified as highly specific (specificity value ranging from 0.96 to 0.98). However, due to the low number of TPs retrieved by each one, the individual models are also considered to have rather low sensitivity with values between 0.04 and 0.16. While all the models are determined to have high accuracy falling within 0.97–0.98, they were, however, distinct from one another regarding YoA and EF values. Model LB5 had the highest YoA and EF with values of 0.45 and 23.57, respectively, compared to the lowest scoring model LB10 with 0.08 and 4.17. Thus, considering all quantitative evaluation parameters, LB5 performed the best overall with the largest enrichment of active compounds over random selection in the theoretical validation. We aimed to cover more than 90% of the actives database with multiple highly selective models, rather than aiming for fewer and more general models. Despite the rather low sensitivity of each individual model, the combined pharmacophore models recognized a total of 89 TPs from the actives data set with an improved overall sensitivity of 0.94 and combined value of 6.11 for the enrichment factor.

**Table 1:** Theoretical Evaluation of LS and DS Generated Models^a^

Model	Actives	Decoy	TPs	FPs	TNs	FNs	Accuracy	Sensitivity	Specificity	YoA	EF
SB1	7	49	7	49	4852	88	0.97	0.07	0.99	0.13	6.57
SB2	6	69	6	69	4832	89	0.97	0.06	0.99	0.08	4.21
LB3	12	51	12	51	4850	83	0.97	0.13	0.99	0.19	10.02
LB4	8	15	8	15	4886	87	0.98	0.08	1.00	0.35	18.29
LB5	13	16	13	16	4885	82	0.98	0.14	1.00	0.45	23.57
LB6	5	24	5	24	4877	90	0.98	0.05	1.00	0.17	9.07
LB7	4	32	4	32	4869	91	0.98	0.04	0.99	0.11	5.84
LB8	3	18	3	18	4883	92	0.98	0.03	1.00	0.14	7.51
LB9	5	52	5	52	4849	90	0.97	0.05	0.99	0.09	4.61
LB10	5	58	5	58	4843	90	0.97	0.05	0.99	0.08	4.17
LB11	9	45	9	45	4856	86	0.97	0.09	0.99	0.17	8.76
DS2	10	49	10	49	4852	85	0.97	0.11	0.99	0.17	8.91
DS5	11	57	11	57	4844	84	0.97	0.12	0.99	0.16	8.51
DS8	10	74	10	74	4827	85	0.97	0.11	0.98	0.12	6.26
DS9	9	64	9	64	4837	86	0.97	0.09	0.99	0.12	6.48
DS12	15	86	15	86	4815	80	0.97	0.16	0.98	0.15	7.81
Combined	89	677	89	677	4224	6	0.86	0.94	0.86	0.12	6.11

a Each model was evaluated in relation to EF, YoA, and number of TPs.

Each model was continually optimized and evaluated to allow as many actives to be retrieved as possible and to exclude the decoy compounds. For the two final SB models, important features representing interactions between two different ligands with key residues at the CBS required for inhibition were retained. Thus, the two SB models displayed different interaction features from their respective crystal structures and met the theoretical evaluation criteria. However, the SB models matched just 13 actives out of 95 from the data set. Therefore, further LB models were generated based on inhibitor scaffolds from the active set to cover more of the active molecular space. After optimization, the additional nine LS models and five DS models retrieved further actives from the data set. A final optimization step was applied to each model, by enhancing the tolerance of or adding additional Xvols to reduce the number of inactive compounds that sterically clash with the protein to be matched with the pharmacophore. This resulted in an enriched final model set recognizing 89 out of 95 active compounds from the training set. This demonstrates that the application of two modeling software tools yielded a broader list of inhibitors, strengthening the model set’s ability to cover more of the active chemical space for virtual screening.

### 3.4 Virtual Screening

All models were screened against three databases. The resulting single hits (*n* = 1) of the combined virtual screening are presented in Table 2. Meanwhile the consensus hits that were found by means of more than one model (*n* = 2, *n*= 3) in the virtual screening can be seen in the SI (Section 3, Table S2). The model LB4 identified no virtual hits against all databases due to it being too selective (FPs = 15) and therefore could not be theoretically validated by the screening; thus, it was omitted from the workflow.

**Table 2:** Overview of Virtual Hits from *In Silico* Screening and Filtering of SPECS, SPECS (NP), and PhytChem (PC) Databases

Virtual screening (single hits)			
Model	SPECS (*n* = 208761)	SPECS (NP) (*n* = 736)	PhytoChem (PC) (*n* = 735)
SB1	64	0	0
SB2	121	0	1
LB3	2624	0	0
LB4	0	0	0
LB5	3050	3	8
LB6	242	0	0
LB7	25	0	1
LB8	210	0	0
LB9	50	0	1
LB10	216	1	0
LB11	567	1	4
DS2	399	1	14
DS5	63	16	0
DS8	210	9	11
DS9	50	7	4
DS12	2961	2	4
Total	5.20% (*n* = 10852)	5.43% (*n* = 40)	6.53% (*n* = 48)

After filtering of the virtual screening hit lists, as outlined in Section 2.5, a total of 47 compounds were purchased and selected for experimental validation in the tubulin polymerization inhibition assay. The final list of selected test compounds with their respective vendor IDs and matching pharmacophore models can be found in SI (Section 4, Table S3).

### 3.5 Tubulin Polymerization Inhibition

**Figure 9:**
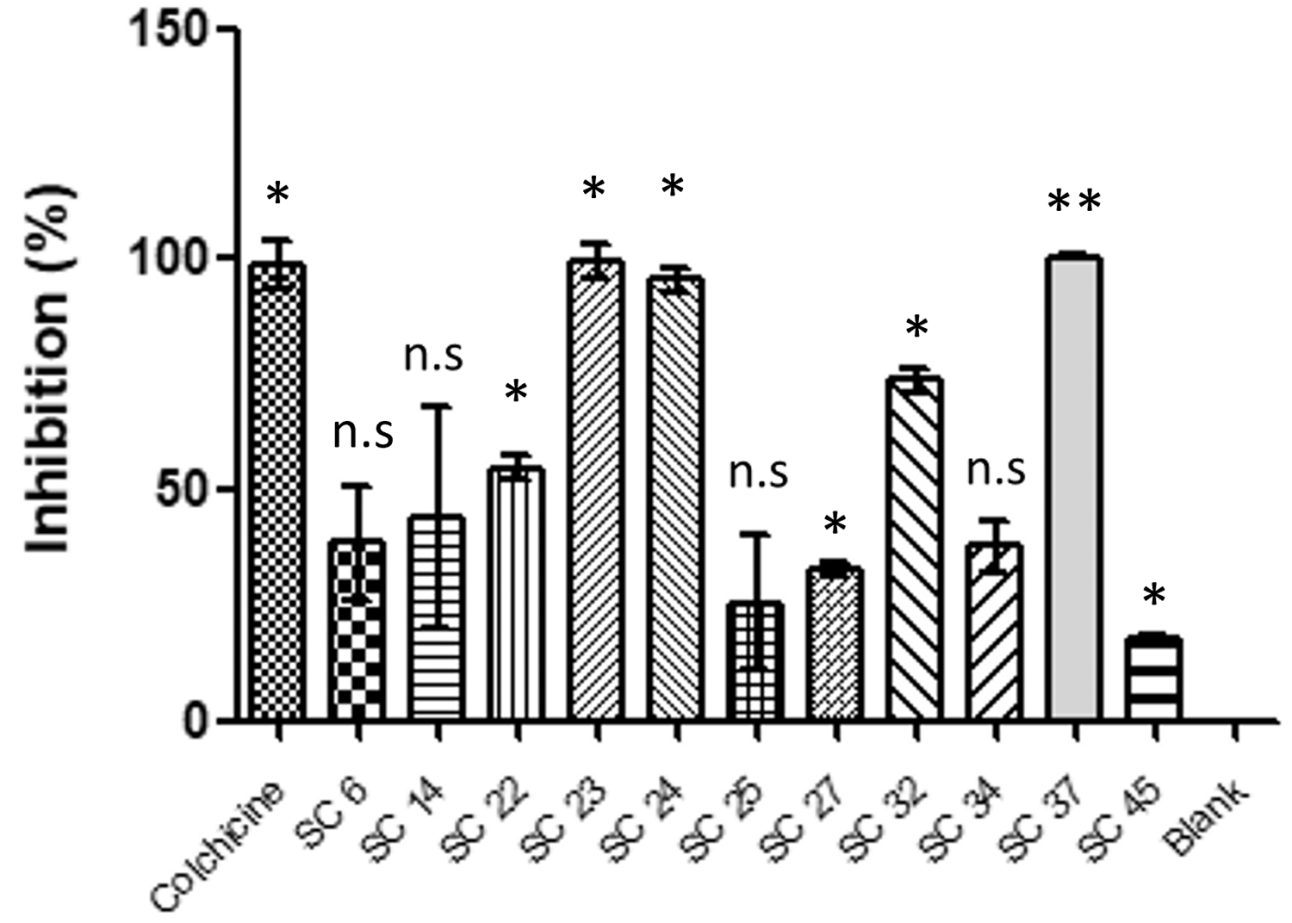
Tubulin polymerization inhibition screening results showing the active virtual hits at 30 μM (**SC6**, **SC22**, **SC23**, **SC25**, **SC27**, **SC32**, **SC34**, **SC37**, and colchicine **1a**), 20 μM (**SC24**), and 10 μM (**SC14**). Results expressed at time point 41.5 min as % inhibition of blank (mean ± SD, *n* = 2) and compared to control (**1a**) and blank. Statistical significance compared to blank analyzed by *t* test: *p < 0.05, **p < 0.01, n.s = not significant.

The 47 selected compounds (**SC1**–**SC46** and **Hit47**) were investigated in the previously described fluorescence-based cell-free tubulin polymerization assay.^59^ In an initial active/inactive discrimination screen, the compounds were tested at 30 μM (or when not fully soluble in DMSO/H_2_O at this concentration, at lower concentrations) to assess for polymerization inhibition. The highly active compounds (Figure 9 and Table 3) **SC23** and **SC37** displayed activities similar to the control **1a** with 99.3 ± 3.7% and 100.3 ± 0.4% inhibition, respectively, at 30 μM. Two compounds, i.e., **SC22** and **SC32**, showed moderate inhibition ranges (50%–75%) while four (i.e., **SC6**, **SC25**, **SC27**, and **SC34**) showed weak inhibition (25%–50%) at 30 μM concentration. Furthermore, the compound **SC24** tested at 20 μM showed high activity with 95.50 ± 2.52% tubulin inhibition. Due to their high activity at 30 μM, compounds **SC23** and **SC37** were also tested at 10 μM to consider potency evaluation experiments. Again, **SC23** showed high activity at 10 μM with 86.5 ± 2.7% inhibition and **SC37** at 10 μM had moderate activity with 71.3 ± 6.4% inhibition. Weak inhibition activity at 10 μM was determined for the other inhibitors **SC14**, **SC27**, and **SC45** (15%–50%). Six compounds (**SC7**, **SC9**, **SC10**, **SC12**, **SC18**, and **SC3**) were omitted from analysis due to solubility constraints or fluorescence signal interference.

**Table 3:** Summary of *In Vitro* Polymerization Inhibition of Compounds Considered Active^a^

Compound	Inhibition at 30 μM (%)	Inhibition at reduced concentration (%)	IC_50_ (μM), CI 95%
**SC6**	38.4 ± 12.6	n.d.	n.d.
**SC14**	insoluble	43.9 ± 23.9 (10 μM)	n.d.
**SC22**	54.7 ± 2.5	n.d.	n.d.
**SC23**	99.3 ± 3.7	86.5 ± 2.7 (10 μM)	2.9 (2.2–3.9)
**SC24**	insoluble	95.5 ± 2.5 (20 μM)	n.d.
**SC25**	25.5 ± 14.6	n.d.	n.d.
**SC27**	32.9 ± 1.7	35.9 ± 4.4 (10 μM)	n.d.
**SC32**	73.9 ± 0.2	n.d.	n.d.
**SC34**	37.8 ± 5.7	n.d.	n.d.
**SC37**	100.3 ± 0.4	71.3 ± 6.4 (10 μM)	5.8 (5.2–6.5)
**SC45**	Insoluble	17.7 ± 1.0 (10 μM)	n.d.
1a (Colchicine)	98.8 ± 5.2	83.2 ± 1.7 (10 μM)	2.3 (2.0–2.8)

a The assay measurements were performed at a concentration of 30, 20, or 10 μM. Polymerization inhibition (*n* = 2, mean ± SD) was normalized to percentage of the blank control at time point 41.5 min.

**Figure 10:**
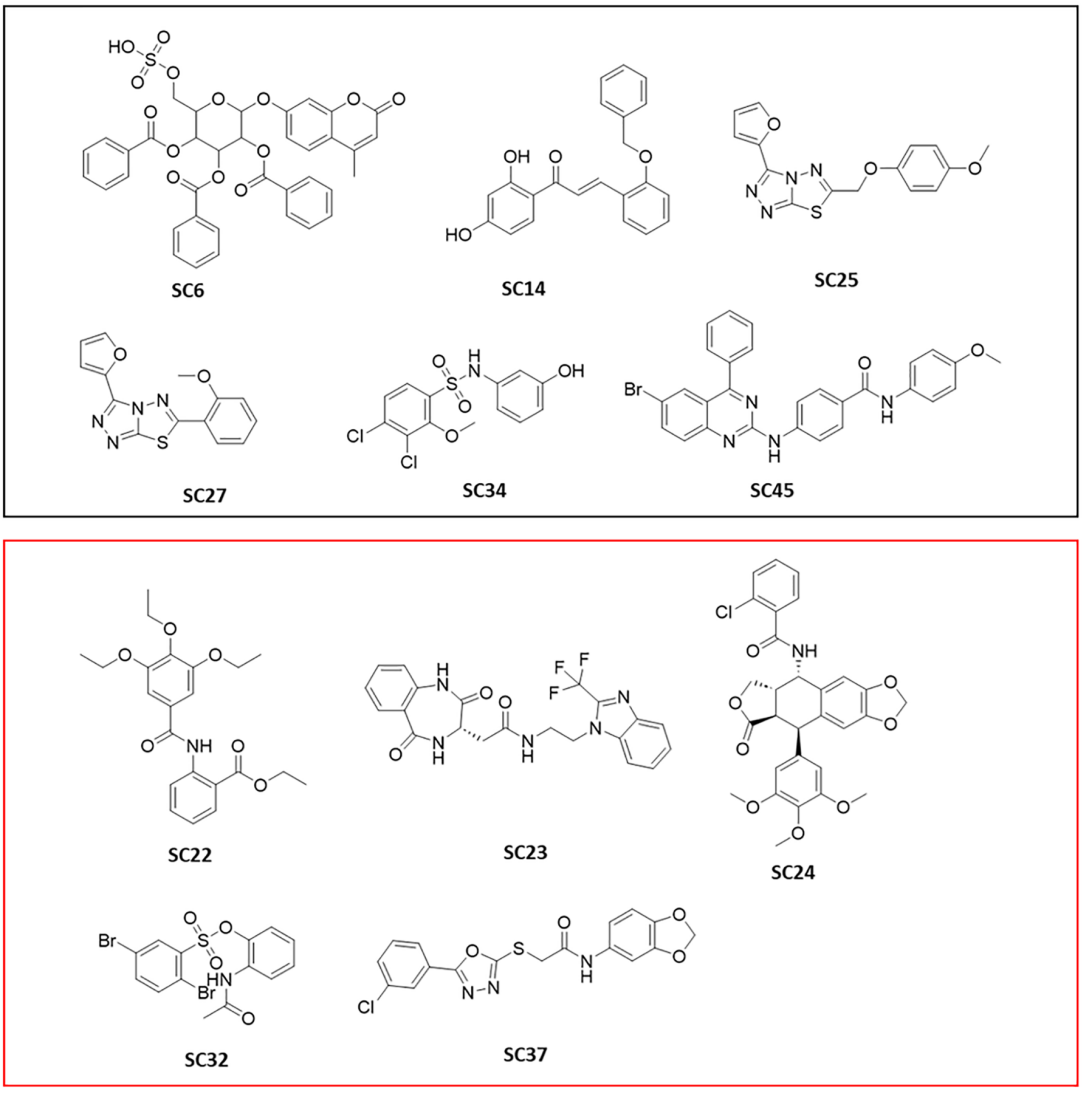
2D structures of the experimentally validated virtual hits identified in the screening assay. The purple box contains structures which had <50% inhibition, and the red box contains structures which had >50% inhibition in the tubulin polymerization assay.

Based on the preliminary screening, the most active inhibitors **SC23** and **SC37** were selected for IC_50_ experiments. Unfortunately, the other two promising inhibitors **SC32** and **SC24** had to be omitted from potency determination due to solubility constraints. All remaining selected test compounds were considered inactive at the tested concentrations. The assay results led to an experimental success rate of 26.8%. The overall results of the preliminary screen are presented in the SI (Section 5, Figure S18 and Table S4). The chemical structures of the active inhibitor compounds are shown in Figure 10.

**Figure 11:**
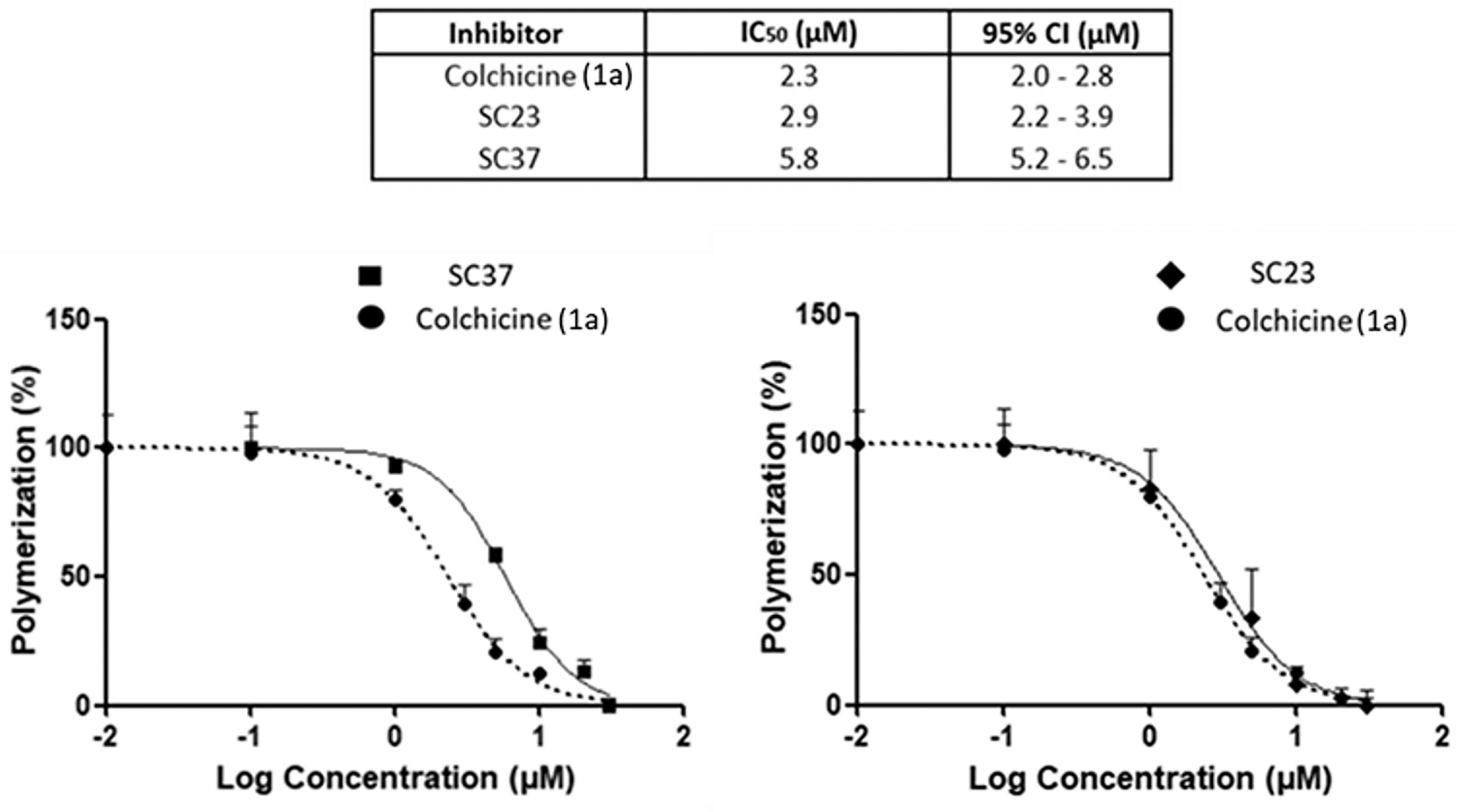
Concentration–response curves and respective IC_50_ values of the most active tubulin polymerization inhibitors **SC23**, **SC37**, and control **1a** (*n* = 3). Expressed as % polymerization with log (inhibitor) vs normalized response function, error bars (mean ± SD). Respective IC_50_ values and 95% confidence intervals for **SC23**, **SC37**, and **1a** are found in Table 3.

For the two virtual hits with the highest polymerization inhibition (compounds **SC23** and **SC37**), concentration–response measurements and potency values (IC_50_) were determined with results shown in Figure 11. When compared to the control **1a** (IC_50_ = 2.3 μM), **SC23** was found to be the most potent (IC_50_ = 2.9 μM). Meanwhile compound **SC37** was less potent (IC_50_ = 5.8 μM) but still in the range of **1a** within this assay.

### 3.6 Model Performance and Inhibitor-Model Alignment

Based on the experimental testing of 41 virtual hits selected from the 15 pharmacophore models, 13 models matched ligands with promising bioactivity. The prospective model performance is presented in Table 4. SB1 and SB2 each yielded one active compound in the bioassay: **SC45** and **SC23**, respectively. Four of LS LB models (i.e., LB3, LB7, LB8, and LB10) and four of the DS LB models (i.e., DS2, DS5, DS8, and DS12) also each matched one active. The best performing LS model was LB6, which had a hit rate of 60% and matched three virtual hits (i.e., **SC32**, **SC34**, and **SC37**) that were active in the bioassay. Meanwhile the best performing DS model was DS9, which matched four actives (**SC6, SC14, SC24, SC25**) and had an 80% hit rate. Furthermore, the combined SB and LB modeling approach with different software environments led to some consensus overlap between the different models. Within LS, the active **SC45** was matched with the models SB1/LB3. Furthermore, the active **SC34** matched with LB6/LB11/DS2 and the active **SC25** matched with LB10/DS9/DS12 were triple consensus hits. These findings demonstrate the utility of combining models from separate software programmes, leading to overlapping experimentally active compounds. Consensus actives were also distributed among the DS models. The highly active inhibitor **SC24** was matched with the three models DS5/DS8/DS9. This compound is a derivative of the well-known CBS ligand podophyllotoxin and thus distinctly highlights that the models are highly selective for such inhibitor scaffolds. Only inactives in the bioassay were found for the virtual hits selected from model LB9; thus, the model should be either reoptimized or disregarded for future screening programmes.

**Table 4:** Prospective Pharmacophore Model Performance Based on Bioactivity Results

Model (Found inhibitor)	Tested virtual hits	Active virtual hits	Number of active hits	Hit rate (%)
SB1	4	**SC45**	1	25
SB2	3	**SC23**	1	33.3
LB3	5	**SC45**	1	20
LB6	5	SC32, SC34, SC37	3	60
LB7	4	**SC22**	1	25
LB8	3	**SC27**	1	33.3
LB9	4	**0**	0	0
LB10	4	**SC25**	1	25
LB11	3	**SC34**	1	33.3
DS2	2	**SC34**	1	50
DS5	4	**SC24**	1	25
DS8	2	**SC24**	1	50
DS9	5	SC6, SC14, SC24, SC25	4	80
DS12	3	**SC25**	1	33.3

**Figure 12:**
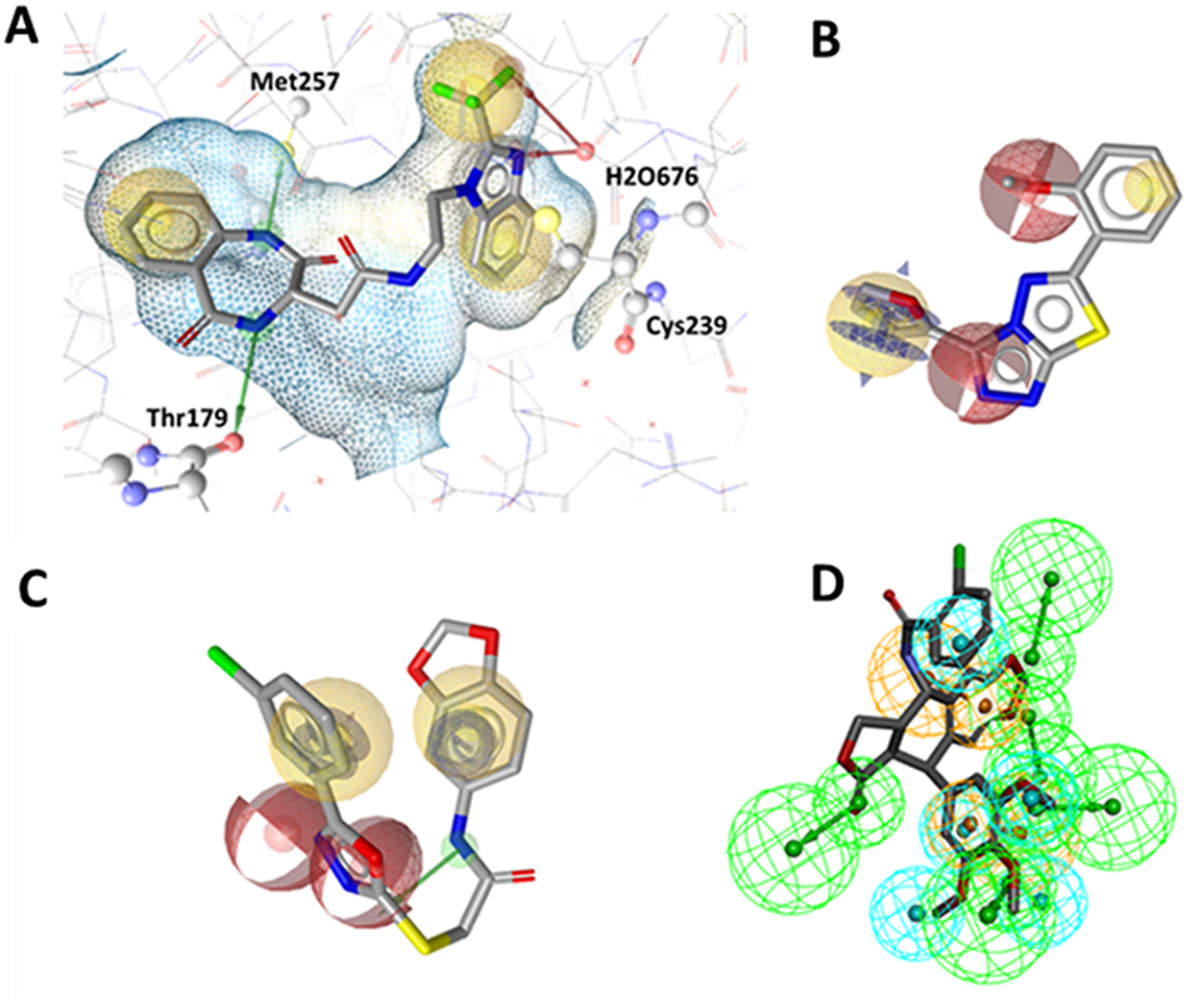
(A–D) Alignment of the most active tubulin inhibitors with their respective pharmacophore models generated in LS and DS. (A) **SC23** aligned to model SB2, (B) **SC27** aligned to model LB8, (C) **SC37** aligned to model LB6, and (D) **SC24** aligned to model DS5.

Using LS and DS software, the most active identified tubulin polymerization inhibitors were aligned to their respective pharmacophore models. The most potent inhibitor (compound **SC23**) was found by means of model SB2 (Figure 12A). It possessed two HBA features necessary for polar interaction with H_2_O676. This interaction resembles that of the complex’s parent cocrystal ligand **2**, but the interaction replaces the TMP methoxy groups with the nitrogen atom of the benzimidazole nucleus and a fluorine atom of the adjacent trifluoromethyl group. Furthermore, the inhibitor’s benzodiazepine ring shared the Thr179-mediated HBD interaction. Interestingly, this moiety also found a new HBD interaction with Met257 through the other nitrogen atom. Inhibitor **SC27** is a thiadiazol scaffold found by means of the LB8 model (Figure 12B). It possesses a HBA feature derived from its triazole ring. Furthermore, the aromatic and hydrogen bond features are owed to its furan ring, while the inhibitor retained one HBA feature of the methoxyphenyl group. The inhibitor **SC37** (Figure 12C) was found by means of model LB6, which was generated based on the benzenesulfonamide-indol **AS59**. The HBD originally due to the indole ring’s nitrogen atom is substituted with the nitrogen atom of the acetamide group linked to the benzodioxol ring. Further, the two HBA features originally owed to **AS59**’s sulfonamide oxygen atoms were substituted with **SC37**’s oxadiazol ring’s nitrogen and oxygen atoms. This demonstrates the pharmacophore’s ability to effectively scaffold hop and exchange functional groups while retaining bioactivity.

The active inhibitor **SC24** aligned to DS5 (Figure 12D) retained the TMP moiety HBA features that are crucial for interaction at the CBS. Found by multiple models, this virtual hit was identified as a triple consensus hit, thus demonstrating each model’s ability to select for known CBS inhibitor scaffolds.

### 3.7 Scaffold Novelty Evaluation

To assess scaffold novelty of the active tubulin inhibitors, their respective SMILES codes were submitted to a SciFinder literature search and to the online SwissTargetPrediction server (www.swisstargetprediction.ch, accessed 10/05/2023). No tubulin activity results were found for these compounds in the SciFinder search. The SwissTargetPrediction server estimates the probability for each query molecule to interact with known protein targets. The prediction is founded on 2D or 3D similarity of the molecule with a large library of known actives on 3000 protein targets linked to the ChEMBL assay database. The query for **SC24** offered 3D structurally similar molecules (*n* = 30) for the tubulin beta-1 chain target (CHEMBL1915) with high probability scores (0.75–0.90), which is due to the molecule’s well-known podophyllotoxin scaffold from which two of its parent models were built (DS5/DS8). Meanwhile **SC14** also matched for molecule 2D similarity (*n* = 11) with moderate probability scores (0.46–0.62) against the same target (CHEMBL1915). Interestingly, the query proposed no similarity results for the remaining actives (**SC6**, **SC22**, **SC23**, **SC25**, **SC27**, **SC32**, **SC34**, **SC37**, and **SC45**) against tubulin. This suggests some of these actives represent novel tubulin-inhibiting scaffolds and could be considered for further investigations as potential new lead candidates targeting the CBS of beta-tubulin. Furthermore, with low probability scores obtained for other targets, the actives identified might possess high selectivity for tubulin with limited off-target interactions. A tabular summary of the SwissTargetPrediction results for the actives is shown in SI (Table S5).

## Discussion and Conclusions

Out of the 16 pharmacophore models created in this study, 15 were used to retrieve virtual hits that were experimentally tested for their inhibitory activity against tubulin polymerization. In terms of individual model performance, the theoretical evaluation of the models revealed model LB5 to be the best performing, defined by the highest YoA (0.45) and EF (23.57) values, respectively. Model LB10 showed the worst performance considering YoA (0.08) and EF (4.17). Thus, the diverse model set displayed varied abilities for the enrichment of active compounds while rejecting decoys. Moreover, the final combined LS and DS model collection yielded 93.7% of the tubulin inhibitors from the actives data set, covering the diverse range of the active chemical space responsible for bioactivity. While both software solutions are based on the same principle, the feature definitions and screening algorithms vary, so that both programs have been shown to cover complementary parts of the active space.^44^ However, six tubulin inhibitors from the literature set were not identified by the model library (i.e., **AS1**, **AS14**, **AS24**, **AS44**, **AS47**, and **AS82**).^62−67^

Furthermore, model LB4 yielded no virtual hits during virtual screening and thus was excluded from the model set because it was considered too restrictive to retrieve hits for experimental validation.

In terms of experimental substantiation, among the 13 experimental bioassay validated models, SB2, a SB model representing the binding mode of ligand **2** in complex with amino acids at the CBS, showed good performance. It led to the highly active benzodiazepine/benzimidazole **SC23**, which was the most potent hit with an IC_50_ of 2.9 μM, making it the best performer of the two SB models. Alignment of this inhibitor at the CBS of crystal structure tubulin complex (PDB: 6O5M) revealed its pharmacophore interaction with H_2_O676 substitutes the HBA features of the TMP group of cocrystal ligand **2** reported by Wang et al.,^39^ with the nitrogen atom of **SC23**’s benzimidazole nucleus and a fluorine atom of its adjacent trifluoromethyl group. Furthermore, the new inhibitor’s benzodiazepine ring possessed a HBD interaction with Thr179 through its nitrogen atom, alike to the imidazol nitrogen atom of ligand **2** previously described.^39^ Interestingly, this moiety also found a new HBD interaction with Met257 mediated by the benzodiazepine ring’s other nitrogen atom, replacing the former indole HBD with Asn347, on which the feature was originally based on. Derivatives of both benzodiazepine and benzimidazole scaffolds are known independently to inhibit tubulin formation.^68,69^ However, this acetamide-bridged merged scaffold has no previous literature reports showing inhibitory potency against tubulin polymerization and would represent a novel merged scaffold for CBS tubulin inhibition. Furthermore, the scaffold novelty evaluation retrieved no similarity results against tubulin. Although some tubulin inhibitors from the actives set are known to be bioactive in the nanomolar range (SI, Table S1), the inhibition potency of **SC23** (2.9 μM) should be considered high, as it fell within range of the control **1a** and was more potent than the majority of micromolar range tubulin inhibitors from which the models were built. Some 1,4-benzodiazepine motifs coupled to the TMP moiety show nanomolar antiproliferative activity.^69,70^ Thus, it would be interesting to investigate how these merged structural features replacing the TMP with the benzimidazole nucleus might affect cell proliferation. This would support its consideration for potential application as a lead compound.

The best performing LB models were LS LB6 and DS DS9. The LB6 model had a hit rate of 60% finding three virtual hits (**SC32**, **SC34**, and **SC37**) that were inhibitors in the bioassay. Interestingly, actives **SC32** (inhibition = 73.9 ± 0.2%) and **SC34** (inhibition = 37.8 ± 5.7%) were sulfonamide and sulfonate derivatives resembling the parent scaffold of the pharmacophore model. However, the moderately active **SC32** had solubility constraints at 50 μM; therefore, it was not determined if its potency would fall within range of the parent ligands (IC_50_ = 1.1–2.9 μM).^71−73^ Despite the structural feature resemblance of these actives to their parent sulfonamide-indole ligands, the highly active and novel inhibitor **SC37** (inhibition = 100.3 ± 0.4%) was also yielded from the LB6 model. The structural difference of 1,3,4-oxadiazol-benzodioxol **SC37** effectively demonstrated the model’s ability to scaffold hop and exchange functional groups while retaining bioactivity of the pharmacophore. It possessed the acetamide-bridged benzodioxol and oxadiazol moieties wherein the HBD feature of the parent indole moiety was replaced with the acetamide group linked to the benzodioxol ring. In addition, the HBA features originally owed to **AS59**’s sulfonamide moiety were substituted with the oxadiazol ring in the pharmacophore of **SC37**. Furthermore, the active ligand demonstrated moderate potency (IC_50_ = 5.8 μM) within the range of the control **1a**. There were no previous literature reports with tubulin activity found for this hybrid compound. Despite SwissTarget prediction giving no similarity score for this active against tubulin, there are hybrids of the 1,3,4-oxadiazol and 1,3-benzodioxol moieties that have demonstrated both matrix metalloproteinase inhibition and *in vitro* anticancer activities.^74^ Furthermore, another study reported compounds with the oxadiazol moiety displaying tubulin inhibition (IC_50_ = 2.2–2.8 μM).^75^ Therefore, like **SC23**, this novel tubulin inhibition activity with an acetamide linkage of the moieties could also be considered further in CBS lead development.

Meanwhile the most successful DS model, DS9, led to four inhibitors (**SC6**, **SC14**, **SC24**, **SC25**), having an 80% experimental hit rate. Among these, the highly active **SC24** showed strong inhibition at 20 μM (95.5 ± 2.5%). This was unsurprising, as the compound is a podophyllotoxin derivative possessing the TMP moiety responsible for the antitubulin activity of this NP compound class. Furthermore, it showed high tubulin activity similarity to its analogous structures in the SwissTarget search. Due to solubility constraints, it was not possible to determine if this compound’s chlorobenzamide moiety is influential in terms of potency compared to other podophyllotoxin derivatives.

However, this activity was also found by means of two other DS models (DS5/DS9). Thus, as a triple consensus hit, it effectively advocates for the utility of pharmacophore modeling for identifying previously known CBS tubulin-inhibiting scaffolds. All remaining actives found by means of the models displayed weak to moderate tubulin inhibition.

**Figure 13:**
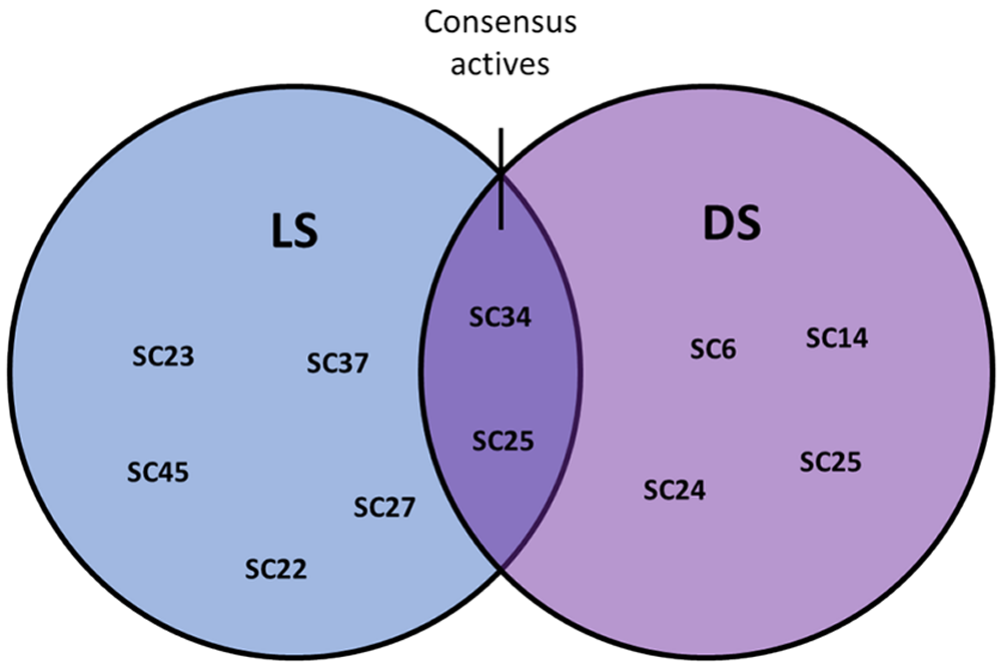
Venn diagram visually displaying the bioassay active virtual hits matched in each modeling program (LS, blue), (DS, Purple). The dark overlapping region shows the bioassay consensus actives matched in both programs.

Within LS, the weakly active **SC45** was matched with the models SB1/LB3. Furthermore, the weak active **SC34** matched with LB6/LB11/DS2 and the weak active **SC25** matched with LB10/DS9/DS12 were triple consensus hits. Despite being weakly active, these hits convey the benefit of a hyphenated LB and SB modeling approach alongside combining different software for discovering active tubulin inhibitors (Figure 13). The active inhibitors **SC27** (LB8) and **SC25** (LB10/DS9/DS12) are triazolo-thiadiazol derivatives with the known tubulin-inhibiting thiadiazol scaffold. Although there was no tubulin activity similarity for these derivatives from the SwissTarget search, they were both previously screened in a HeLa cell-based HCS assay for microtubule stabilizers and were reported to be inactive at 10 μM in the bioassay report.^76^ Considering these derivatives were not cytotoxic for the endpoint measurement of cellular microtubule stabilization, our results suggest that alternative derivatives of these tubulin polymerization inhibitors could be considered further for tubulin destabilization and antiproliferative screening investigations wherein a CBS tubulin destabilization mechanism of action is exerted. These actives differ only by the 2-methoxyphenyl and 4-methoxyphenoxy groups linked to the triazol ring and presented comparable tubulin inhibition capacities in the assay.

The models indicated that they possess a HBA feature derived from the triazole ring. Furthermore, the aromatic and hydrogen bond features belonging to the furan ring are present; meanwhile, these inhibitors retained one HBA feature of their respective methoxyphenyl and methoxyphenoxy groups. Interestingly, the structurally analogous triazolo-thiadiazol **SC26** (LB3/LB5) was an inactive test compound. It has a phenoxypropyl group instead of a methoyphenyl/methoxyphenoxy group, emphasizing the importance of these additional moieties for establishing a bioactive pharmacophore among this structural class.

To conclude, a pharmacophore-based virtual screening workflow was designed and subsequently led to the identification of 11 novel tubulin polymerization inhibitors with a combined experimental hit rate of 26.8%. The 13 optimized and experimentally validated pharmacophore models can be considered as efficient tools for the prioritization of compounds for future bioassay screening. The two most potent tubulin inhibitors identified demonstrate the effectiveness of acetamide linkages of tubulin-inhibiting warheads, and these merged scaffolds could potentially contribute to further development of lead structures targeting the CBS of tubulin for cancer treatment. Next, future investigations will be considered to assess the pharmacological potential of the most active compounds against cancer cell lines or parasitic embryo development. Furthermore, the collated CBS model library will be cross-functionally utilized for virtual screening of additional natural product databases to prospect for novel CBS tubulin inhibitors for numerous therapeutic areas (inflammation, anti-infectives, cancer).

## Data Availability

All data sets used within this work are available as sd files as supporting files to this publication. For the generation and optimization of pharmacophore models as well as the prospective screening of the SPECS database, LigandScout version 4.08 was used. A trial version of this commercial software valid for one month can be acquired from inte:ligand using the following link: http://www.inteligand.com/cgi-bin/ligandscout4/register.pl. BIOVIA’s Discovery Studio and Pipeline Pilot are a commercial software that can be acquired online using the 3DS website https://www.3ds.com/products-services/biovia/products/data-science/pipeline-pilot/ and choosing the contact us option. The database of readily commercially available compound SPECS can be downloaded upon creating an account at www.specs.net website in the download databases section or under https://www.specs.net/index.php?view=databases&page=download. The database of compounds available at 10 mg was screened.

## Supporting Information Available

1. List of ligands active against tubulin used to generate the actives data set for pharmacophore model training. 2. Description and optimization of pharmacophore models. 3. Results of virtual screening of pharmacophore models with consensus hits. 4. Test compound selection of hits obtained from virtual screening of the databases. 5. Bioactivity results of selected test compounds from the polymerization inhibition assay and similarity prediction. 6. Active compound characterization provided by Specs 7. LC-MS and ^1^H NMR data provided by Specs. (PDF)

## Abbreviations

AI: aromatic interaction
AUC: area under the curve
CBS: colchicine-binding site
CBSI: colchicine-binding site inhibitor
DS: Discovery Studio
EF: enrichment factor
GTP: Guanosine-5′-triphosphate
HBA: hydrogen bond acceptor
HBD: hydrogen bond donor
HC: hydrophobic contact
LS: LigandScout
LB: ligand-based
MDR: multidrug resistance
P-GP: P-glycoprotein
SB: structure-based
SI: Supporting Information
TMP: trimethoxyphenyl
YoA: yield of actives
