# Supplementary material for: Identification of Novel β-Tubulin Inhibitors Using a Combined In Silico/In Vitro Approach
Source: J Chem Inf Model. 2023 Sep 29;63(20):6396–411. doi: 10.1021/acs.jcim.3c00939 (PMC10598795; doi:10.1021/acs.jcim.3c00939)
Supplement: Supplementary file 1 — ci3c00939_si_001.pdf [file ci3c00939_si_001.pdf]

Identification of Novel β-Tubulin Inhibitors Using a Combined *In Silico*/*In Vitro* Approach

*Mark James Horgan[1], Lukas Zell[2], Bianka Siewert[1], Hermann Stuppner[1], Daniela Schuster[2] and *Veronika Temml[2]*

[1]Institute of Pharmacy/Pharmacognosy, Center for Chemistry and Biomedicine, University of Innsbruck, Innrain 80-82, 6020 Innsbruck, Austria. [2]Institute of Pharmacy, Department of Pharmaceutical and Medicinal Chemistry, Paracelsus Medical University Salzburg, Strubergasse 21, 5020 Salzburg, Austria.

*E-mail: veronika.temml@pmu.ac.at

Supporting Information

1. Actives dataset generation

Table S1. List of ligands active against tubulin used to generate the actives dataset for pharmacophore model training.

ID	Name	Tubulin activity	SMILES	Reference
**AS1**	Thiabendazole	81.6 ± 1% inhibition at 20 µM	C1=CC=C2C(=C1)NC(=N2)C3=CSC=N3	1
**AS2**	Albendazole	IC_{50} = 6.9 µM	CCCSC1=CC2=C(C=C1)N=C(N2)NC(=O)OC	2

**AS3**	Fenbendazole	IC_{50} = 5.4 µM	COC(=O)Nc1nc2ccc(Sc3ccccc3)cc2[nH]1	[2]
**AS7**	Mebendazole	IC_{50} = 6.1 µM	COC(=O)NC1=NC2=C(N1)C=C(C=C2)C(=O)C3=CC=CC=C3	[2]
**AS8**	Flubendazol	IC_{50} = 3.5 µM	COC(=O)Nc1nc2cc(C(=O)c3ccc(F)cc3)ccc2[nH]1	[2]
**AS9**	Parbendazol	IC_{50} = 3.1 µM	CCCCc1ccc2[nH]c(NC(=O)OC)nc2c1	[2]
**AS10**	Oxfendazole	IB_{50} = 1.3 µM (Mebendazole inhibitory binding)	COC(=O)NC1=NC2=C(N1)C=C(C=C2)S(=O)C3=CC=CC=C3	[3]
**AS11**	Oxibendazole	IC_{50} = 2.4 µM	CCCOc1ccc2[nH]c(NC(=O)OC)nc2c1	[2]
**AS5**	Nocodazole	IC_{50} = 1.82 ± 0.06µM	COC(=O)NC1=NC2=C(N1)C=C(C=C2)C(=O)C3=CC=CS3	[4]
**AS12**	CHEMBL459773	IC_{50} = 4.23 µM	COC1=C(OC)C(OC)=CC(C(C2=CSC(C3=CC=CC=C3)=N2)=O)=C1	[5]
**AS13**	CHEMBL3426932	IC_{50} = 2.36 µM	COC1=C(OC)C(OC)=CC(C2=C(C3=CC=C(OCC)C=C3)N=C(N)S2)=C1	[6]
**AS14**	CHEMBL273081	IC_{50} = 16.75 µM	ClC1=C(/N=C2S/C(C(N/2)=O)=C/C3=CC=C(N(C)C)C=C3)C=CC=C1	[7]
**AS15**	24045-22-5	IC_{50} = 4.0 ± 0.6 µM	O=C(N/1)/C(SC1=N\C2=CC=C(O)C=C2)=C\C3=CC(OC)=C(O)C=C3	[8]
**AS16**	1596344-07-8	IC_{50} = 5.6 ± 0.3 µM	O=C(N/1)/C(SC1=N\C2=CC=C(OC3=CC=CC=C3)C=C2)=C\C4=C(O)C=CC=C4	[8]
**AS17**	463979-31-9	IC_{50} = 6.28 µM	O=C(N/1)/C(SC1=N\C2=CC=C(O)C=C2)=C\C3=CC(OC)=CC=C3	[9]
**AS4**	356542-94-4	IC_{50} = 1.5 µM	O=C(C1=CC(OC)=C(O)C=C1)/C=C/C2=C(OC)C=C(OC)C=C2OC	[10,11]
**AS18**	CHEMBL1163178	IC_{50} = 6.8 ± 0.6 µM	O=C(C1=CC=C(Cl)C=C1)CC(SC(N(CC)CC)=S)C2=CC=CC=C2	[12]
**AS19**	CHEMBL220818	IC_{50} = 2.2 µM	O=C(C1=CC(OC)=C(OC)C(OC)=C1)/C=C/C2=CC=C(C=CC=C3)C3=C2	[13]
**AS20**	CHEMBL285901	IC_{50} = 2.8 µM	O=C(C1=CC(OC)=C(OC)C(OC)=C1)/C=C/C2=CC=C(N(C)C)C=C2	[13]
**AS21**	1585234-76-9	IC_{50} = 1.6 ± 0.32 µM	COC1=C(C(OC)=CC(OC)=C1)/C=C/C(C2=CC(N)=CC=C2)=N\O	[14]
**AS22**	CHEMBL3393090	IC_{50} = 1.3 ± 0.0 µM	OC1=CC(/C=C/C(C2=CC=CC=C2C3=CC=C(F)C=C3)=O)=CC=C1OC	[15]
**AS23**	CHEMBL3393091	IC_{50} = 3.4 ± 0.2 µM	FC(C=C1)=CC=C1C2=CC=CC=C2C(/C=C/C3=CC=C(OC)C(B(O)O)=C3)=O	[15]
**AS24**	CHEMBL3393092	IC_{50} = 3.6 ± 0.0 µM	FC(C=C1)=CC=C1C2=CC=CC=C2C(/C=C/C3=CC=C(N(C)C)C=C3)=O	[15]
**AS25**	CHEMBL3393099	IC_{50} = 0.49 ± 0.27 µM	FC1=CC=CC(C2=CC=CC=C2C(/C=C/C3=CC=C(C(O)=C3)OC)=O)=C1	[15]
**AS26**	CHEMBL3818930	IC_{50} = 1.99 ± 0.03 µM	O=C(/C(CC1=C2OC)=C/C3=C4C=CNC4=CC=C3)C1=CC(OC)=C2OC	[16]
**AS27**	CHEMBL3818850	IC_{50} = 2.68 ± 0.15 µM	O=C(C1=CC(OC)=C(OC)C(OC)=C1)/C(C)=C/C2=CNC3=C2C=CC(OC)=C3	[17]

**AS28**	CHEMBL595808	IC_{50} = 12 µM	COC1=C(OC)C(OC)=CC(C(/C=C/C2=C(OC)C=C(OC)C=C2OC)=O)=C1	18
**AS29**	CHEMBL9859	IC_{50} = 0.46 µM	COC1=C(OC)C(OC)=CC(C(/C(C)=C/C2=CC=C(OC)C(O)=C2)=O)=C1	19
**AS30**	CHEMBL107	IC_{50} = 6.7 ± 0.05 µM	CC(=O)N[C@H]1CCC2=CC(=C(C(=C2C3=CC=C(C(=O)C=C13)OC)OC)OC)OC	20
**AS31**	CHEMBL208189	IC_{50} = 8.3 µM	CC(=O)CN[C@H]1CCC2=CC(=C(C(=C2C3=CC=C(C(=O)C=C13)OC)OC)OC)OC	21
**AS32**	CHEMBL206877	IC_{50} = 4.6± 0.17 µM	COC1=CC=C2C(=CC1=O)[C@H](CCC3=CC(=C(C(=C32)OC)OC)OC)NC4=C(C=C(C=C4)[N+](=O)[O-])[N+](=O)[O-]	21
**AS33**	CHEMBL2180999	IC_{50} = 1.62 µM	COC1=CC=C2C(=CC1=O)[C@H](CCC3=CC(=C(C(=C32)OC)OC)OC)NCC4=C(C(=CC=C4)F)F	22
**AS34**	CHEMBL4553296	IC_{50} = 0.05 µM	O(C)C1=C2C=3C([C@@H](NC(C)=O)CCC2=CC(OC)=C1OC)=CC(=O)C(NC4=CC(C(F)(F)F)=C(Cl)C=C4)=CC3	23
**AS35**	CHEMBL1946973	IC_{50} = 1.1 ± 0.1 µM	COC1=C(OC)C(OC)=CC(N2C(C3=CC=C(OCC)C=C3)=NN=N2)=C1	24
**AS36**	CHEMBL1823147	IC_{50} = 3.0 ± 0.6 µM	COC1=C(OC)C(OC)=CC(C(C2=C(C)OC3=C(O)C(OC)=CC=C23)=O)=C1	25
**AS37**	CHEMBL3580699	IC_{50} = 14 ± 2.2 µM	COC1=C(OC)C(OC)=CC(C2=C(NC3=CC=C(OC)C=C3)N(C=CC=C4)C4=N2)=C1	26
**AS38**	7-Methoxy-4-(3,4,5-trimethoxyphenyl)quinolin-2(1H)-one	IC_{50} = 2.56 ± 0.15 µM	COC1=C(OC)C(OC)=CC(C(C2=CC=C(CC)C=C2N3)=CC3=O)=C1	27
**AS39**	CHEMBL221765	IC_{50} = 2.0 µM	COC1=CC(SC2=C(C(OC)=O)NC3=CC=C(OC)C=C23)=CC(OC)=C1OC	28
**AS40**	CHEMBL380871	IC_{50} = 4.5 ± 0.1 µM	COC1=CC(SC2=C(C(OC)=O)NC3=CC=C([N+]([O-])=O)C=C23)=CC(OC)=C1OC	29
**AS41**	1179587-98-4	IC50 = 1.3 ± 0.1 µM	COC1=CC(SC2=C(C(OC)=O)NC3=CC=C(Br)C=C23)=CC(OC)=C1OC	30
**AS42**	1179588-09-0	IC_{50} = 0.67 ± 0.02 µM	COC1=CC(C(C2=C(C(OC)=O)NC3=CC=C(OC)C=C23)=O)=CC(OC)=C1OC	30
**AS43**	1839121-23-1	IC_{50} = 8.3 µM	O=C(C(NC1=CC=C(OC)N=C1)=O)C2=CN(CC3OCCC3)C4=C2C=CC=C4	31
**AS44**	CHEMBL3747333	IC_{50} = 6.6 µM	O=C(C(NC1=CC=C(OC(C)C)N=C1)=O)C2=CN(CCOC)C3=C2C=CC=C3	31
**AS45**	1582297-75-3	IC_{50} = 1.65 µM	CC1=C([Se](C2=CC(OC)=C(OC)C(OC)=C2)=O)C3=C(N1)C=CC=C3	32
**AS46**	CHEMBL4092940	IC_{50} = 11.2 ± 1.13 µM	ClC1=CC(NC(C(C2=CC=CS2)=O)=C3NC4=C([N+]([O-])=O)C=NC(N5CCN(C)CC5)=N4)=C3C=C1	33
**AS47**	CHEMBL1940254	IC_{50} = 1.1 ± 0.5 µM	COC1=CC(SC2=C(C3=CC=CN3)NC4=CC=CC=C24)=CC(OC)=C1OC	34

**AS48**	CHEMBL1940258	IC_{50} = 0.74 ± 0.05 µM	COC1=CC(SC2=C(C3=CC=CS3)NC4=CC=CC=C24)=CC(OC)=C1OC	[34]
**AS49**	CHEMBL2313790	IC_{50} = 1.3 ± 0.06 µM	COC1=CC(SC2=C(N3C=CN=C3)NC4=CC=CC=C24)=CC(OC)=C1OC	[35]
**AS50**	CHEMBL3597212	IC_{50} = 1.1 ± 0.1 µM	COC1=CC(SC2=C(C3=CC=CC=C3)NC4=CC(OC)=CC=C24)=CC(OC)=C1OC	[36]
**AS51**	CHEMBL3597223	IC_{50} = 1.2 ± 0.1 µM	COC1=CC(=CC(=C1OC)OC)SC2=C(NC3=C2C=CC(=C3Cl)Cl)C4=CC=CC=C4	[36]
**AS52**	CHEMBL89341	IC_{50} = 1.1 ± 0.4 µM	COC1=CC(C(C2=C(C3=CC(O)=C(OC)C=C3)NC4=CC(OC)=CC=C24)=O)=CC(OC)=C1OC	[37]
**AS53**	CHEMBL2442345	IC_{50} = 1.1 ± 0.4 µM	COC1=CC(C(C2=C(C3=CC(O)=C(OC)C=C3)NC4=C(OC)C(OC)=CC=C24)=O)=CC(OC)=C1OC	[37]
**AS54**	CHEMBL20684	IC_{50} = 2.2 µM	COC1=CC=C(S(NC2=CC=CN=C2NC3=CC=C(O)C=C3)(=O)=O)C=C1	[38]
**AS55**	CHEMBL281995	IC_{50} = 2.1 µM	COC1=CC=C(S(NC2=C3C(C=C(Cl)N3)=CC=C2)(=O)=O)C=C1	[38]
**AS56**	CHEMBL20289	IC_{50} = 9.5 µM	COC1=CC=C(S(NC2=C3C(C(Cl)=CN3)=CC=C2)(=O)=O)C=C1	[38]
**AS57**	CHEMBL20296	IC_{50} = 88.5 µM	ClC1=CC=C(S(NC2=C3C(C=C(Cl)N3)=CC=C2)(=O)=O)C=C1	[39]
**AS58**	CHEMBL20800	IC_{50} = 2.9 µM	CC1=CC=C(C=C1)S(=O)(=O)NC2=CC=CC3=C2NC(=C3)O	[39]
**AS59**	SCHEMBL3072090	IC_{50} = 2.5 µM	CC1=CC=C(S(NC2=C3C(CC(N3)=O)=CC=C2)(=O)=O)C=C1	[40]
**AS60**	CHEMBL216379	IC_{50} = 1.1 ± 0.1 µM	O=C(NC1=C2C(CCN2S(C3=CC=C(OC)C=C3)(=O)=O)=CC=C1)C4=CC=NC=C4	[41]
**AS61**	CHEMBL216327	IC_{50} = 1.2 ± 0.1 µM	O=C(NC1=C2C(CCN2S(C3=CC=C(OC)C=C3)(=O)=O)=CC=C1)C4=CC=CO4	[41]
**AS62**	CHEMBL230296	IC_{50} = 0.6 µM	COC1=NC(OC)=C(NS(C2=CC3=C(N(C)C4=C3C=CC=C4)C=C2)(=O)=O)C=C1	[42]
**AS63**	CHEMBL79280 (Betabulin)	IC_{50} = 2 µM	COC1=C(F)C=C(NS(C2=C(F)C(F)=C(F)C(F)=C2F)(=O)=O)C=C1	[43]
**AS64**	CHEMBL2397992	IC_{50} = 3.68 µM	COC1=CC(OC)=CC(OC)=C1/C=C/S(NC2=CC=C(OC)C(N)=C2)(=O)=O	[44]
**AS65**	CHEMBL141689	IC_{50} = 3.6 µM	COC1=CC=C(CNC2=NC(NCC3=CC=C(OC)C=C3)=C4C(N(C(C)C)C=N4)=N2)C=C1	[45]
**AS66**	CHEMBL2001473	Tubulin polymerization MIC = 20 µM	COC1=CC=C(CNC2=NC(NCC3=CC=C(OC)C=C3)=NC(NC4CCCCC4)=N2)C=C1	[46]
**AS67**	CHEMBL1968484	Tubulin polymerization MIC = 10 µM	COC1=CC=C(CNC2=NC(NCC3=CC=C(OC)C=C3)=NC(NC(C)C)=N2)C=C1	[46]
**AS68**	CHEMBL1976996	Tubulin polymerization MIC = 5 µM	COC1=C(OC)C=C(CNC2=NC(NCC3=CC=C(OC)C=C3)=NC(NC4CCCCC4)=N2)C=C1	[46]

**AS69**	CHEMBL141477	IC_{50} = 2 µM	COC1=CC=C(CNC2=NC(NCC3=CC=C(OC)C=C3)=C4C(N(C5CCCCC5)C=N4)=N2)C=C1	47
**AS70**	SCHEMBL2905972	IC_{50} = 16 ± 2 µM	COC1=CC=C(CNC2=NC(NCC3=CC=C(OC)C=C3)=C4C(C(C(C)C)=NN4)=N2)C=C1	48
**AS71**	CHEMBL513813	IC_{50} = 3.4 µM	COC1=CC=C(CNC2=NC3=CC=NN3C(NCC4=CC=C(OC)C=C4)=N2)C=C1	45
**AS72**	CHEMBL466716	IC_{50} = 3.6 µM	COC1=CC=C(CNC2=NC3=C(C(C)C)C=NN3C(NCC4=CC=C(OC)C=C4)=N2)C=C1	45
**AS6**	CHEMBL61	IC_{50} = 1.3 ± 0.06 µM	COC(C=C([C@H]1C2[C@H](COC2=O)[C@@H](O)C3=CC4=C(OCO4)C=C13)C=C5OC)=C5OC	49
**AS73**	CHEMBL2165487	IC_{50} = 7.7 ± 1.1 µM	COc1cc([C@@H]2c3cc4c(cc3[C@](O)(C3(O)CCCCC3)[C@H]3COC(=O)[C@H]23)OCO4)cc(OC)c1OC	50
**AS74**	CHEMBL2165463	21.1 % inhibition at 1 µM	COc1cc([C@@H]2c3cc4c(cc3[C@@](O)(C3(O)CCOCC3)[C@H]3COC(=O)[C@@H]23)OCO4)cc(OC)c1OC	50
**AS75**	CHEMBL2165468	84.5% inhibition at 1 µM (IC50 = 0.45 ± 0.05 µM)	O=C1[C@@]2([C@@H](C=3C([C@H]([C@@H](C)C)[C@]2(CO1)[H])=CC4=C(C3)OCO4)C5=CC(OC)=C(OC)C(OC)=C5)[H]	50
**AS76**	266685-31-8	IC_{50} = 0.17 µM	COC(C=C(C1C2=C(COC2=O)NC3=CC4=C(OCO4)C=C13)C=C5OC)=C5OC	51
**AS77**	1260403-30-2	IC_{50} = 0.27 µM	O=C1OCC2=C1C(C3=CC4=C(C=C3N2)OCO4)/C=C/C5=CC(OC)=C(OC)C(OC)=C5	51
**AS78**	CHEMBL114642	IC_{50} = 2.3 µM	O=C1C2=C(C=CC=C2)C(CC3=CC=C(OC)C(O)=C3)C4=CC=CC=C41	52
**AS79**	CHEMBL392633	IC_{50} = 0.56 µM	COC1=CC=C(C=C1)S(=O)(=O)OC2=C3C=CC=CC3=CC4=CC=CC=C42	53
**AS80**	CHEMBL455138	IC_{50} = 0.52 µM	O=C1C2=C(C=CC=C2)/C(C3=CC=CC=C31)=C\C(C4=CC=C(OC)C=C4)=O	54
**AS81**	CHEMBL1210677	IC_{50} = 1.5 µM	O=C1C2=C(C=CC=C2)/C(C3=CC=CC=C31)=N\OCC4=CC=C(OC)C=C4	55
**AS82**	CHEMBL1814612	IC_{50} = 0.44 µM	O=C1C2=C(C=CC=C2)/C(C3=CC=CC=C31)=N\C4=CC=C(OC)C(O)=C4	56
**AS83**	CHEMBL1814615	IC_{50} = 1.11 µM	O=C1C2=C(C=CC=C2Cl)/C(C3=CC=CC(Cl)=C31)=N\C4=CC=C(OC)C(O)=C4	56
**AS84**	CHEMBL4585656	51.0 ± 11.2 % inhibition at 10 µM	OC1=C(C)C=C2C([C@H](C)CC/C=C(C)\C2)=C1	57
**AS85**	CHEMBL4551469	56.3 ± 7.5 % inhibition at 10 µM	C[C@H]1C2=CC(OC(C3=CC=CC=C3)=O)=C(C)C=C2C/C(C)=C\CC1	57
**AS86**	2301070-52-8	58.1 ± 25.3 % inhibition at 10 µM	OC1=C(C)C=C2C([C@H](C)CCC/C(C)=C\2)=C1	57
**AS87**	CHEMBL4590152	38.7 ± 25.4 % inhibition at 10 µM	C[C@H]1C2=CC(OC(C3=CC=CC=C3)=O)=C(C)C=C2/C=C(C)\CCC1	57

**AS88**	CHEMBL4542864	46.9 ± 5.3 % inhibition at 10 µM	OC1=C(C)C=C2C([C@H](C)CC[C@H]3[C@](C)(O3)C2)=C1	[57]
**AS89**	CHEMBL4550701	47.9 ± 8.1 % inhibition at 10 µM	C[C@H]1C2=CC(OC(C3=CC=CC=C3)=O)=C(C)C=C2C[C@@](C)(O4)[C@@H]4CC1	[57]
**AS90**	CHEMBL4553976	30.3 ± 5.4 % inhibition at 10 µM	OC1=C(C)C=C2C([C@H](C)CCC[C@@]3(C)[C@@H]2O3)=C1	[57]
**AS91**	CHEMBL4538683	97.9 ± 15.3 % inhibition at 10 µM	C[C@H]1C2=CC(OC(C3=CC=CC=C3)=O)=C(C)C=C2[C@@H](O4)[C@]4(C)CCC1	[57]
**AS92**	CHEMBL4567416	43.7 ± 27.5 % inhibition at 10 µM	OC1=C(C)C=C2C([C@](C)(O3)CC[C@H]3[C@H](C)C2)=C1	[57]
**AS93**	CHEMBL4582836	26.1 ± 15.8 % inhibition at 10 µM	C[C@]1(O2)C3=CC(OC(C4=CC=CC=C4)=O)=C(C)C=C3C[C@@H](C)[C@@H]2CC1	[57]
**AS94**	CHEMBL4578873	38.7 ± 2.5 % inhibition at 10 µM	OC1=C(C)C=C2C([C@@]3(C)CCC[C@H](C)[C@@H]2O3)=C1	[57]
**AS95**	CHEMBL4593906	9.2 ± 4.1 % inhibition at 10 µM	C[C@]12C3=CC(OC(C4=CC=CC=C4)=O)=C(C)C=C3[C@@H](O2)[C@@H](C)CCC1	[57]

2. Description and optimisation of pharmacophore models

Model SB1

Pharmacophore SB1 was derived from Nocodazole bound to chain B of porcine tubulin (PDB: 5CA1).[58] Based on the automatically generated pharmacophore model, removal and tolerance adjusting of features and Xvols coat modifications, led to the model consisting of two HC features and one HBD (interaction with GLU198B). LB pharmacophore features from active compounds **AS2**, **AS7**, **AS8**, **AS10** , and **AS11** were merged, and the feature coordinates were extracted and manually added to model SB1, leading to the final model consisting of an additional HBD, two HBAs, two AI features and eleven Xvols.

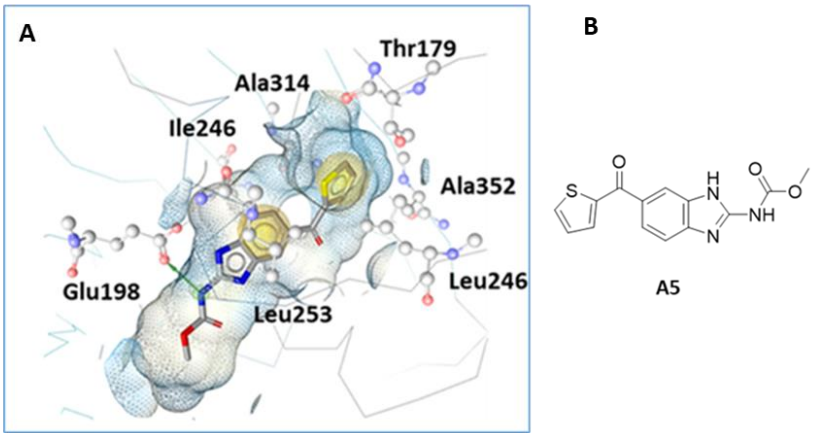

Figure S1. A) Automatically generated pharmacophore model of Tubulin CBS 3D crystal structure complex with nocodazole **AS5** based on (PDB 5CA1)[58], B) Structure of the ligand nocodazole **AS5**.

Model SB2

Pharmacophore model SB2 was derived from [2-(1H-indol-4-yl)-1H-imidazol-4-yl](3,4,5-trimethoxyphenyl)methanone bound to chain B of bovine tubulin (PDB: 6O5M).[59] Based on the automatically generated pharmacophore model, removal and tolerance adjusting of features and Xvols coat modifications, led to the model consisting of one HC feature, three HBAs towards HOH67B and Cys239B, two HBDs (interactions with Thr179A, ASN347B), and 18 Xvols.

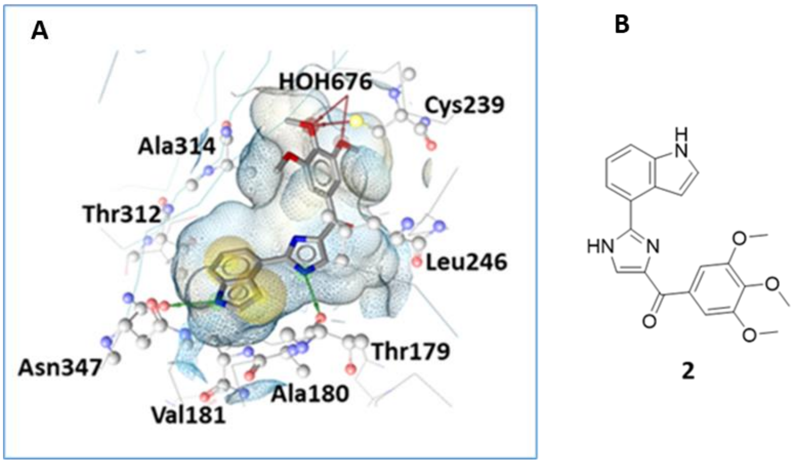

Figure S2. A) Automatically generated pharmacophore model of Tubulin CBS 3D crystal structure complex with the indol-imidazol-trimethoxypheny **2** based on (PDB 6O5M)[59], B) Structure of the ligand **2**.

Model LB3

Pharmacophore model LB3 was developed by automatic clustering of **AS55**, **AS56**, **AS57**, **AS63**, **AS85**, **AS86**, **AS87**, **AS88**, **AS89**, **AS90**, **AS91**, **AS92**, **AS93**, **AS94**, and **AS95** and creating a merged feature pharmacophore. After automatic pharmacophore model generation, removal and tolerance adjusting of features and Xvols coat modifications were optimised to find as many actives as possible while excluding inactives. The final model contained one AI feature, three HC features, two HBAs and 36 Xvols.

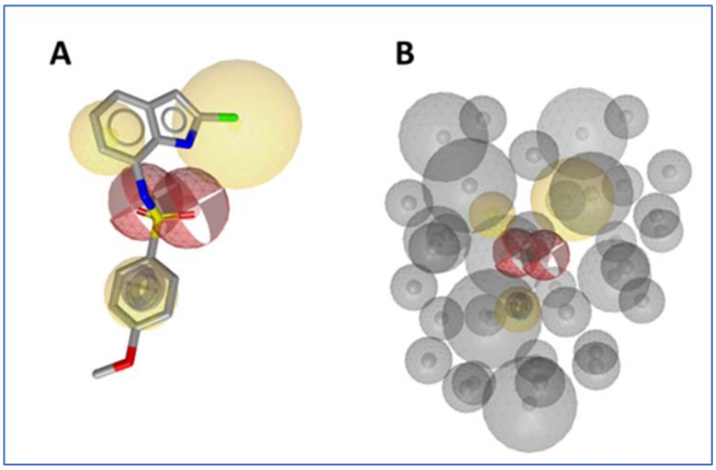

Figure S3. A) Optimised LB pharmacophore model LB3 aligned with base ligand **AS55** and B) the model LB3 with Xvols spheres. (HC, yellow spheres), (AI, purple sphere) Hydrogen bond (HBA; red arrows/spheres), and (Xvols; grey sphere).

Model LB4

Pharmacophore model LB4 was developed by automatic clustering and aligning the myosverin derivatives **AS66**, **AS67**, **AS68**, **AS69**, **AS70**, **AS71**, and **AS72** and creating a merged feature pharmacophore. After automatic pharmacophore model generation, removal and tolerance adjusting of features and Xvols coat modifications were optimised to find as many actives as possible while excluding inactives. The final model contained two HC features, five HBAs, two HBDs and 54 Xvols.

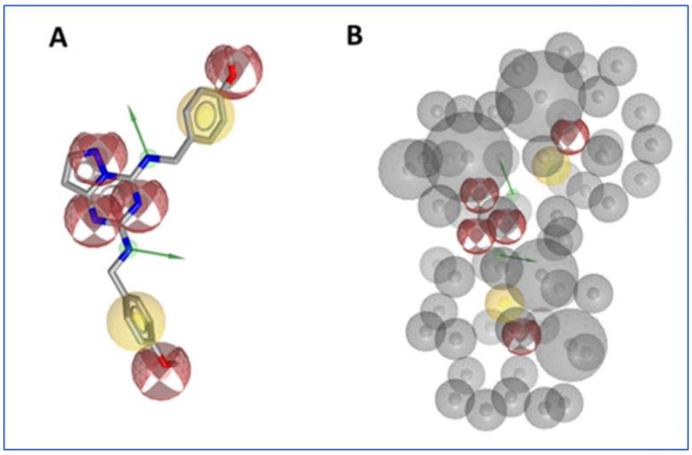

Figure S4. A) Optimised LB pharmacophore model LB4 aligned with base ligand **AS71** and B) the model LB4 with Xvol spheres. (HC, yellow spheres), (AI, purple sphere), (HBD; green arrows/spheres), Hydrogen bond acceptors (HBA; red arrows/spheres), and (Xvols; grey sphere).

Model LB5

Pharmacophore model LB5 was developed by automatic clustering and aligning the sesquiterpenoids **AS85, AS86**, **AS87**, **AS88**, **AS89**, **AS90**, **AS91**, **AS92**, **AS93**, and **AS94** and creating a merged features pharmacophore. After automatic pharmacophore model generation, removal and tolerance adjusting of features and Xvols coat modifications were optimised to find as many actives as possible while excluding inactives. The final model contained one AI feature, three HC features, one HBA, and 49 Xvols.

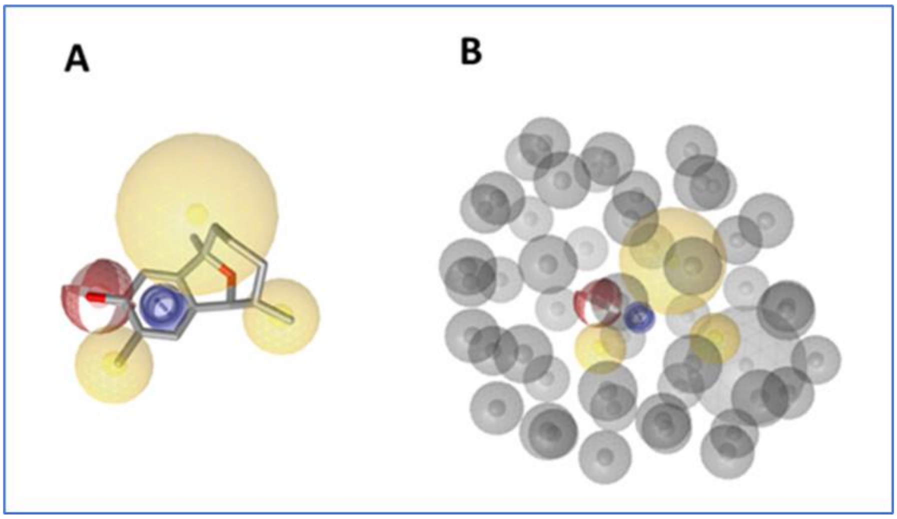

Figure S5. A) Optimised LB pharmacophore model LB5 aligned with base ligand **AS94** and B) the model LB5 with Xvols spheres. (HC, yellow spheres), (AI, purple sphere), (HBA; red arrows/spheres), and (Xvols; grey sphere).

Model LB6

Pharmacophore model LB6 was developed by manually selecting and aligning the indole-sulfonamide derivatives **AS58**, **AS59**, **AS60**, and **AS61**, creating a shared features pharmacophore. After automatic pharmacophore model generation, removal and tolerance adjusting of features and Xvols coat modifications were optimised to find as many actives as possible while excluding inactives. The final model contained two AI features, two HC features, two HBAs, one HBD and 35 Xvols.

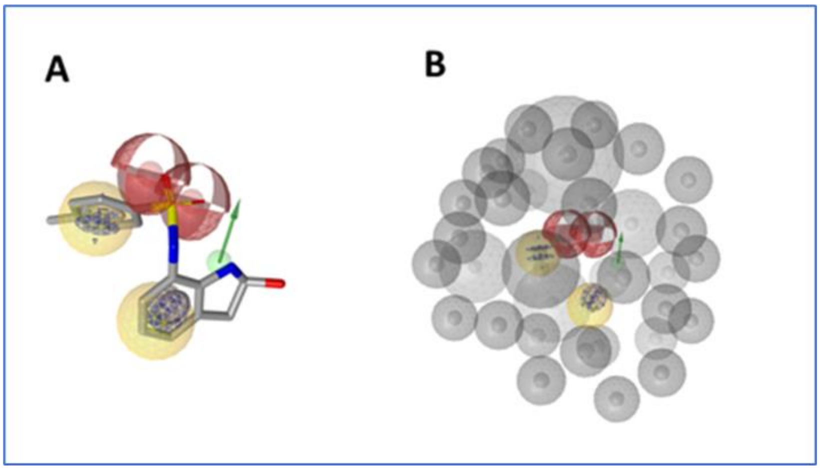

Figure S6. A) Optimised LB pharmacophore model LB6 aligned with base ligand **AS59** and B) the model LB6 with Xvol spheres. (HC, yellow spheres), (AI, purple sphere), (HBD; green arrows/spheres), (HBA; red arrows/spheres), and (Xvols; grey sphere).

Model LB7

Pharmacophore model LB7 was developed by manually selecting and aligning **AS30**, **AS33**, **AS34**, **AS75**, and **AS77** and creating a merged features pharmacophore. After automatic pharmacophore model generation, removal and tolerance adjusting of features and Xvols coat modifications were optimised to find as many actives as possible while excluding inactives. The final model contained one AI feature, one HC feature, four HBAs, one HBD and 68 Xvols.

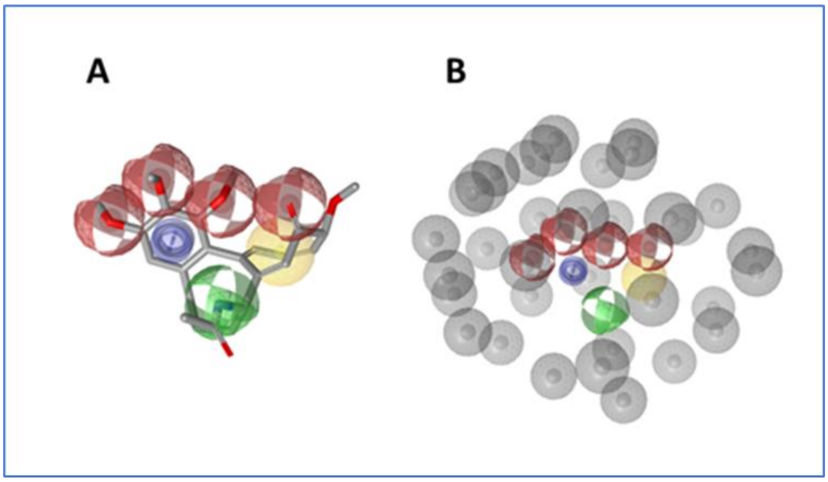

Figure S7. A) Optimised LB pharmacophore model LB7 aligned with base ligand **AS30** and B) the model LB7 with Xvols spheres. (HC, yellow spheres), (AI, purple sphere), (HBD; green arrows/spheres), (HBA; red arrows/spheres), and (Xvols; grey sphere).

Model LB8

Pharmacophore model LB8 was developed bymanually selectingand aligning **AS1**, **AS3**, **AS8**, **AS12**, **AS13**, **AS14**, **AS15**, **AS16**, and **AS17** and creating a merged features pharmacophore. After automatic pharmacophore model generation, removal and tolerance adjusting of features and Xvols coat modifications were optimised to find as many actives as possible while excluding inactives. The final model contained one AI feature, two HC features, two HBAs and 30 Xvols.

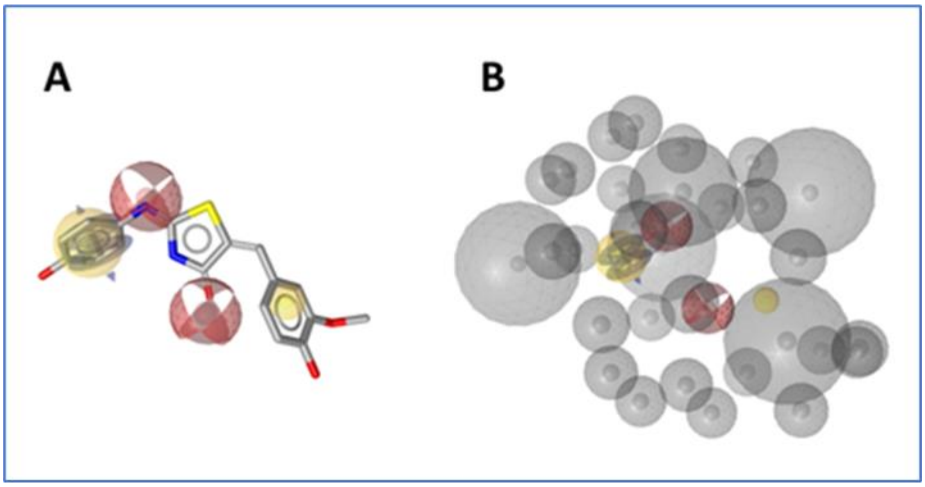

Figure S8. A) Optimised LB pharmacophore model LB8 aligned with base ligand **AS15** and B) the model LB8 with Xvol spheres. (HC, yellow spheres), (AI, purple sphere), (HBA; red arrows/spheres), and (Xvols; grey sphere).

Model LB9

Pharmacophore model LB9 was developed by manually selecting and aligning **AS3**, **AS8**, **AS12**, and **AS13** and creating a merged features pharmacophore. After automatic pharmacophore model generation, removal and tolerance adjusting of features and Xvols coat modifications were optimised to find as many actives as possible while excluding inactives. The final model contained two AI features, one HC feature, three HBAs and 39 Xvols.

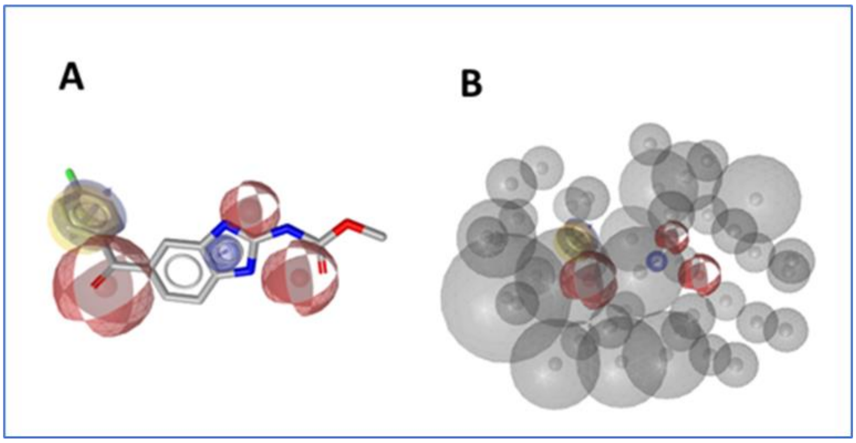

Figure S9. A) Optimised LB pharmacophore model LB9 aligned with base ligand **AS8** and B) the model LB9 with Xvols spheres. (HC, yellow spheres), (AI, purple sphere), (HBA; red arrows/spheres), and (Xvols; grey sphere).

Model LB10

Pharmacophore model LB10 was developed by automatic clustering and aligning **AS79**, **AS80**, **AS81**, and **AS82** creating a merged features pharmacophore. After automatic pharmacophore model generation, removal and tolerance adjusting of features and Xvols coat modifications were optimised to find as many actives as possible while excluding inactives. The final model contained two AI features, two HC features, two HBAs and 45 Xvols.

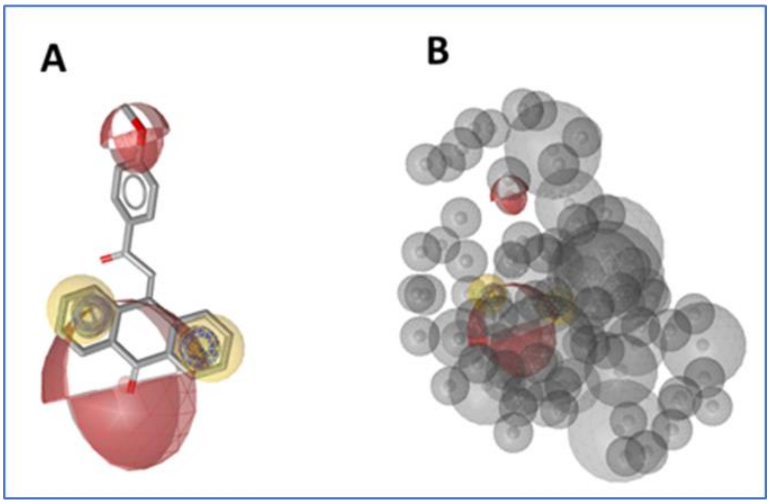

Figure S10. A) Optimised LB pharmacophore model LB10 aligned with base ligand **AS80** and B) the model LB10 with Xvol spheres. (HC, yellow spheres), (AI, purple sphere), (HBA; red arrows/spheres), and (Xvols; grey sphere).

Model LB11

Pharmacophore model LB11 was developed by manually selecting and aligning **AS13**, **AS27**, **AS43**, and **AS72** and creating a merged features pharmacophore. After automatic pharmacophore model generation, removal and tolerance adjusting of features and Xvols coat modifications were optimised to find as many actives as possible while excluding inactives. The final model contained one HC feature, four HBAs and 39 Xvols.

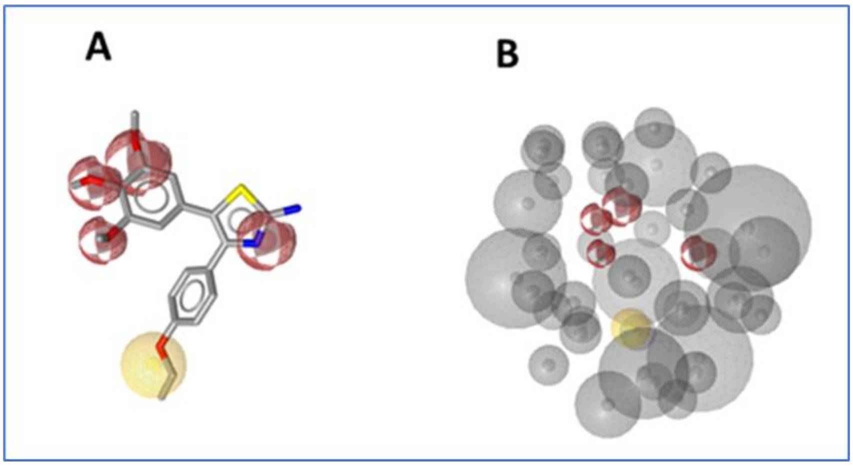

Figure S11. A) Optimised LB pharmacophore model LB11 aligned with base ligand **AS13** and B) the model LB11 with Xvol spheres. (HC, yellow spheres), (AI, purple sphere), (HBA; red arrows/spheres), and (Xvols; grey sphere).

Model DS2

The model DS2 was based on **AS64**, a diphenyl sulfonamide. It contained three HC features, two AI features that are mapped on the two phenyl rings, two HBD features and 26 Xvols.

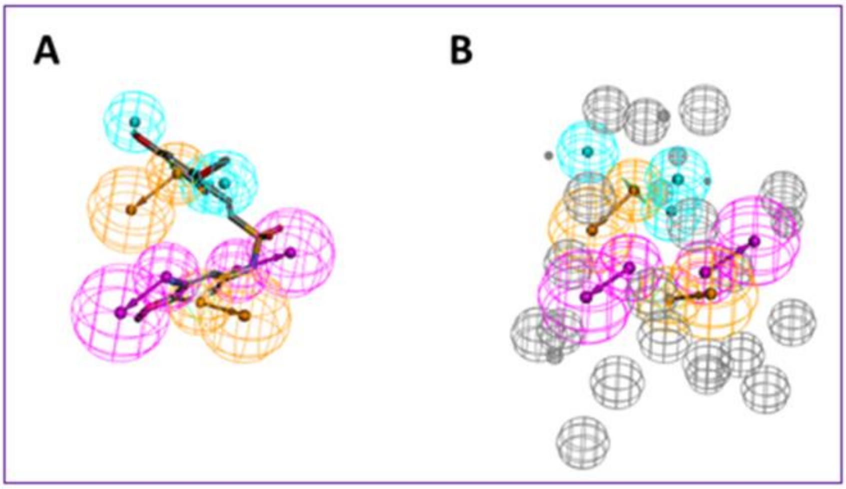

Figure S12. A) Optimised LB pharmacophore model DS2 aligned with base ligand **AS64**. B) the model DS2 with Xvol spheres. (HC, blue spheres), (AI, brown spheres), (HBD; purple), and (Xvols; grey sphere).

Model DS5

Model DS5 was based on **AS75**, a podophyllotoxin. It contains four HC features, two AI features, five HBA features and 34 Xvols.

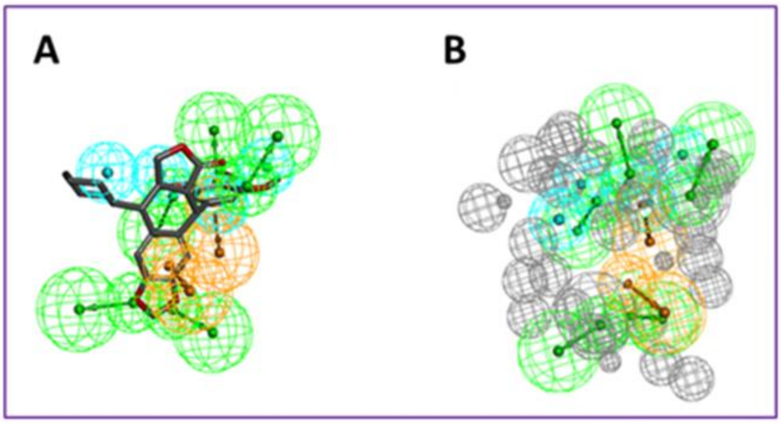

Figure S13. A) Optimised LB pharmacophore model DS5 aligned with base ligand **AS75**. B) the model DS5 with Xvol spheres. (HC, blue spheres), (AI, brown spheres), (HBA; green sphere), and exclusion volumes (Xvols; grey sphere).

Model DS8

The model DS8 was calculated for **AS77**, a modified podophyllotoxin scaffold with an amine functionality inserted in the central ring and an elongated linker to the trimethoxyphenylring.

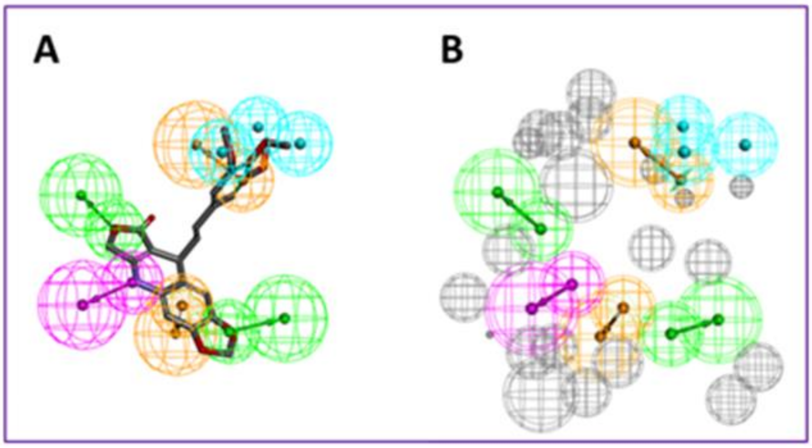

Figure S14. A) Optimised LB pharmacophore model DS8 aligned with base ligand **AS77** .B) the model DS8 with Xvol spheres. (HC, blue spheres), (AI, brown spheres), (HBA; green sphere) (HBD; purple sphere), and (Xvols; grey sphere).

Model DS9

Model DS9 was derived from **AS79**, an anthracene sulfonate. It consists of a HC feature, three AI features, two HBA features and 60 Xvols.

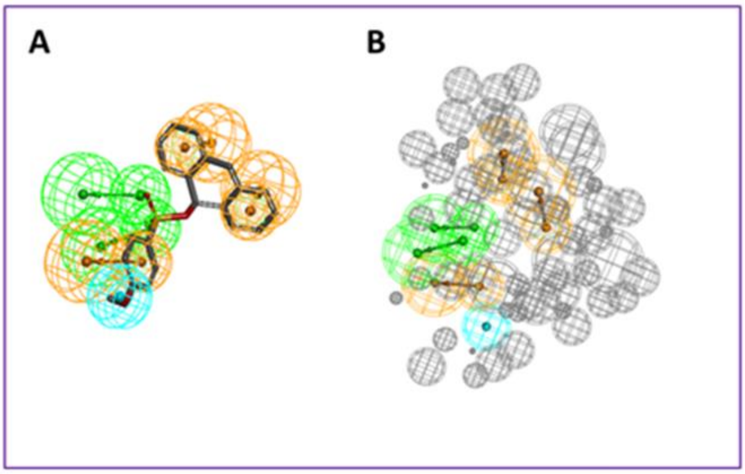

Figure S15. A) Optimised LB pharmacophore model DS9 aligned with base ligand **AS79**. B) the model DS9 with Xvol spheres. (HC, blue spheres), (AI, brown spheres), (HBA; green sphere), and (Xvols; grey sphere).

Model DS12

The model DS12 was based on **AS29**, a chalcone. The final model contains two HC, two AI, two HBA features and 118 Xvols.

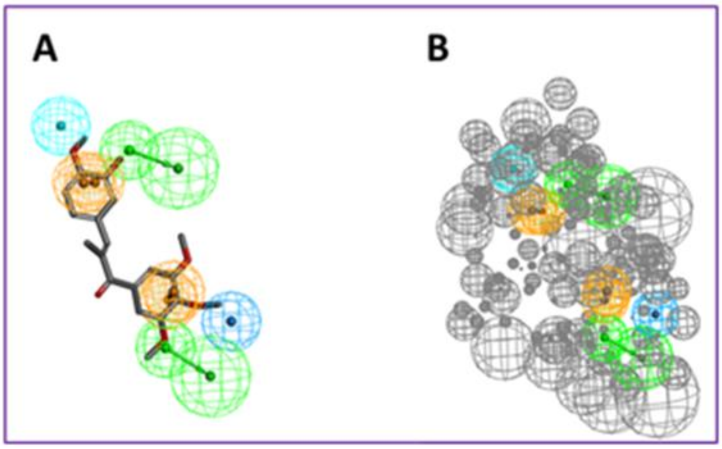

Figure S16. A) Optimised LB pharmacophore model DS12 aligned with base ligand **AS29**. B) the model DS12 with Xvol spheres. (HC, blue spheres), (AI, brown spheres), (HBA; green sphere), and (Xvols; grey sphere).

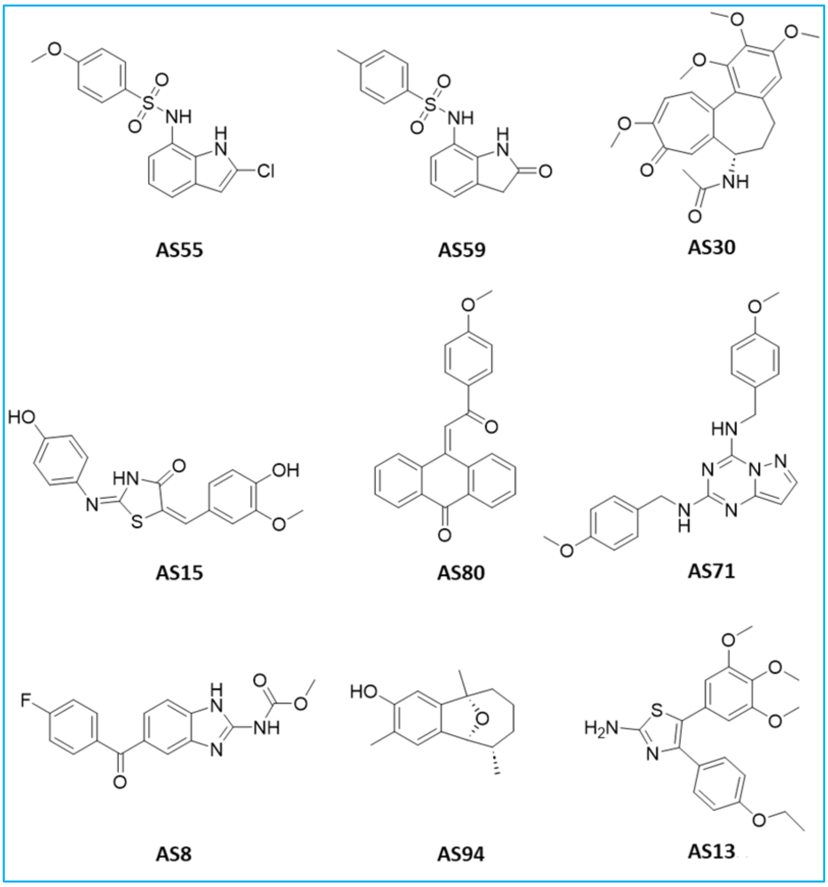

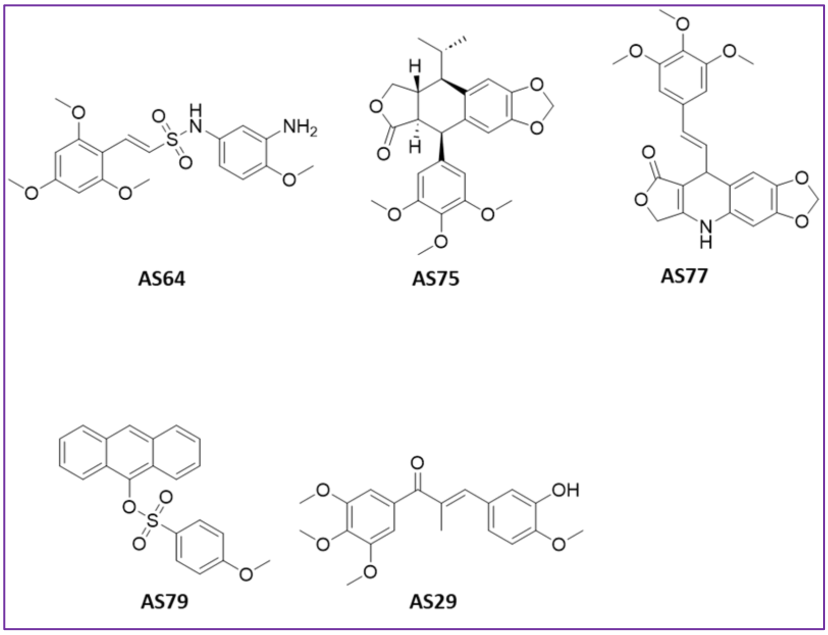

Figure S17. Chemical structures of the main active scaffolds used to generate the LB pharmacophore models. Compounds in the blue box were used for the LS models and compounds in the purple box were used for the DS models.

3. Virtual screening of pharmacophore models with consensus hits

Table S2. Virtual screening results with consensus hits obtained by the respective pharmacophore models.

	**Consensus hit**	**(n=2)**	
Models	Specs (Synth)	Specs (NP)	PhytoChem (PC)
DS2/DS8	0	0	1
DS5/DS8	0	3	0
DS8/DS9	4	1	0
DS5/DS9	9	0	0
DS2/DS9	13	0	0
DS12/DS9	5	0	0
DS12/DS2	3	0	0
LB8/DS9	3	0	0
LB8/DS2	1	0	0
LB8/DS12	2	0	0
LB7/DS9	2	0	0
LB6/DS9	1	0	0
LB5/DS9	19	0	0
LB5/DS2	10	0	0
LB5/DS12	3	0	0
LB5/DS8	4	0	0
LB5/DS6	3	0	0
LB3/DS9	46	0	0
LB3/DS5	1	0	0
LB3/DS2	5	0	0
LB3/DS12	3	0	0
LB3/LB9	1	0	0
LB3/LB8	4	0	0
LB3/LB6	16	0	0
LB3/LB5	167	0	0
SB2/LB5	4	0	0
LB11/DS9	6	0	0
LB11/DS2	1	0	0
LB11/DS12	1	0	0
LB11/LB9	1	0	0
LB11/LB8	1	0	0
LB11/LB7	1	0	0

LB11/LB6	6	0	0
LB11/LB5	5	0	0
LB11/LB3	6	0	0
LB11/SB2	3	0	0
LB10/DS9	6	0	0
LB10/DS5	5	0	0
LB10/LB3	2	0	0
LB10/LB11	3	0	0
SB1/LB5	4	0	0
SB1/LB3	1	0	0
	**Consensus hit**	**(n=3)**	
Models	Specs (Synth)	Specs (NP)	PhytoChem (PC)
DS5/DS8/DS9	0	3	0
LB10 / LB5 /DS9	1	0	0
LB10 / DS9/DS12	2	0	0
LB11 /LB6/DS2	1	0	0
LB3/LB5/LB8	1	0	0
LB3/LB5/DS9	2	0	0
LB5/DS2/DS8	1	0	0
DS2/DS5/DS9	1	0	0
LB3/LB5/DS2	1	0	0

4. Test compound selection of hits obtained from virtual screening of the databases.

Table S3. Virtual screening hits selected for experimental validation. Red box indicates insoluble compounds or fluorescent interference and thus excluded compounds.

Specs	Virtual Hit	SB1	SB2	LB3	LB4	LB5	LB6	LB7	LB8	LB9	LB10	LB11	DS2	DS5	DS8	DS9	DS12
AA-504/21163091	SC1													X			
AB-323/13887441	SC2		X														
AC-776/41252593	SC3													X			
AF-399/14738025	SC4								X								
AG-205/13184005	SC5			X													
AG-205/21054014	SC6															X	
AG-205/36265044	SC7	X (red)								X							
AG-389/15452424	SC8																
AG-401/30827061	SC9							X (red)	X (red)								X (red)
AG-690/09788017	SC10										X (red)						
AJ-292/21122017	SC11													X			
AJ-916/12583009	SC12					X (red)			X (red)								
AK-087/42718249	SC13					X											
AK-087/42718376	SC14															X	
AK-693/40962733	SC15					X											

AK-968/41922685	SC16	X															
AM-807/13614425	SC17	X		X													
AN-329/43211079	SC18							X									
AN-465/14334022	SC19									X							
AN-465/41674183	SC20							X									
AN-648/42098983	SC21		X														
AN-652/12103469	SC22							X									
AO-022/43514129	SC23		X														
AO-313/21215019	SC24													X	X	X	
AO-365/43300818	SC25										X					X	X
AO-365/43300823	SC26			X		X											
AO-365/43300935	SC27								X								
AO-365/43474517	SC28											X				X	
AO-365/43486487	SC29									X							
AO-476/15509049	SC30										X						
AP-501/43179291	SC31										X	X					
AP-263/40720502	SC32						X										
AP-123/40765218	SC33												X				
AP-263/43418357	SC34						X					X	X				
AP-845/42065522	SC35									X							
AP-853/42655770	SC36						X										
AP-853/43386817	SC37						X										

**AQ-086/43467809**	**SC38**	X															
**AT-057/43469212**	**SC39**																
**AT-057/43485534**	**SC40**							X									
**AT-583/41299652**	**SC41**														X		
**AH-487/41949628**	**SC42**																X
**AG-690/12094002**	**SC43**			X					X								
**AI-204/31688027**	**SC44**						X										
**AG-690/12413358**	**SC45**	X		X													
**AI-899/21033027**	**SC46**										X						

5. Bioactivity results of selected test compounds from the polymerisation inhibition assay and similarity prediction

Table S4. Overview of the tubulin polymerisation inhibition screening results.

Specs ID-number	ID	% Inhibition (30 µM) ± SD	% Inhibition (10 µM) ± SD *20 µM	IC50 (µM)
AA-504/21163091	SC1	28.8 ± 22.7	n.d	n.d
AB-323/13887441	SC2	≤ 0	n.d	n.d
AC-776/41252593	SC3	Insoluble	≤ 0	n.d
AF-399/14738025	SC4	7.0 ± 8.1	n.d	n.d
AG-205/13184005	SC5	≤ 0	n.d	n.d
AG-205/21054014	SC6	38.4 ± 12.6	n.d	n.d
AG-205/36265044	SC7	insoluble	insoluble	n.d
AG-389/15452424	SC8	Insoluble	≤ 0	n.d
AG-401/30827061	SC9	Insoluble	Insoluble	n.d
AG-690/09788017	SC10	Insoluble	Insoluble	n.d
AJ-292/21122017	SC11	≤ 0	n.d	n.d
AJ-916/12583009	SC12	insoluble	insoluble	n.d
AK-087/42718249	SC13	≤ 0	n.d	n.d
AK-087/42718376	SC14	insoluble	43.9 ± 23.9	n.d
AK-693/40962733	SC15	n.d	≤ 0	n.d
AK-968/41922685	SC16	n.d	≤ 0	n.d
AM-807/13614425	SC17	n.d	1.7 ± 3.1	n.d
AN-329/43211079	SC18	n.d	n.d	n.d
AN-465/14334022	SC19	n.d	≤ 0	n.d
AN-465/41674183	SC20	19. 2 ± 24.9	n.d	n.d
AN-648/42098983	SC21	n.d	1.29 ± 1.28	n.d
AN-652/12103469	SC22	54.7 ± 2.5	n.d	n.d
AO-022/43514129	SC23	99.3 ± 3.72	86.5 ± 2.7	Yes
AO-313/21215019	SC24	insoluble	*95.50 ± 2.5	n.d
AO-365/43300818	SC25	25.5 ± 14.6	n.d	n.d
AO-365/43300823	SC26	insoluble	37.6 ± 35.9	n.d
AO-365/43300935	SC27	32.87 ± 1.69	36.0 ± 4.4	n.d
AO-365/43474517	SC28	Insoluble	2.33 ± 1.8	n.d
AO-365/43486487	SC29	8.69 ± 4.42	n.d	n.d
AO-476/15509049	SC30	Insoluble	Insoluble	n.d
AP-501/43179291	SC31	Insoluble	≤ 0	n.d
AP-263/40720502	SC32	73.9 ± 0.2	n.d	n.d
AP-123/40765218	SC33	≤ 0	n.d	n.d

AP-263/43418357	SC34	37.8 ± 5.7	n.d	n.d
AP-845/42065522	SC35	Insoluble	7.6 ± 8.0	n.d
AP-853/42655770	SC36	Insoluble	≤ 0	n.d
AP-853/43386817	SC37	100.3 ± 0.4	71.3 ± 6.4	Yes
AQ-086/43467809	SC38	Insoluble	6.7 ± 1.0	n.d
AT-057/43469212	SC39	Insoluble	≤ 0	n.d
AT-057/43485534	SC40	Insoluble	3.9 ± 7.3	n.d
AT-583/41299652	SC41	≤ 0	n.d	n.d
AH-487/41949628	SC42	≤ 0	n.d	n.d
AG-690/12094002	SC43	Insoluble	11.3 ± 3.5	n.d
AI-204/31688027	SC44	8.84 ± 1.45	n.d	n.d
AG-690/12413358	SC45	Insoluble	17.7 ± 1.0	n.d
AI-899/21033027	SC46	8.3 ± 2.4	n.d	n.d
MERCK Group	Hit47	≤ 0	n.d	n.d
TCI Chemicals	Colchicine	98.8 ± 5.2	83.2 ± 1.7	YES

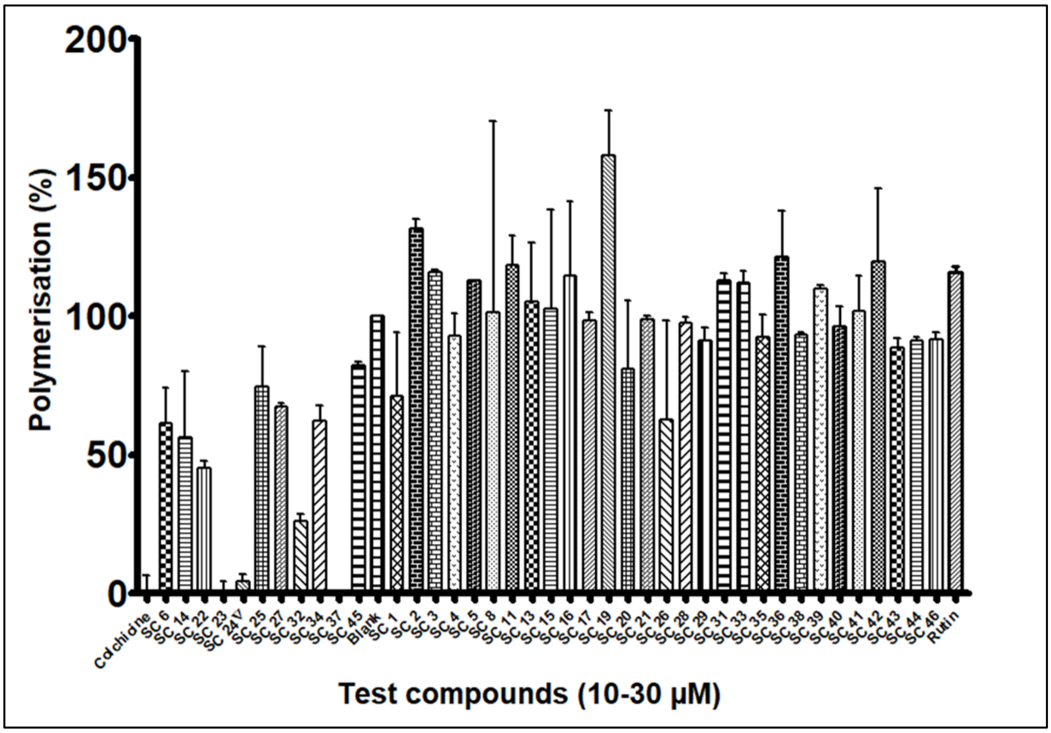

Figure S18. Graphical results of the active/inactive preliminary screening. Corresponding numerical values are shown above in Table S4.

Table S5. SwissTarget Similarity prediction scores for the tubulin inhibitors

Inhibitor	Tubulin-Ligand Probability Score	Best Target Probability score	CHEMBL Target ID
SC6	none	Carbonic anhydrase II (0.04)	CHEMBL205
SC14	CHEMBL195840 (0.62)	-	CHEMBL1915
SC22	none	Phosphodiesterase 5A (0.11)	CHEMBL1827
SC23	none	Acetyl-CoA carboxylase 2 (0.11)	CHEMBL4829
SC24	CHEMBL363063 (0.90)	-	CHEMBL1915
SC25	none	NAD-dependent deacetylase sirtuin 1 (0.14)	CHEMBL4506
SC27	none	NAD-dependent deacetylase sirtuin 1 (0.21)	CHEMBL4506
SC32	none	Metabotropic glutamate receptor 5 (0.11)	CHEMBL3227
SC34	none	Microtubule-associated protein tau (0.79)	CHEMBL1293224
SC37	none	Monoamine oxidase B (0.11)	CHEMBL2039
SC45	none	Histone deacetylase 1 0.12)	CHEMBL325

6. Active compound characterization provided by Specs

Table S6. List of compound characterization data for the found active inhibitors provided by SPECS.

ID	Specs ID	QC Method	Purity	MW	SMILES
SC6*	AG-205/210 54014	LC-MS	>95%	730.7	CC1=CC(=O)Oc2cc(ccc12)OC6OC(COS(O)(=O)=O)C(OC(=O)c3ccccc3)C(OC(=O)c4ccccc4)C6(OC(=O)c5ccccc5)
SC14	AK-087/427 18376	H-NMR,LC-MS	>95%	346.38	Oc3ccc(C(=O)C=Cc2ccccc2(OCc1ccccc1))c(O)c3

SC22	AN-652/12103469	H-NMR,LC-MS	95%	401.46	CCOC(=O)c2ccccc2(NC(=O)c1cc(OCC)c(OCC)c(c1)OCC)
SC23	AO-022/43514129	LC-MS	>95%	445.4	O=C(CC1NC(=O)c2ccccc2(NC1(=O)))NCCn3c4ccccc4(nc3C(F)(F)F)
SC24	AO-313/21215019	H-NMR,LC-MS	95%	551.98	COc1cc(cc(OC)c1(OC))C5c2cc6OCOc6(cc2C(NC(=O)c3ccccc3Cl)C4COC(=O)C45)
SC25	AO-365/43300818	LC-MS	>95%	328.35	COc1ccc(cc1)OCC3=Nn2c(nnc2S3)c4ccco4
SC27	AO-365/43300935	H-NMR,LC-MS	95%	298.33	COc1ccccc1C3=Nn2c(nnc2S3)c4ccco4
SC32	AP-263/40720502	LC-MS	>95%	449.12	CC(=O)Nc2ccccc2(OS(=O)(=O)c1cc(ccc1Br)Br)
SC34	AP-263/43418357	LC-MS	>95%	348.2	COc1c(ccc(c1Cl)Cl)S(=O)(=O)Nc2cccc(O)c2
SC37	AP-853/43386817	H-NMR,LC-MS	95%	389.82	O=C(CSc1nnc(o1)c2cccc(c2)Cl)Nc3ccc4OCOc4(c3)
SC45	AG-690/12413358	H-NMR,LC-MS	>95%	525.4	COc1ccc(cc1)NC(=O)c2ccc(cc2)Nc4nc3ccc(cc3c(n4)c5ccccc5)Br

** Stereochemistry undefined*

7. LC-MS and ^{1}H-NMR data provided by Specs

Compound **SC6**, LC-MS:

SPECS and BioSPECS B.V., The Netherlands Page 21
File:9910121054014 ID:AG-205/21054014 Description:C37H30O14S
Vial:1:21 Date:08-Nov-2001 Time:18:54:29

Printed: Thu Nov 29 09:26:51 2001

Sample Report (continued):

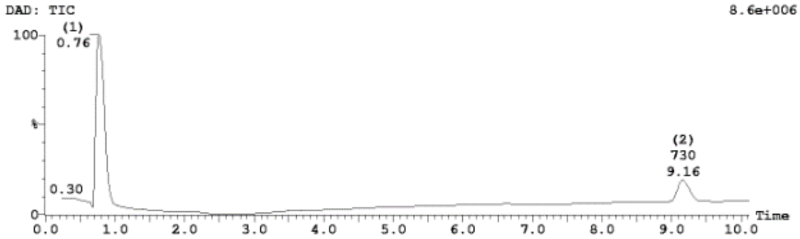

Peak Number	Time	Area %Total
1	0.76	81.66
2	9.16	18.34

Peak ID	Time	Mass Found	BPM
1	0.76		302.0

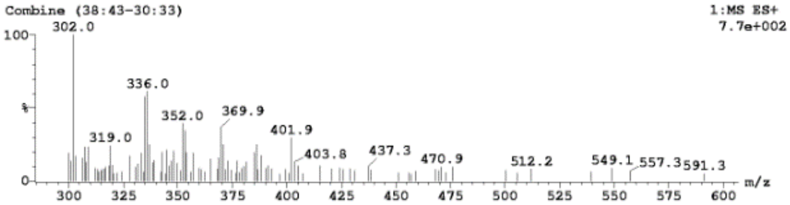

Peak ID	Time	Mass Found	BPM
2	9.16	730.14	748.3

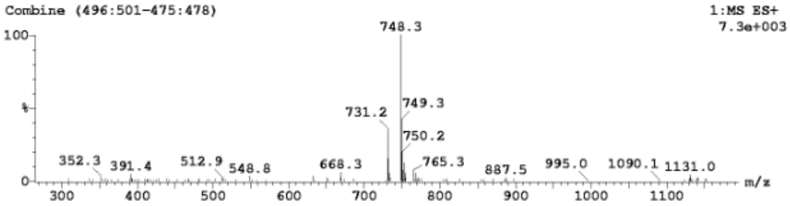

Compound **SC6**, ^{1}H-NMR:

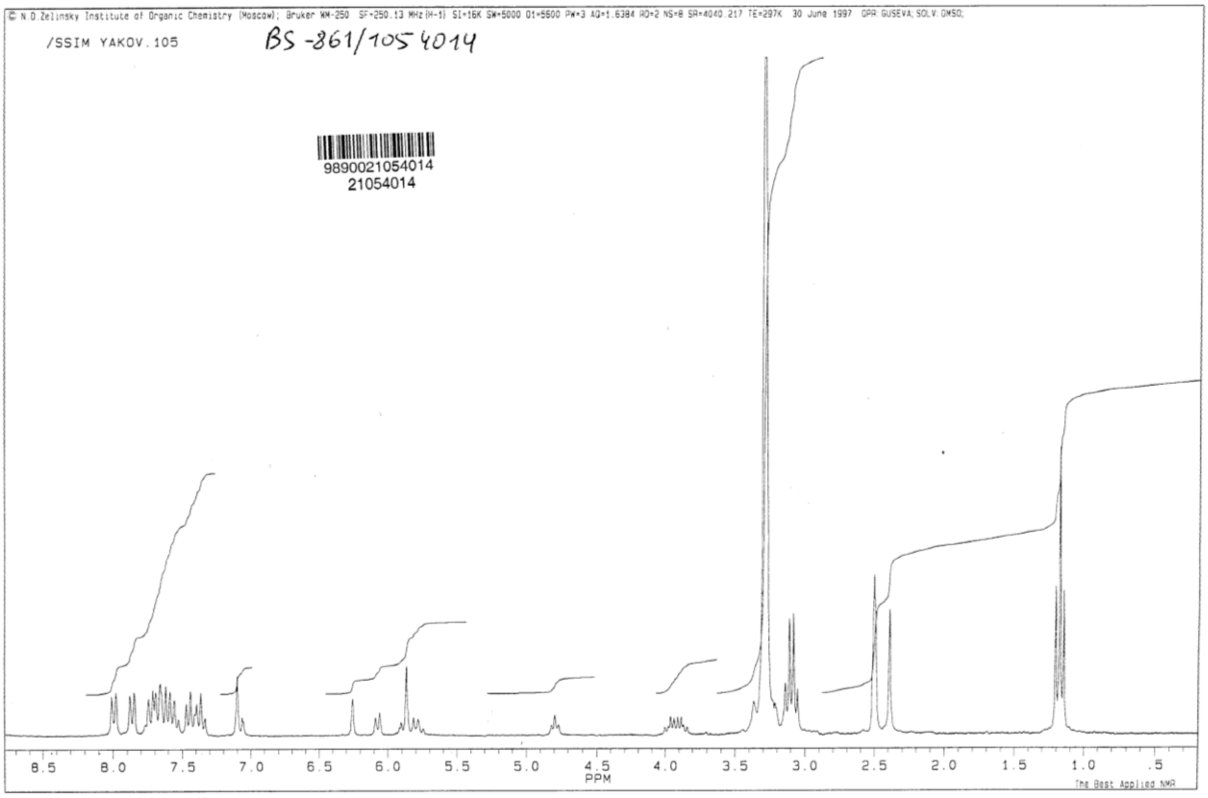

Compound **SC14**, LC-MS:

Specs Page 89
File:9900142718376 ID:AK-087/42718376 Description:C22H18O4
Instrument:LC/MS A Vial:1:89 Date:26-Feb-2004

Printed: Thu Feb 26 16:06:21 2004

Sample Report (continued):

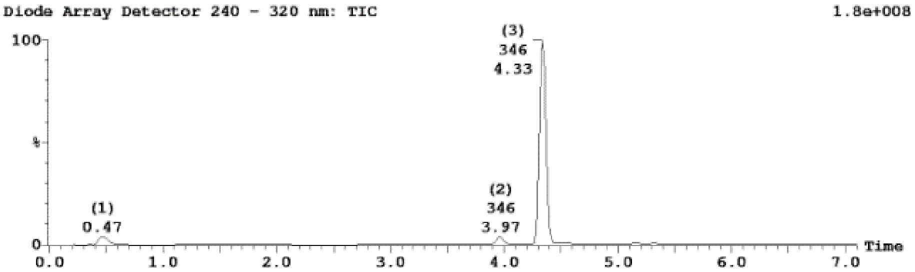

Peak Number	Time	Area %Total
1	0.47	5.46
2	3.97	3.16
3	4.33	91.38

Peak ID	Time	Mass Found	BPM
2	3.97	346.12	347

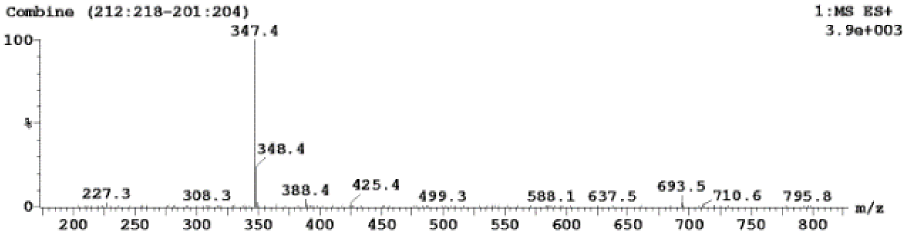

Peak ID	Time	Mass Found	BPM
3	4.33	346.12	347

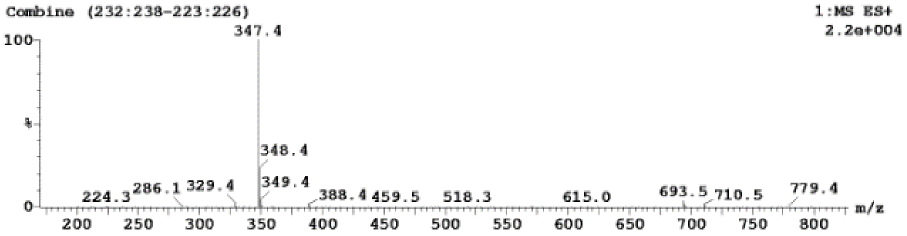

Compound **SC14**, ^{1}H-NMR:

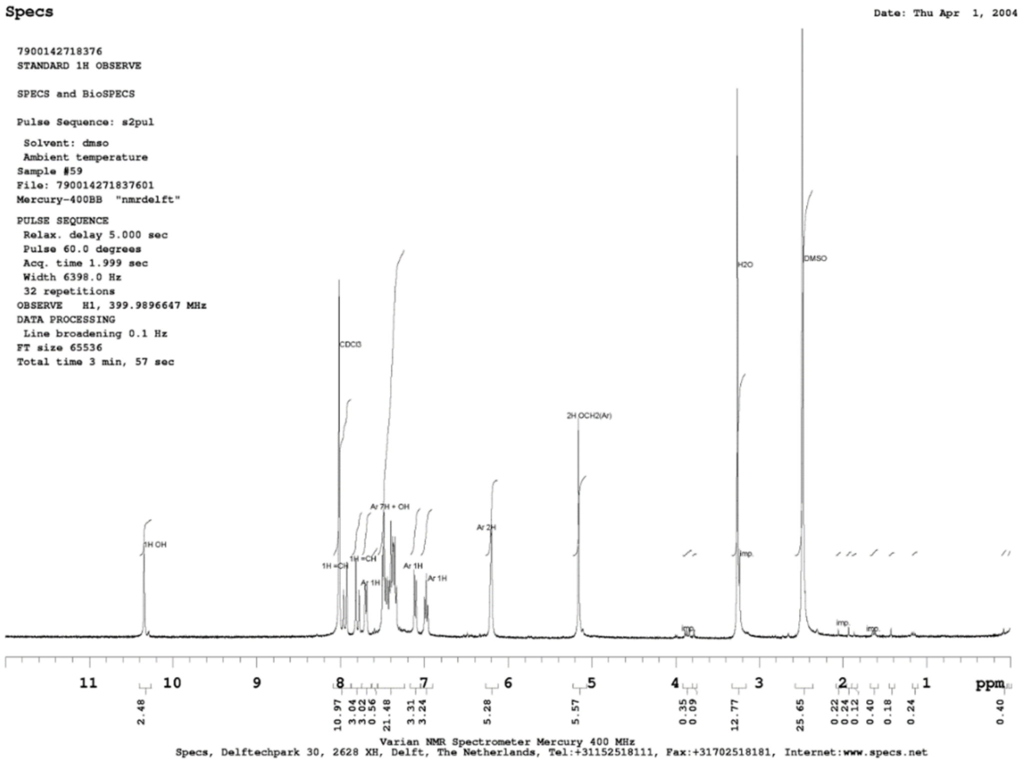

Compound **SC22**, LC-MS:

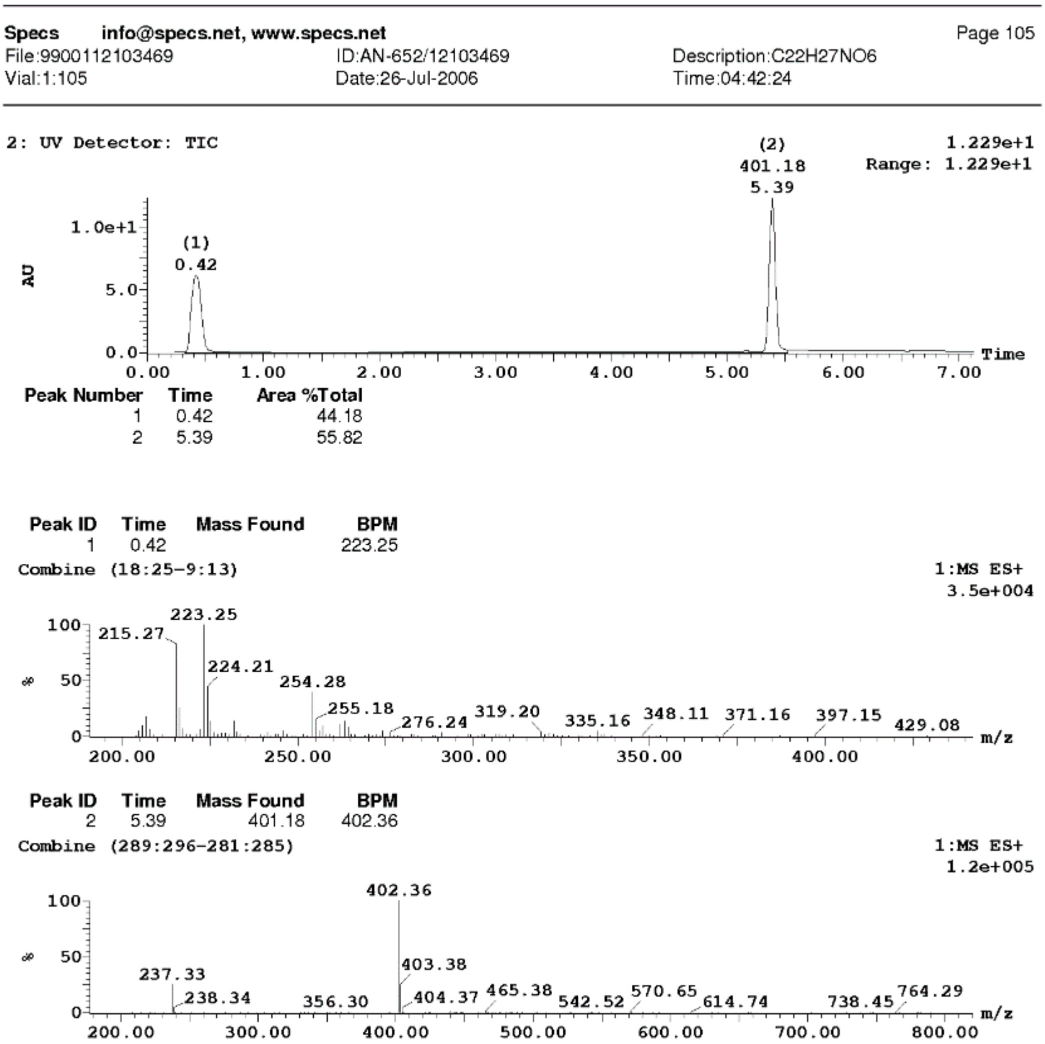

Compound **SC22**, ^{1}H-NMR:

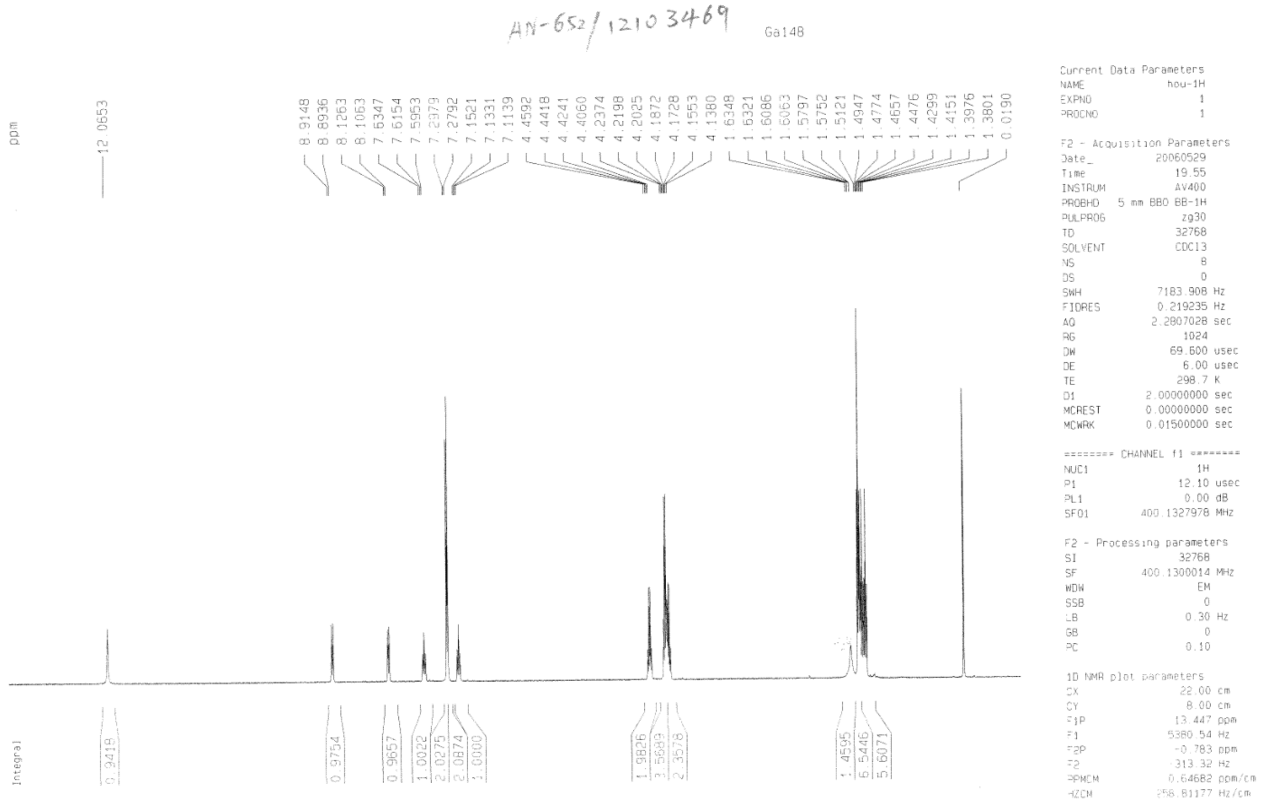

Compound **SC23**, LC-MS:

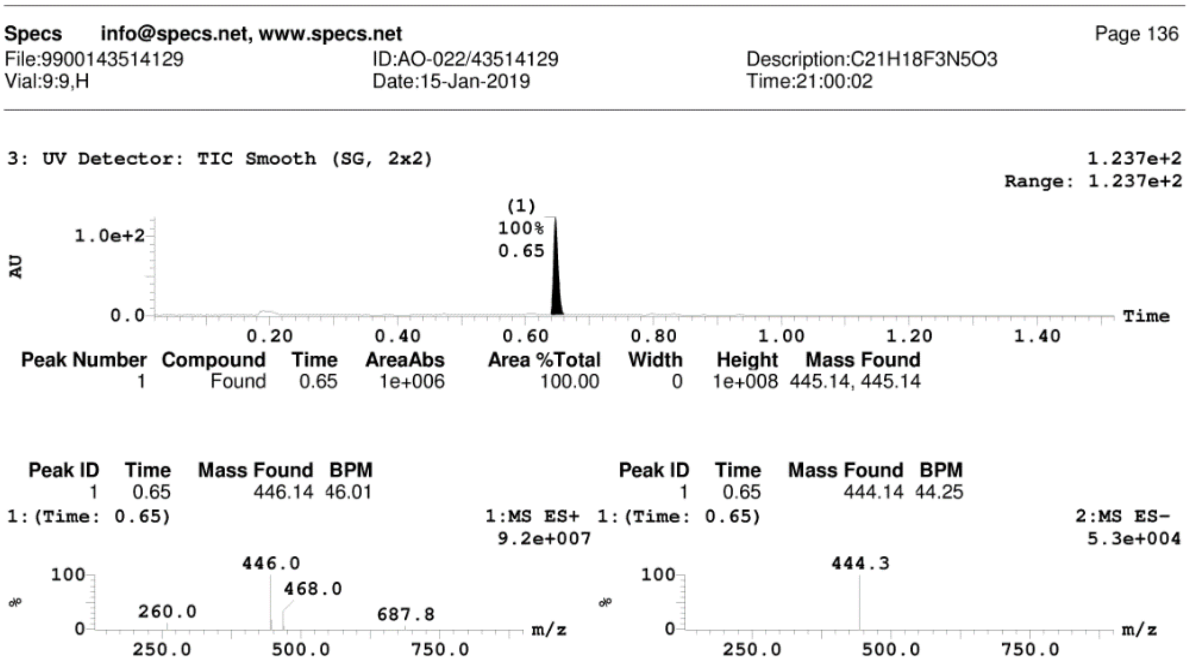

Compound **SC24**, LC-MS:

Specs **info@specs.net, www.specs.net** Page 72

File:9920121215019 ID:AO-313/21215019 Description:C29H26ClNO8

Vial:7:F,12 Date:19-Feb-2008 Time:19:52:49

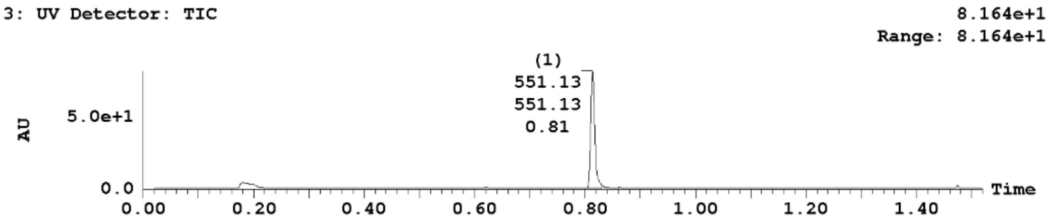

Peak Number	Time	Area %Total
1	0.81	100.00

Peak ID	Time	Mass Found	BPM
1	0.81	551.13	552.14

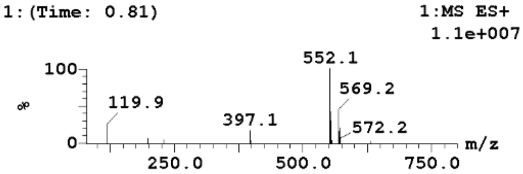

Peak ID	Time	Mass Found	BPM
1	0.81	551.13	550.29

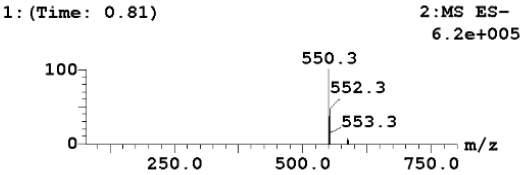

Compound **SC24**, ^{1}H-NMR:

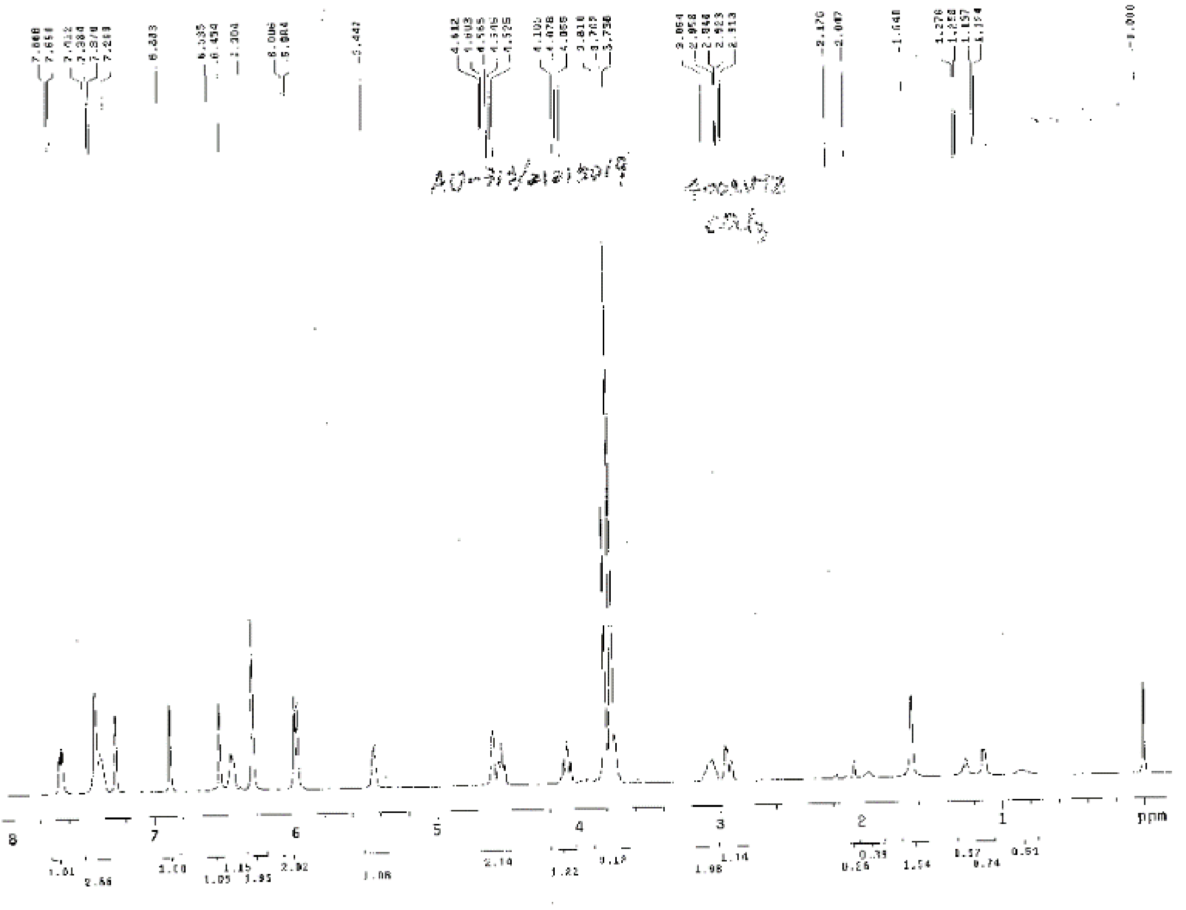

Compound **SC25**, LC-MS:

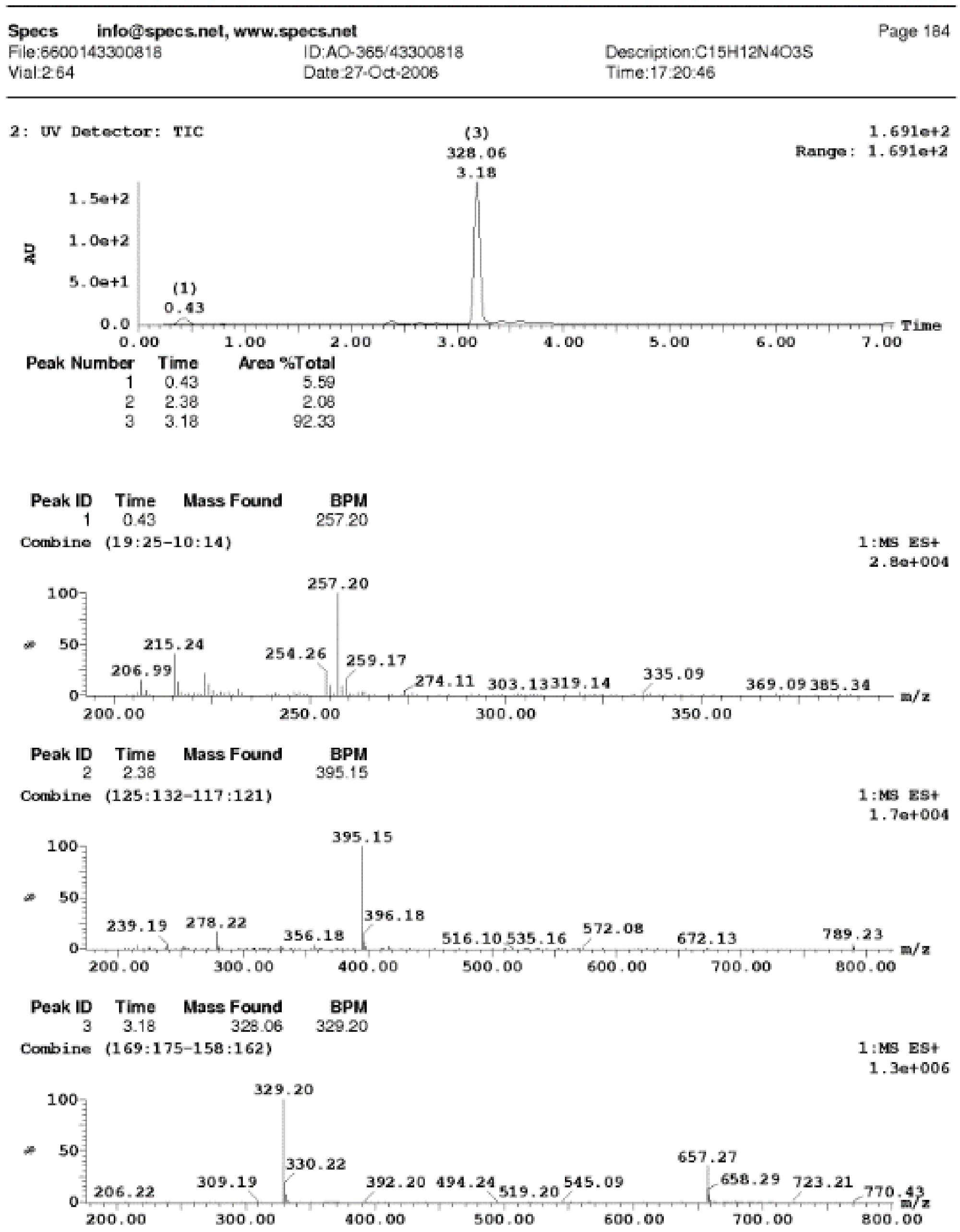

Compound **SC27**, LC-MS:

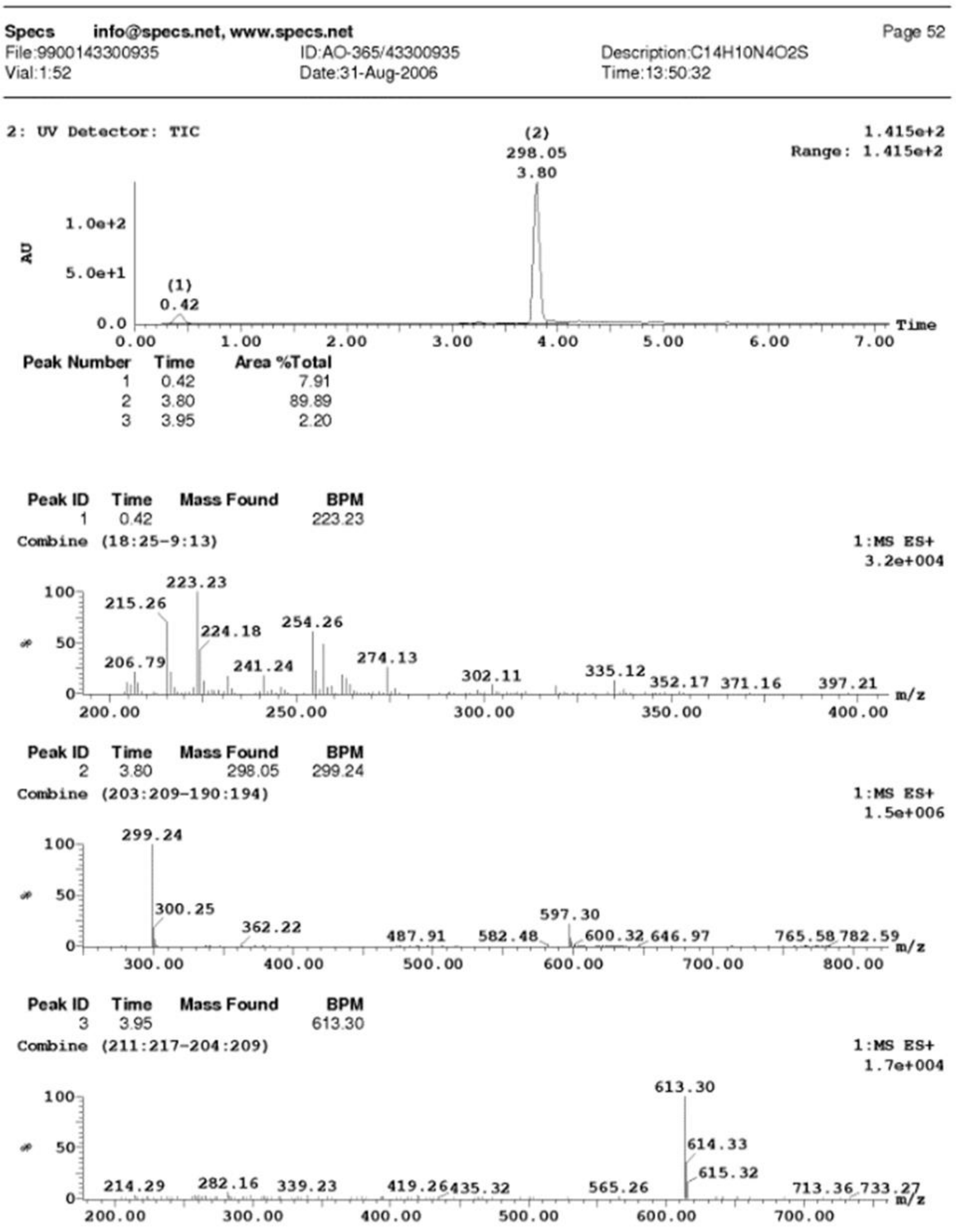

Compound **SC27**, ^{1}H-NMR:

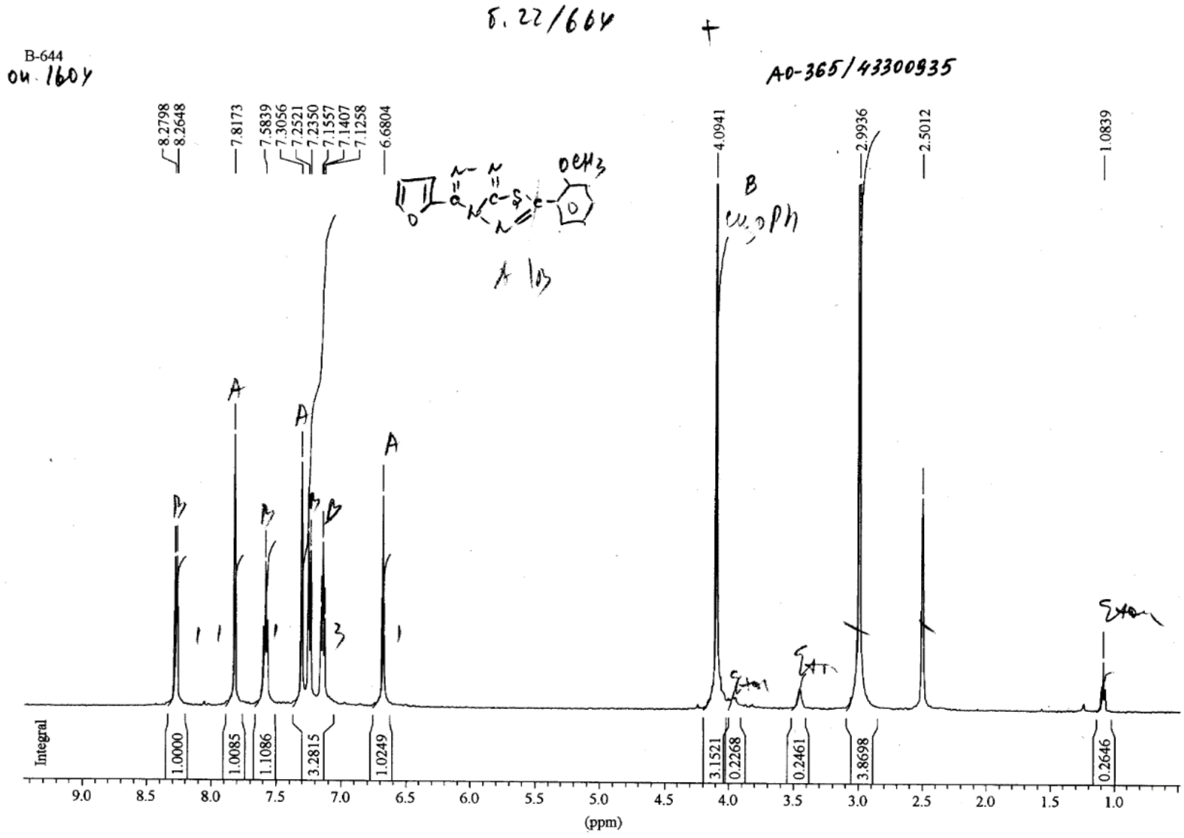

Compound **SC32**, LC-MS:

Specs **info@specs.net, www.specs.net** Page 78

File:9900140720502 ID:AP-263/40720502 Description:C14H11Br2NO4S

Vial:8:5,N Date:24-Sep-2015 Time:16:44:19

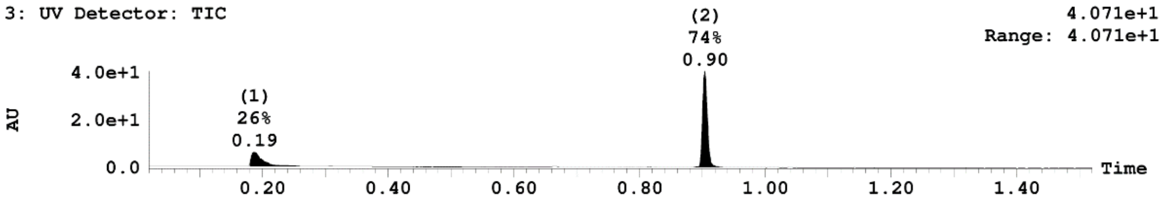

Peak Number	Compound	Time	AreaAbs	Area %Total	Width	Height	Mass Found
1		0.19	1e+005	25.93	0	6e+006	
2	Found	0.90	4e+005	74.07	0	4e+007	446.88

Peak ID	Time	Mass Found	BPM
1	0.19		45.86

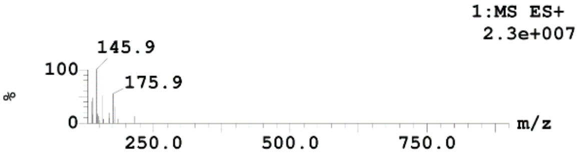

Peak ID	Time	Mass Found	BPM
2	0.90	447.88	49.76

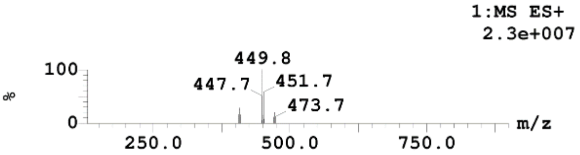

Compound **SC34**, LC-MS:

Specs **info@specs.net, www.specs.net** Page 86
File:9900143418357 ID:AP-263/43418357 Description:C13H11Cl2NO4S
Vial:5:11,F Date:09-Mar-2010 Time:04:53:06

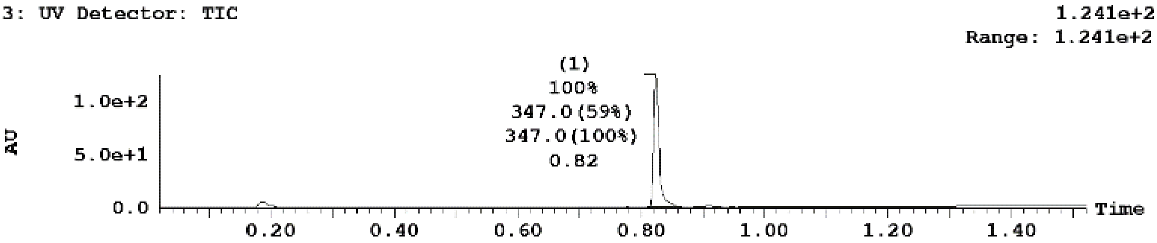

Peak Number	Time	Area %Total
1	0.82	100.00

Peak ID	Time	Mass Found	BPM
1	0.82	346.98	348.27

Peak ID	Time	Mass Found	BPM
1	0.82	346.98	346.24

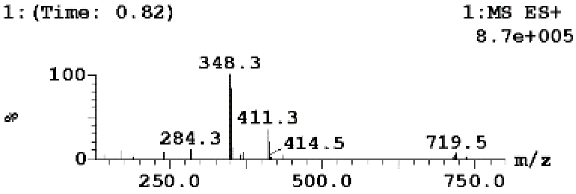

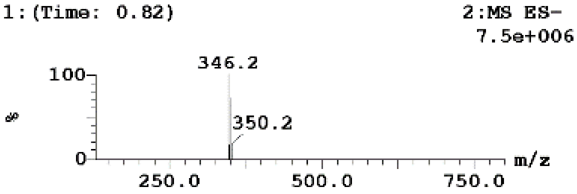

Compound **SC37**, ^{1}H-NMR:

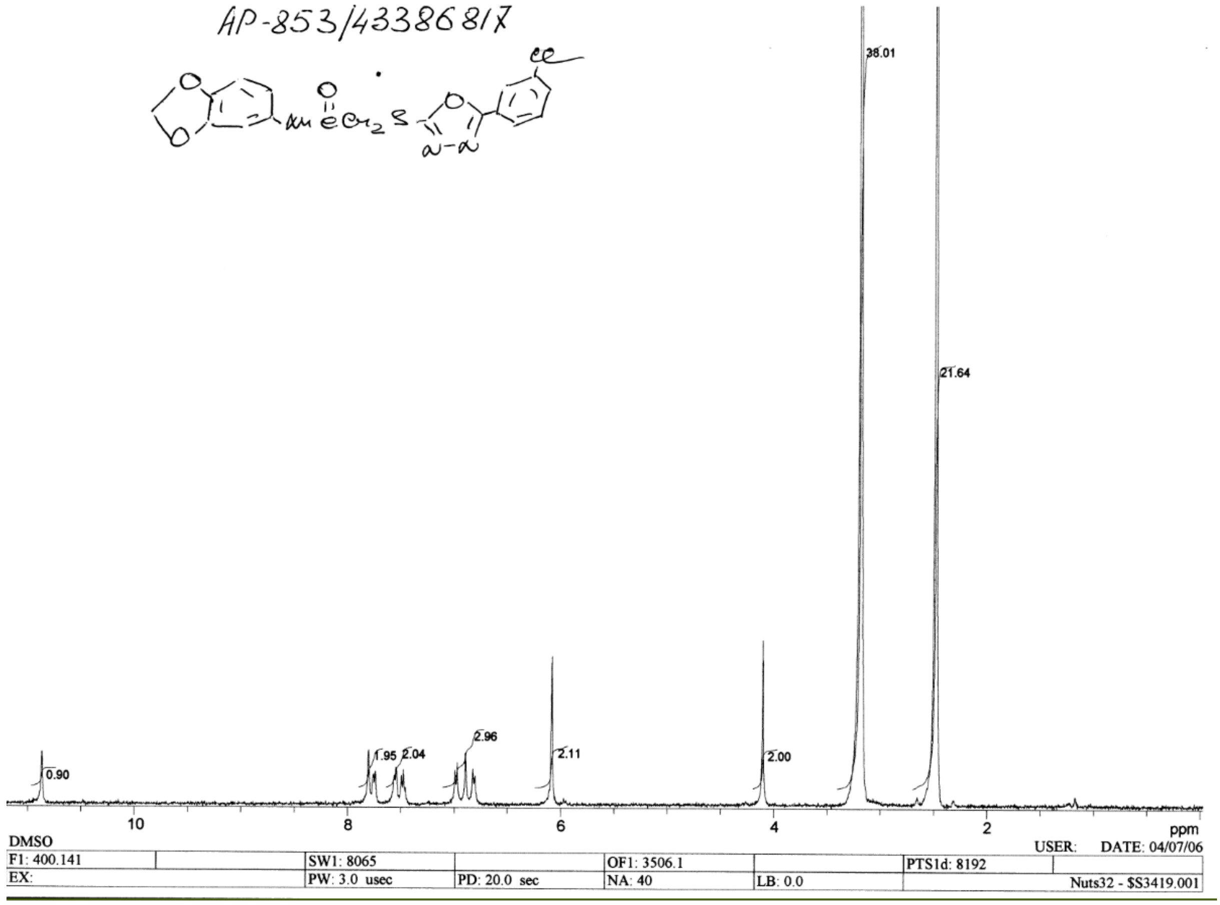

Compound **SC45**, LC-MS:

Specs			Page 11
File:5000112413358	ID:AG-690/12413358	Description:C28H21BrN4O2
Instrument:LC/MS A	Vial:1:11	Date:13-Apr-2004

Printed: Wed Apr 14 13:04:05 2004

Sample Report (continued):

Diode Array Detector 240 - 320 nm: TIC 1.4e+008

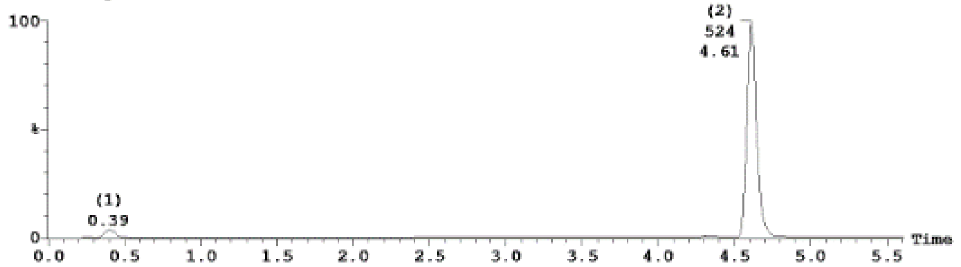

Peak Number	Time	Area %Total
1	0.39	3.80
2	4.61	96.20

Peak ID	Time	Mass Found	BPM
1	0.39		215

Combine (17:23-11:14) 1:MS ES+ 2.0e+003

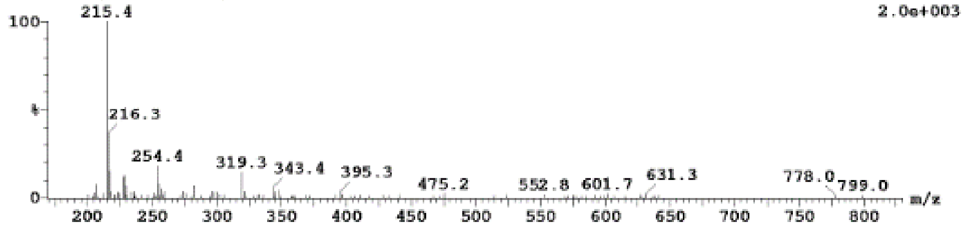

Peak ID	Time	Mass Found	BPM
2	4.61	524.08	525

Combine (247:253-238:241) 1:MS ES+ 9.6e+002

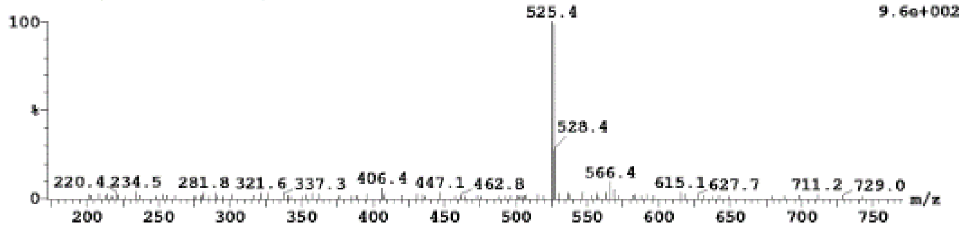

Compound **SC45**, ^{1}H-NMR:

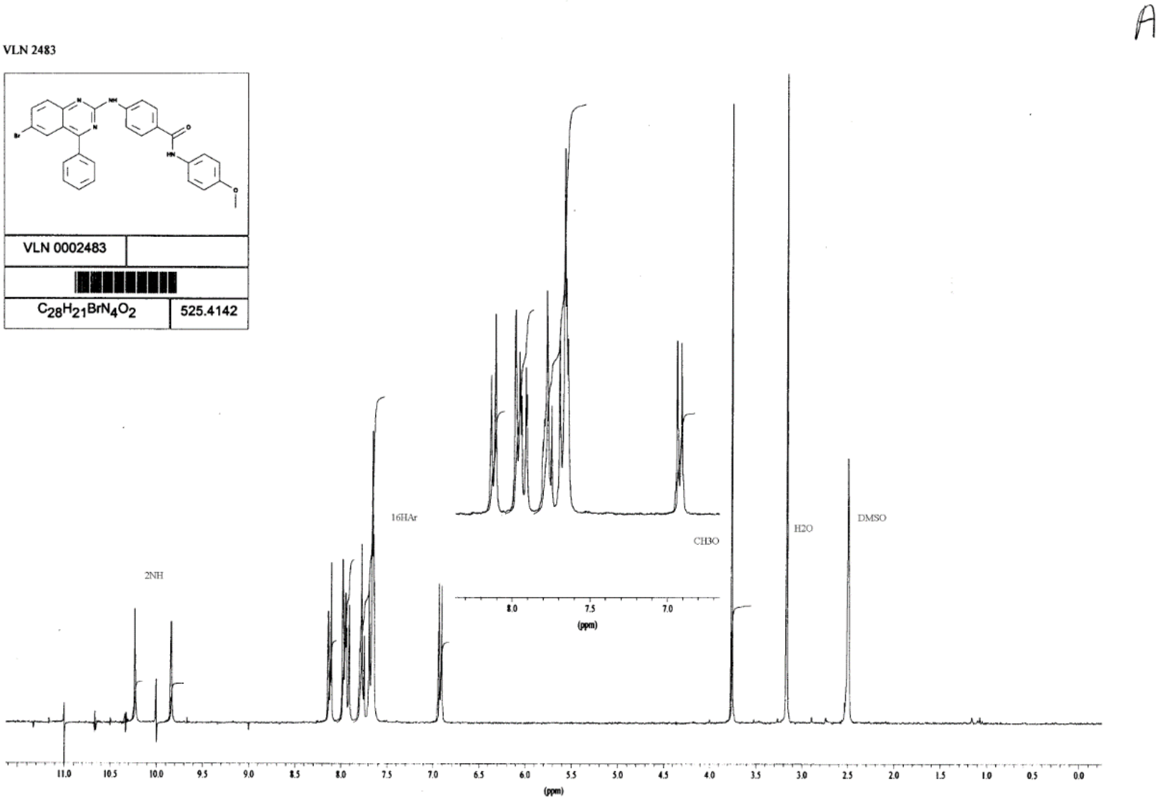

References

1. Zhou, Y.; Xu, J.; Zhu, Y.; Duan, Y.; Zhou, M. Mechanism of Action of the Benzimidazole Fungicide on Fusarium graminearum: Interfering with Polymerization of Monomeric Tubulin But Not Polymerized Microtubule. *Phytopathology.* **2016,** *106*, 807-813. DOI: 10.1094/phyto-08-15-0186-r.

2. Lacey, E.; Brady, R. L.; Prichard, R. K.; Watson, T. R. Comparison of inhibition of polymerisation of mammalian tubulin and helminth ovicidal activity by benzimidazole carbamates. *Vet. Parasitol.* **1987,** *23*, 105-119. DOI: 10.1016/0304-4017(87)90029-X.

3. Morgan, U. M.; Reynoldson, J. A.; Thompson, R. C. Activities of several benzimidazoles and tubulin inhibitors against Giardia spp. in vitro. *Antimicrob. Agents Chemother.* **1993,** *37*, 328-31. DOI: 10.1128/aac.37.2.328.

4. Kamal, A.; Bharath Kumar, G.; Lakshma Nayak, V.; Reddy, V. S.; Shaik, A. B.; Rajender; Kashi Reddy, M. Design, synthesis and biological evaluation of imidazopyridine/imidazopyrimidine-benzimidazole conjugates as potential anticancer agents. *MedChemComm.* **2015,** *6*, 606-612. DOI: 10.1039/C4MD00400K.

5. Wu, Y.; Guan, Q.; Zheng, D.; Yan, P.; Feng, D.; Du, J.; Zhang, J.; Zuo, D.; Bao, K.; Zhang, W. Conformation impacts on the bioactivities of SMART analogues. *Eur. J. Med. Chem.* **2018,** *158*, 733-742. DOI: 10.1016/j.ejmech.2018.09.045.

6. Wang, F.; Yang, Z.; Liu, Y.; Ma, L.; Wu, Y.; He, L.; Shao, M.; Yu, K.; Wu, W.; Pu, Y.; Nie, C.; Chen, L. Synthesis and biological evaluation of diarylthiazole derivatives as antimitotic and antivascular agents with potent antitumor activity. *Bioorg. Med. Chem.* **2015,** *23*, 3337-3350. DOI: 10.1016/j.bmc.2015.04.055.

7. Zhang, Q.; Zhai, S.; Li, L.; Li, X.; Zhou, H.; Liu, A.; Su, G.; Mu, Q.; Du, Y.; Yan, B. Anti-tumor selectivity of a novel Tubulin and HSP90 dual-targeting inhibitor in non-small cell lung cancer models. *Biochem. Pharmacol.* **2013,** *86*, 351-360. DOI: 10.1016/j.bcp.2013.05.019.

8. Li, L.; Liu, Y.; Zhang, Q.; Zhou, H.; Zhang, Y.; Yan, B. Comparison of Cancer Cell Survival Triggered by Microtubule Damage after Turning Dyrk1B Kinase On and Off. *ACS Chem. Biol.* **2014,** *9*, 731-742. DOI: 10.1021/cb4005589.

9. Mu, Y.; Liu, Y.; Li, L.; Tian, C.; Zhou, H.; Zhang, Q.; Yan, B. The novel tubulin polymerization inhibitor MHPT exhibits selective anti-tumor activity against rhabdomyosarcoma in vitro and in vivo. *PLoS One.* **2015,** *10*, e0121806. DOI: 10.1371/journal.pone.0121806.

10. Lawrence, N. J.; Rennison, D.; McGown, A. T.; Ducki, S.; Gul, L. A.; Hadfield, J. A.; Khan, N. Linked Parallel Synthesis and MTT Bioassay Screening of Substituted Chalcones. *J. Comb. Chem.* **2001,** *3*, 421-426. DOI: 10.1021/cc000075z.

11. Woods, J. A.; Hadfield, J. A.; Pettit, G. R.; Fox, B. W.; McGown, A. T. The interaction with tubulin of a series of stilbenes based on combretastatin A-4. *Br. J. Cancer.* **1995,** *71*, 705-711. DOI: 10.1038/bjc.1995.138.

12. Qian, Y.; Ma, G.-Y.; Yang, Y.; Cheng, K.; Zheng, Q.-Z.; Mao, W.-J.; Shi, L.; Zhao, J.; Zhu, H.-L. Synthesis, molecular modeling and biological evaluation of dithiocarbamates as novel antitubulin agents. *Bioorg. Med. Chem.* **2010,** *18*, 4310-4316. DOI: 10.1016/j.bmc.2010.04.091.

13. Salum, L. B.; Altei, W. F.; Chiaradia, L. D.; Cordeiro, M. N. S.; Canevarolo, R. R.; Melo, C. P. S.; Winter, E.; Mattei, B.; Daghestani, H. N.; Santos-Silva, M. C.; Creczynski-

Pasa, T. B.; Yunes, R. A.; Yunes, J. A.; Andricopulo, A. D.; Day, B. W.; Nunes, R. J.; Vogt, A. Cytotoxic 3,4,5-trimethoxychalcones as mitotic arresters and cell migration inhibitors. *Eur. J. Med. Chem.* **2013,** *63*, 501-510. DOI: 10.1016/j.ejmech.2013.02.037.

14. Wang, Y.-T.; Qin, Y.-J.; Zhang, Y.-L.; Li, Y.-J.; Rao, B.; Zhang, Y.-Q.; Yang, M.-R.; Jiang, A.-Q.; Qi, J.-L.; Zhu, H.-L. Synthesis, biological evaluation, and molecular docking studies of novel chalcone oxime derivatives as potential tubulin polymerization inhibitors. *RSC Adv.* **2014,** *4*, 32263-32275. DOI: 10.1039/C4RA03803G.

15. Zhu, C.; Zuo, Y.; Wang, R.; Liang, B.; Yue, X.; Wen, G.; Shang, N.; Huang, L.; Chen, Y.; Du, J.; Bu, X. Discovery of Potent Cytotoxic Ortho-Aryl Chalcones as New Scaffold Targeting Tubulin and Mitosis with Affinity-Based Fluorescence. *J. Med. Chem.* **2014,** *57*, 6364-6382. DOI: 10.1021/jm500024v.

16. Chen, J.; Yan, J.; Hu, J.; Pang, Y.; Huang, L.; Li, X. Synthesis, biological evaluation and mechanism study of chalcone analogues as novel anti-cancer agents. *RSC Adv.* **2015,** *5*, 68128-68135. DOI: 10.1039/C5RA14888J.

17. Yan, J.; Chen, J.; Zhang, S.; Hu, J.; Huang, L.; Li, X. Synthesis, Evaluation, and Mechanism Study of Novel Indole-Chalcone Derivatives Exerting Effective Antitumor Activity Through Microtubule Destabilization in Vitro and in Vivo. *J. Med. Chem.* **2016,** *59*, 5264-5283. DOI: 10.1021/acs.jmedchem.6b00021.

18. Ducki, S.; Rennison, D.; Woo, M.; Kendall, A.; Chabert, J. F. D.; McGown, A. T.; Lawrence, N. J. Combretastatin-like chalcones as inhibitors of microtubule polymerization. Part 1: Synthesis and biological evaluation of antivascular activity. *Bioorg. Med. Chem.* **2009,** *17*, 7698-7710. DOI: 10.1016/j.bmc.2009.09.039.

19. Ducki, S.; Mackenzie, G.; Lawrence, N. J.; Snyder, J. P. Quantitative Structure−Activity Relationship (5D-QSAR) Study of Combretastatin-like Analogues as Inhibitors of Tubulin Assembly. *J. Med. Chem.* **2005,** *48*, 457-465. DOI: 10.1021/jm049444m.

20. Thomopoulou, P.; Sachs, J.; Teusch, N.; Mariappan, A.; Gopalakrishnan, J.; Schmalz, H.-G. New Colchicine-Derived Triazoles and Their Influence on Cytotoxicity and Microtubule Morphology. *ACS Med. Chem. Lett.* **2016,** *7*, 188-191. DOI: 10.1021/acsmedchemlett.5b00418.

21. Cifuentes, M.; Schilling, B.; Ravindra, R.; Winter, J.; Janik, M. E. Synthesis and biological evaluation of B-ring modified colchicine and isocolchicine analogs. *Bioorg. Med. Chem. Lett.* **2006,** *16*, 2761-2764. DOI: 10.1016/j.bmcl.2006.02.010.

22. Cosentino, L.; Redondo-Horcajo, M.; Zhao, Y.; Santos, A. R.; Chowdury, K. F.; Vinader, V.; Abdallah, Q. M. A.; Abdel-Rahman, H.; Fournier-Dit-Chabert, J.; Shnyder, S. D.; Loadman, P. M.; Fang, W.-s.; Díaz, J. F.; Barasoain, I.; Burns, P. A.; Pors, K. Synthesis and Biological Evaluation of Colchicine B-Ring Analogues Tethered with Halogenated Benzyl Moieties. *J. Med. Chem.* **2012,** *55*, 11062-11066. DOI: 10.1021/jm301151t.

23. Singh, B.; Kumar, A.; Joshi, P.; Guru, S. K.; Kumar, S.; Wani, Z. A.; Mahajan, G.; Hussain, A.; Qazi, A. K.; Kumar, A.; Bharate, S. S.; Gupta, B. D.; Sharma, P. R.; Hamid, A.; Saxena, A. K.; Mondhe, D. M.; Bhushan, S.; Bharate, S. B.; Vishwakarma, R. A. Colchicine derivatives with potent anticancer activity and reduced P-glycoprotein induction liability. *Org. Biomol. Chem.* **2015,** *13*, 5674-5689. DOI: 10.1039/C5OB00406C.

24. Romagnoli, R.; Baraldi, P. G.; Salvador, M. K.; Preti, D.; Aghazadeh Tabrizi, M.; Brancale, A.; Fu, X.-H.; Li, J.; Zhang, S.-Z.; Hamel, E.; Bortolozzi, R.; Basso, G.; Viola, G. Synthesis and Evaluation of 1,5-Disubstituted Tetrazoles as Rigid Analogues of

Combretastatin A-4 with Potent Antiproliferative and Antitumor Activity. *J. Med. Chem.* **2012,** *55*, 475-488. DOI: 10.1021/jm2013979.

25. Flynn, B. L.; Gill, G. S.; Grobelny, D. W.; Chaplin, J. H.; Paul, D.; Leske, A. F.; Lavranos, T. C.; Chalmers, D. K.; Charman, S. A.; Kostewicz, E.; Shackleford, D. M.; Morizzi, J.; Hamel, E.; Jung, M. K.; Kremmidiotis, G. Discovery of 7-Hydroxy-6-methoxy-2-methyl-3-(3,4,5-trimethoxybenzoyl)benzo[b]furan (BNC105), a Tubulin Polymerization Inhibitor with Potent Antiproliferative and Tumor Vascular Disrupting Properties. *J. Med. Chem.* **2011,** *54*, 6014-6027. DOI: 10.1021/jm200454y.

26. Sanghai, N.; Jain, V.; Preet, R.; Kandekar, S.; Das, S.; Trivedi, N.; Mohapatra, P.; Priyadarshani, G.; Kashyap, M.; Das, D.; Satapathy, S. R.; Siddharth, S.; Guchhait, S. K.; Kundu, C. N.; Bharatam, P. V. Combretastatin A-4 inspired novel 2-aryl-3-arylamino-imidazo-pyridines/pyrazines as tubulin polymerization inhibitors, antimitotic and anticancer agents. *MedChemComm.* **2014,** *5*, 766-782. DOI: 10.1039/C3MD00357D.

27. Yan, J.; Pang, Y.; Chen, J.; Sheng, J.; Hu, J.; Huang, L.; Li, X. Synthesis, biological evaluation and mechanism study of a class of cyclic combretastatin A-4 analogues as novel antitumour agents. *RSC Adv.* **2015,** *5*, 98527-98537. DOI: 10.1039/C5RA19270F.

28. De Martino, G.; La Regina, G.; Coluccia, A.; Edler, M. C.; Barbera, M. C.; Brancale, A.; Wilcox, E.; Hamel, E.; Artico, M.; Silvestri, R. Arylthioindoles, Potent Inhibitors of Tubulin Polymerization. *J. Med. Chem.* **2004,** *47*, 6120-6123. DOI: 10.1021/jm049360d.

29. De Martino, G.; Edler, M. C.; La Regina, G.; Coluccia, A.; Barbera, M. C.; Barrow, D.; Nicholson, R. I.; Chiosis, G.; Brancale, A.; Hamel, E.; Artico, M.; Silvestri, R. New Arylthioindoles: Potent Inhibitors of Tubulin Polymerization. 2. Structure−Activity

Relationships and Molecular Modeling Studies. *J. Med. Chem.* **2006,** *49*, 947-954. DOI: 10.1021/jm050809s.

30. La Regina, G.; Sarkar, T.; Bai, R.; Edler, M. C.; Saletti, R.; Coluccia, A.; Piscitelli, F.; Minelli, L.; Gatti, V.; Mazzoccoli, C.; Palermo, V.; Mazzoni, C.; Falcone, C.; Scovassi, A. I.; Giansanti, V.; Campiglia, P.; Porta, A.; Maresca, B.; Hamel, E.; Brancale, A.; Novellino, E.; Silvestri, R. New Arylthioindoles and Related Bioisosteres at the Sulfur Bridging Group. 4. Synthesis, Tubulin Polymerization, Cell Growth Inhibition, and Molecular Modeling Studies. *J. Med. Chem.* **2009,** *52*, 7512-7527. DOI: 10.1021/jm900016t.

31. Colley, H. E.; Muthana, M.; Danson, S. J.; Jackson, L. V.; Brett, M. L.; Harrison, J.; Coole, S. F.; Mason, D. P.; Jennings, L. R.; Wong, M.; Tulasi, V.; Norman, D.; Lockey, P. M.; Williams, L.; Dossetter, A. G.; Griffen, E. J.; Thompson, M. J. An Orally Bioavailable, Indole-3-glyoxylamide Based Series of Tubulin Polymerization Inhibitors Showing Tumor Growth Inhibition in a Mouse Xenograft Model of Head and Neck Cancer. *J. Med. Chem.* **2015,** *58*, 9309-9333. DOI: 10.1021/acs.jmedchem.5b01312.

32. Wen, Z.; Xu, J.; Wang, Z.; Qi, H.; Xu, Q.; Bai, Z.; Zhang, Q.; Bao, K.; Wu, Y.; Zhang, W. 3-(3,4,5-Trimethoxyphenylselenyl)-1H-indoles and their selenoxides as combretastatin A-4 analogs: Microwave-assisted synthesis and biological evaluation. *Eur. J. Med. Chem.* **2015,** *90*, 184-194. DOI: 10.1016/j.ejmech.2014.11.024.

33. Diao, P. C.; Li, Q.; Hu, M.-J.; Ma, Y.-F.; You, W.-W.; Hong, K. H.; Zhao, P.-L. Synthesis and biological evaluation of novel indole-pyrimidine hybrids bearing morpholine and thiomorpholine moieties. *Eur. J. Med. Chem.* **2017,** *134*, 110-118. DOI: 10.1016/j.ejmech.2017.04.011.

34. La Regina, G.; Bai, R.; Rensen, W.; Coluccia, A.; Piscitelli, F.; Gatti, V.; Bolognesi, A.; Lavecchia, A.; Granata, I.; Porta, A.; Maresca, B.; Soriani, A.; Iannitto, M. L.; Mariani, M.; Santoni, A.; Brancale, A.; Ferlini, C.; Dondio, G.; Varasi, M.; Mercurio, C.; Hamel, E.; Lavia, P.; Novellino, E.; Silvestri, R. Design and Synthesis of 2-Heterocyclyl-3-arylthio-1H-indoles as Potent Tubulin Polymerization and Cell Growth Inhibitors with Improved Metabolic Stability. *J. Med. Chem.* **2011,** *54*, 8394-8406. DOI: 10.1021/jm2012886.

35. La Regina, G.; Bai, R.; Rensen, W. M.; Di Cesare, E.; Coluccia, A.; Piscitelli, F.; Famiglini, V.; Reggio, A.; Nalli, M.; Pelliccia, S.; Da Pozzo, E.; Costa, B.; Granata, I.; Porta, A.; Maresca, B.; Soriani, A.; Iannitto, M. L.; Santoni, A.; Li, J.; Miranda Cona, M.; Chen, F.; Ni, Y.; Brancale, A.; Dondio, G.; Vultaggio, S.; Varasi, M.; Mercurio, C.; Martini, C.; Hamel, E.; Lavia, P.; Novellino, E.; Silvestri, R. Toward Highly Potent Cancer Agents by Modulating the C-2 Group of the Arylthioindole Class of Tubulin Polymerization Inhibitors. *J. Med. Chem.* **2013,** *56*, 123-149. DOI: 10.1021/jm3013097.

36. La Regina, G.; Bai, R.; Coluccia, A.; Famiglini, V.; Pelliccia, S.; Passacantilli, S.; Mazzoccoli, C.; Ruggieri, V.; Verrico, A.; Miele, A.; Monti, L.; Nalli, M.; Alfonsi, R.; Di Marcotullio, L.; Gulino, A.; Ricci, B.; Soriani, A.; Santoni, A.; Caraglia, M.; Porto, S.; Da Pozzo, E.; Martini, C.; Brancale, A.; Marinelli, L.; Novellino, E.; Vultaggio, S.; Varasi, M.; Mercurio, C.; Bigogno, C.; Dondio, G.; Hamel, E.; Lavia, P.; Silvestri, R. New Indole Tubulin Assembly Inhibitors Cause Stable Arrest of Mitotic Progression, Enhanced Stimulation of Natural Killer Cell Cytotoxic Activity, and Repression of Hedgehog-Dependent Cancer. *J. Med. Chem.* **2015,** *58*, 5789-5807. DOI: 10.1021/acs.jmedchem.5b00310.

37. MacDonough, M. T.; Strecker, T. E.; Hamel, E.; Hall, J. J.; Chaplin, D. J.; Trawick, M. L.; Pinney, K. G. Synthesis and biological evaluation of indole-based, anti-cancer agents inspired by the vascular disrupting agent 2-(3′-hydroxy-4′-methoxyphenyl)-3-(3″,4″,5″-

trimethoxybenzoyl)-6-methoxyindole (OXi8006). *Bioorg. Med. Chem.* **2013,** *21*, 6831-6843. DOI: 10.1016/j.bmc.2013.07.028.

38. Owa, T.; Yokoi, A.; Yamazaki, K.; Yoshimatsu, K.; Yamori, T.; Nagasu, T. Array-Based Structure and Gene Expression Relationship Study of Antitumor Sulfonamides Including N-[2-[(4-Hydroxyphenyl)amino]-3-pyridinyl]-4-methoxybenzenesulfonamide and N-(3-Chloro-7-indolyl)-1,4-benzenedisulfonamide. *J. Med. Chem.* **2002,** *45*, 4913-4922. DOI: 10.1021/jm0201060.

39. Owa, T.; Okauchi, T.; Yoshimatsu, K.; Sugi, N. H.; Ozawa, Y.; Nagasu, T.; Koyanagi, N.; Okabe, T.; Kitoh, K.; Yoshino, H. A focused compound library of novel N-(7-indolyl)benzenesulfonamides for the discovery of potent cell cycle inhibitors. *Bioorg. Med. Chem. Lett.* **2000,** *10*, 1223-1226. DOI: 10.1016/S0960-894X(00)00219-5.

40. Yokoi, A.; Kuromitsu, J.; Kawai, T.; Nagasu, T.; Hata Sugi, N.; Yoshimatsu, K.; Yoshino, H.; Owa, T. Profiling Novel Sulfonamide Antitumor Agents with Cell-based Phenotypic Screens and Array-based Gene Expression Analysis. *Mol. Cancer. Ther.* **2002,** *1*, 275-286.

41. Chang, J.-Y.; Hsieh, H.-P.; Chang, C.-Y.; Hsu, K.-S.; Chiang, Y.-F.; Chen, C.-M.; Kuo, C.-C.; Liou, J.-P. 7-Aroyl-aminoindoline-1-sulfonamides as a Novel Class of Potent Antitubulin Agents. *J. Med. Chem.* **2006,** *49*, 6656-6659. DOI: 10.1021/jm061076u.

42. Wang, Y.-M.; Hu, L.-X.; Liu, Z.-M.; You, X.-F.; Zhang, S.-H.; Qu, J.-R.; Li, Z.-R.; Li, Y.; Kong, W.-J.; He, H.-W.; Shao, R.-G.; Zhang, L.-R.; Peng, Z.-G.; Boykin, D. W.; Jiang, J.-D. N-(2,6-Dimethoxypyridine-3-yl)-9-Methylcarbazole-3-Sulfonamide as a Novel Tubulin Ligand against Human Cancer. *Clin. Cancer. Res.* **2008,** *14*, 6218-6227. DOI: 10.1158/1078-0432.Ccr-08-0550.

43. Shan, B.; Medina, J. C.; Santha, E.; Frankmoelle, W. P.; Chou, T. C.; Learned, R. M.; Narbut, M. R.; Stott, D.; Wu, P.; Jaen, J. C.; Rosen, T.; Timmermans, P. B.; Beckmann, H. Selective, covalent modification of beta-tubulin residue Cys-239 by T138067, an antitumor agent with in vivo efficacy against multidrug-resistant tumors. *Proc. Natl. Acad. Sci. U.S.A.* **1999,** *96*, 5686-91. DOI: 10.1073/pnas.96.10.5686.

44. Reddy, M. V. R.; Mallireddigari, M. R.; Pallela, V. R.; Cosenza, S. C.; Billa, V. K.; Akula, B.; Subbaiah, D. R. C. V.; Bharathi, E. V.; Padgaonkar, A.; Lv, H.; Gallo, J. M.; Reddy, E. P. Design, Synthesis, and Biological Evaluation of (E)-N-Aryl-2-arylethenesulfonamide Analogues as Potent and Orally Bioavailable Microtubule-Targeted Anticancer Agents. *J. Med. Chem.* **2013,** *56*, 5562-5586. DOI: 10.1021/jm400575x.

45. Popowycz, F.; Schneider, C.; DeBonis, S.; Skoufias, D. A.; Kozielski, F.; Galmarini, C. M.; Joseph, B. Synthesis and antiproliferative evaluation of pyrazolo[1,5-a]-1,3,5-triazine myoseverin derivatives. *Bioorg. Med. Chem.* **2009,** *17*, 3471-3478. DOI: 10.1016/j.bmc.2009.03.007.

46. Moon, H.-S.; Jacobson, E. M.; Khersonsky, S. M.; Luzung, M. R.; Walsh, D. P.; Xiong, W.; Lee, J. W.; Parikh, P. B.; Lam, J. C.; Kang, T.-W.; Rosania, G. R.; Schier, A. F.; Chang, Y.-T. A Novel Microtubule Destabilizing Entity from Orthogonal Synthesis of Triazine Library and Zebrafish Embryo Screening. *J. Am. Chem. Soc.* **2002,** *124*, 11608-11609. DOI: 10.1021/ja026720i.

47. Chang, Y.-T.; Wignall, S. M.; Rosania, G. R.; Gray, N. S.; Hanson, S. R.; Su, A. I.; Merlie, J.; Moon, H.-S.; Sangankar, S. B.; Perez, O.; Heald, R.; Schultz, P. G. Synthesis and Biological Evaluation of Myoseverin Derivatives: Microtubule Assembly Inhibitors. *J. Med. Chem.* **2001,** *44*, 4497-4500. DOI: 10.1021/jm010451+.

48. Kryštof, V.; Moravcová, D.; Paprskářová, M.; Barbier, P.; Peyrot, V.; Hlobilková, A.; Havlíček, L.; Strnad, M. Synthesis and biological activity of 8-azapurine and pyrazolo[4,3-d]pyrimidine analogues of myoseverin. *Eur. J. Med. Chem.* **2006,** *41*, 1405-1411. DOI: 10.1016/j.ejmech.2006.07.004.

49. Kuo, S. C.; Lee, H. Z.; Juang, J. P.; Lin, Y. T.; Wu, T. S.; Chang, J. J.; Lednicer, D.; Paull, K. D.; Lin, C. M. Synthesis and cytotoxicity of 1,6,7,8-substituted 2-(4'-substituted phenyl)-4-quinolones and related compounds: identification as antimitotic agents interacting with tubulin. *J. Med. Chem.* **1993,** *36*, 1146-1156. DOI: 10.1021/jm00061a005.

50. Abad, A.; López-Pérez, J. L.; del Olmo, E.; García-Fernández, L. F.; Francesch, A.; Trigili, C.; Barasoain, I.; Andreu, J. M.; Díaz, J. F.; San Feliciano, A. Synthesis and Antimitotic and Tubulin Interaction Profiles of Novel Pinacol Derivatives of Podophyllotoxins. *J. Med. Chem.* **2012,** *55*, 6724-6737. DOI: 10.1021/jm2017573.

51. Labruère, R.; Gautier, B.; Testud, M.; Seguin, J.; Lenoir, C.; Desbène-Finck, S.; Helissey, P.; Garbay, C.; Chabot, G. G.; Vidal, M.; Giorgi-Renault, S. Design, Synthesis, and Biological Evaluation of the First Podophyllotoxin Analogues as Potential Vascular-Disrupting Agents. *ChemMedChem.* **2010,** *5*, 2016-2025. DOI: 10.1002/cmdc.201000305.

52. Prinz, H.; Ishii, Y.; Hirano, T.; Stoiber, T.; Camacho Gomez, J. A.; Schmidt, P.; Düssmann, H.; Burger, A. M.; Prehn, J. H. M.; Günther, E. G.; Unger, E.; Umezawa, K. Novel Benzylidene-9(10H)-anthracenones as Highly Active Antimicrotubule Agents. Synthesis, Antiproliferative Activity, and Inhibition of Tubulin Polymerization. *J. Med. Chem.* **2003,** *46*, 3382-3394. DOI: 10.1021/jm0307685.

53. Zuse, A.; Schmidt, P.; Baasner, S.; Böhm, K. J.; Müller, K.; Gerlach, M.; Günther, E. G.; Unger, E.; Prinz, H. Sulfonate Derivatives of Naphtho[2,3-b]thiophen-4(9H)-one and

9(10H)-Anthracenone as Highly Active Antimicrotubule Agents. Synthesis, Antiproliferative Activity, and Inhibition of Tubulin Polymerization. *J. Med. Chem.* **2007,** *50*, 6059-6066. DOI: 10.1021/jm0708984.

54. Prinz, H.; Schmidt, P.; Böhm, K. J.; Baasner, S.; Müller, K.; Unger, E.; Gerlach, M.; Günther, E. G. 10-(2-oxo-2-Phenylethylidene)-10H-anthracen-9-ones as Highly Active Antimicrotubule Agents: Synthesis, Antiproliferative Activity, and Inhibition of Tubulin Polymerization. *J. Med. Chem.* **2009,** *52*, 1284-1294. DOI: 10.1021/jm801338r.

55. Surkau, G.; Böhm, K. J.; Müller, K.; Prinz, H. Synthesis, antiproliferative activity and inhibition of tubulin polymerization by anthracenone-based oxime derivatives. *Eur. J. Med. Chem.* **2010,** *45*, 3354-3364. DOI: 10.1016/j.ejmech.2010.04.019.

56. Prinz, H.; Schmidt, P.; Böhm, K. J.; Baasner, S.; Müller, K.; Gerlach, M.; Günther, E. G.; Unger, E. Phenylimino-10H-anthracen-9-ones as novel antimicrotubule agents—synthesis, antiproliferative activity and inhibition of tubulin polymerization. *Bioorg. Med. Chem.* **2011,** *19*, 4183-4191. DOI: 10.1016/j.bmc.2011.06.010.

57. Silva-García, E. M.; Cerda-García-Rojas, C. M.; del Río, R. E.; Joseph-Nathan, P. Parvifoline Derivatives as Tubulin Polymerization Inhibitors. *J. Nat. Prod.* **2019,** *82*, 840-849. DOI: 10.1021/acs.jnatprod.8b00860.

58. Wang, Y.; Zhang, H.; Gigant, B.; Yu, Y.; Wu, Y.; Chen, X.; Lai, Q.; Yang, Z.; Chen, Q.; Yang, J. Structures of a diverse set of colchicine binding site inhibitors in complex with tubulin provide a rationale for drug discovery. *FEBS J.* **2016,** *283*, 102-111. DOI: 10.1111/febs.13555.

59. Wang, Q.; Arnst, K. E.; Wang, Y.; Kumar, G.; Ma, D.; White, S. W.; Miller, D. D.; Li, W.; Li, W. Structure-Guided Design, Synthesis, and Biological Evaluation of (2-(1H-Indol-3-

yl)-1H-imidazol-4-yl)(3,4,5-trimethoxyphenyl) Methanone (ABI-231) Analogues Targeting the Colchicine Binding Site in Tubulin. *J. Med. Chem.* **2019,** *62*, 6734-6750. DOI: 10.1021/acs.jmedchem.9b00706.
